# An Entire Process Optimization Strategy for Comprehensive In Vivo Metabolite Profiling of Prucalopride in Rats Based on Ultra-Performance Liquid Chromatography With Q-Exactive Hybrid Quadrupole–Orbitrap High-Resolution Mass Spectrometry

**DOI:** 10.3389/fphar.2021.610226

**Published:** 2021-05-07

**Authors:** Lihua Zuo, Liwei Liu, Yantao Yang, Jie Yang, Min Chen, Huafeng Zhang, Jian Kang, Xiaojian Zhang, Jiabo Wang, Zhi Sun

**Affiliations:** ^1^ Department of Pharmacy, the First Affiliated Hospital of Zhengzhou University, Zhengzhou, China; ^2^ Henan Engineering Research Center of Clinical Mass Spectrometry for Precision Medicine, Zhengzhou, China; ^3^ Department of Neurology, The First Affiliated Hospital of Zhengzhou University, Zhengzhou, China; ^4^ Department of Orthopedics, the First Affiliated Hospital of Zhengzhou University, Zhengzhou, China; ^5^ School of Traditional Chinese Medicine, Capital Medical University, Beijing, China

**Keywords:** prucalopride, metabolite identification, high-resolution mass spectrometry, the entire and novel strategy, metabolic network

## Abstract

Prucalopride was widely used for chronic constipation, which is difficult to be adequately relieved by laxatives in adult patients in clinic. Due to the difficulty in metabolite identification, metabolic process of prucalopride had not been investigated in vivo. In this study, an efficient strategy was proposed for comprehensive metabolite profiling of prucalopride after oral administration in rat plasma, urine, and feces samples. This strategy was composed of five steps. First, the samples at multiple time points after oral administration were collected to increase the representativeness of the samples. Second, different sample preparation methods were investigated to obtain superior extraction efficiency. Third, the raw data of test sample and blank sample were acquired using ultra-performance liquid chromatography with Q-Exactive hybrid quadrupole–orbitrap high-resolution accurate mass spectrometry under the positive and negative full-scan/dd MS^2^ mode. Fourth, combined mass defect filter with background subtraction model in soft of compound discovery, all peaks were constructed to filter potential metabolites after retention time alignment and ion filtration, which could remove large amounts of interference ions. Besides, it can predict potential biotransformation, promoting to understand how to metabolize the drug. This provides multiple possibilities and prevents us conjecturing the potential metabolites blindly. Finally, the verification procedure was implemented through exporting the structure and MS^2^ spectrum to the analytical tool of Mass Frontier. The proposed strategy significantly improved the targeted detection and identification for metabolites in vivo. A total of 47 metabolites were tentatively characterized in the plasma, urine, and feces samples after oral administration of prucalopride. This study could provide a valuable reference for systematic metabolite profile of drug in vivo.

## Introduction

The drug-related metabolites could provide abundant information related to the side effects and formation of active or other toxic metabolites (Balasaheb et al., 2018; Godinho et al., 2018; Radko and Olejnik, 2018; Shankar et al., 2018). However, in vivo samples are very complex with majority of detected peaks in low abundance, which may throw a huge obstacle to acquire purified metabolite-related data from complex materials (Chaleckis et al., 2019). Furthermore, even though the purified data are obtained, the structure elucidation and the annotation of particular biotransformation still are an ongoing challenge (Wolfender et al., 2015; Mahieu and Patti, 2017). Therefore, the efficient strategies that could extract and target detect the multiple trace-absorbed prototypes and metabolites from the complex biological matrix are in great demand.

As one of the leading edge techniques in the drug metabolite research, ultra-high-performance liquid chromatography coupled with mass spectrometry (UHPLC-MS) has developed rapidly in recent decades. It could shorten the analysis time and improve the analysis efficiency by optimizing the parameters of chromatography–mass spectrometry. Meanwhile, there are several scan modes, such as information-dependent acquisition (IDA), selected reaction monitoring (SRM), and precursor and neutral loss scan, which have their own superiorities and characteristics. But due to its drawbacks, including relatively insensitive scan functions, long duty cycle, and multiple injections to monitor multiple precursors and neutral, it was used in the drug-related metabolite studies previously (Gao et al., 2015). While data-independent acquisition (DIA) is more suitable to acquire comprehensive data because they could obtain complete fragment ion information by two interleaved scan functions using gradient collision energy, respectively (Roemmelt et al., 2014; Zhu et al., 2014; Maurer and Meyer, 2016; Zhou and Yin, 2016; Ludwig et al., 2018; Wang et al., 2018; Yao et al., 2018). Although the data acquisition method has improved, what we will be facing is still a heavy workload. Hence, the strategy integrated the acquisition mode, and database and data analysis tools have been proposed. MDF combined with BS are common data processing method, which should select a MDF template indicating that the metabolites without high similarity to the template would not be detected. But the MDF-processed data often display false-positive results and cannot offer sufficient information for the structure (Xing et al., 2015a; Xing et al., 2017; Li et al., 2018a; Zhou et al., 2018). So, the novel ring double bond (RDB; valence values of elements in structure) filter was added to show rich structural information in more sensitive full-scan MS chromatograms. In addition, the combination of neutral loss and precursor ion scan mode was also applied to the explanation of the metabolites, but the systematic and comprehensive metabolite data cannot be obtained with the structure elucidation being time consuming and laborious (Domínguez-Romero et al., 2013; Xing et al., 2015b; Blaženović et al., 2018; Li et al., 2018b).

Prucalopride is the first representative of a novel chemical class of dihydrobenzofurancarboxamide derivatives, which has highly selective agonist activity and high affinity 5-HT_4_ receptor to act primarily on different parts of the lower gastrointestinal tract, promoting cholinergic, nonadrenergic and noncholinergic neurotransmission by enteric neurons (Sun et al., 2016). In our previous study of our group, the pharmacokinetics and tissue distribution of prucalopride had been studied (Sun et al., 2016; Zuo et al., 2016). However, the metabolites of prucalopride in vivo are not clear. So, in this study, the metabolite identification of prucalopride was taken as a case. Forty-seven metabolites were identified in total with some interesting phenomena that found that the drug could combine with some endogenous substances, such as amino acid, palmitic acid, and so on. This study may support the scientific explanation for the activity or toxicity.

**FIGURE 1:**
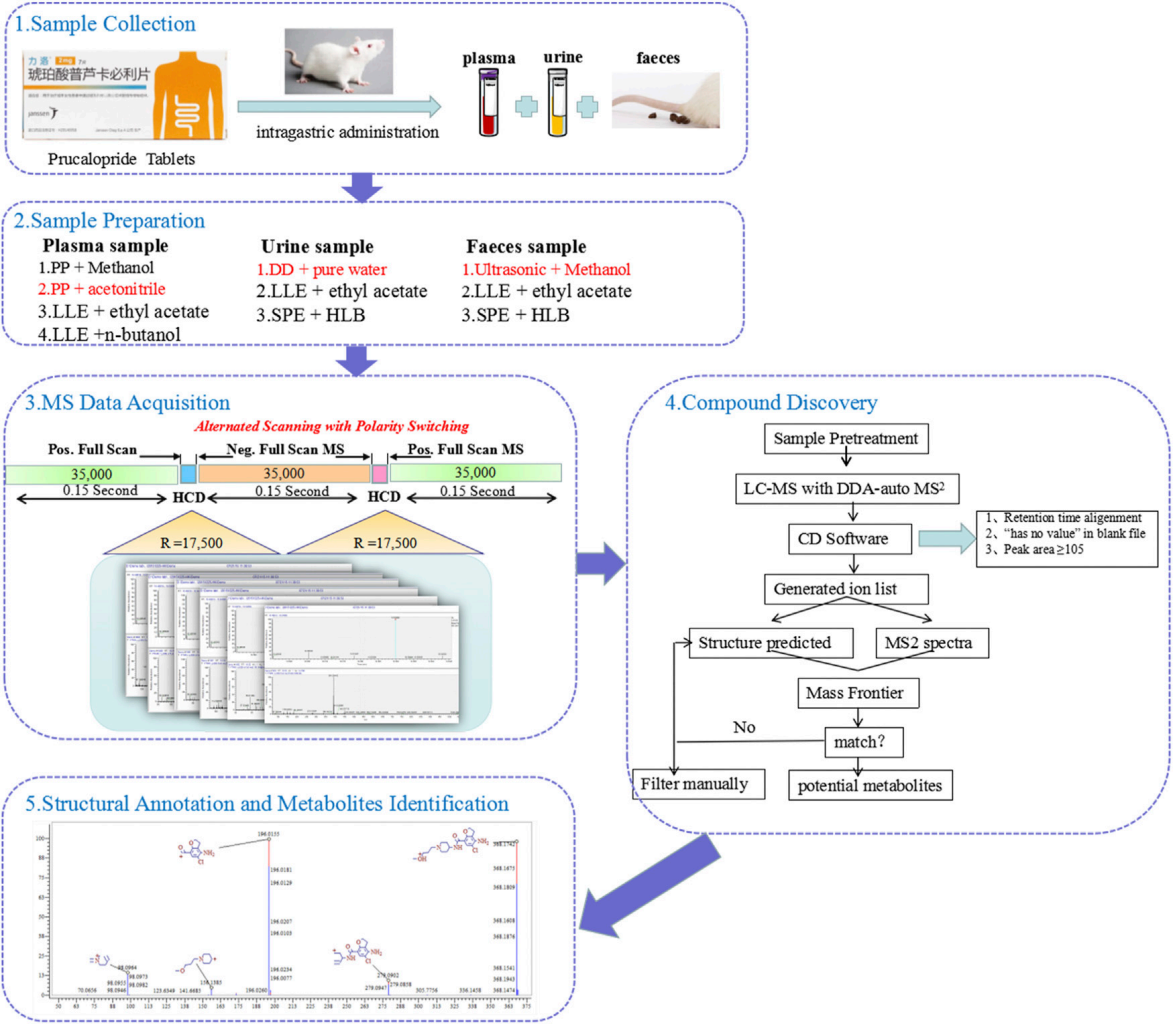
The workflow of analytical strategy for metabolite identification using UHPLC-Q-Orbitrap HRMS in combination with multiple data mining techniques.

In this study, a comprehensive strategy was proposed for the rapid identification of the metabolites of prucalopride after oral administration based on the ultra-performance liquid chromatography with Q-Exactive hybrid quadrupole–orbitrap high-resolution accurate mass spectrometry (UHPLC-HRMS). This strategy could be divided into five steps. The flow chart is shown in Figure 1. First, the plasma, urine, and feces samples at multiple time points after oral administration were collected for analysis. Second, different sample preparation methods were investigated to obtain superior extraction efficiency, including the protein precipitation, liquid–liquid extraction, and solid phase extraction. Third, the MS acquisition method was optimized under the positive and negative full-scan/dd MS^2^ mode. Fourth, combined mass defect filter with background subtraction model in soft of compound discovery, all peaks were constructed to filter potential metabolites after retention time alignment and ion filtration, which could remove large amounts of interference ions. Besides, it can predict potential biotransformation, which could help us in inferring the metabolic process of the drug. This provides multiple possibilities and prevents us conjecturing the potential metabolites blindly. Finally, the verification procedure was implemented through exporting the structure and MS^2^ spectrum to the analytical tool of Mass Frontier. The absorbed metabolites were comprehensively characterized by reference standards and MS/MS fragmentation in rat plasma, urine, and feces after oral administration of prucalopride. The entire strategy presented in this study significantly will improve the targeted detection and identification for metabolites in complex biological samples.

## Experiment and Methods

### Material, Reagents, and Animals

Prucalopride (purity >99%) was obtained from Sigma Chemical Co. (St.Louis, MO, United States). Acetonitrile and methanol of HPLC grade were purchased from Fisher Scientific (Fair Lawn, NJ, United States). The chemical of formic acid was of HPLC grade and purchased from Aladdin Industrial Corporation (Shanghai, China). All other reagents were of analytical grade. Deionized water was purified via a Milli-Q system (Millipore, Milford, MA, United States).

Male Sprague–Dawley (SD) rats, weighing 200 ± 20 g, were acquired from the Experimental Animal Center of Zhengzhou University (Zhengzhou, China). In order to adapt to the environment, all animals were fed under the controlled temperature (20 ± 2°C), humidity (60 ± 5%), and 12-h-light/12-h-dark cycle for one week before the experiment. All protocols of animal experiments were in accordance with the Regulations of Experimental Animal Administration issued by the Animal Ethics Committee of First Affiliated Hospital of Zhengzhou University.

### Chromatographic Conditions

Chromatographic separation was performed on an UHPLC Dionex Ultimate 3,000 (Thermo Scientific, San Jose, United States) equipped with a binary pump solvent management system with online degasser, autosampler, and column oven. A Waters ACQUITY UPLC® BEH C_18_ column (2.1 mm × 100 mm, 1.7 µm) was employed and the column temperature was maintained at 40°C. The mobile phase was composed of A (0.1% formic acid in water) and B (acetonitrile) with the following gradient elution: 0.0–3.0 min, 5%B; 3.0–10.0 min, 5–30%B; 10.0–27.0 min, 30–80% B; 27.0–28.0 min, 80–100% B; 28.0–30.0 min, 100% B; and 30.0–32.0 min, 100–5% B at a flow of 0.2 ml/min. The autosampler was conditioned at 10°C, and the injection volume was 5 µL for analysis.

### Mass Spectrometric Conditions

A Q-Exactive high-resolution mass spectrometry using a heated electrospray ionization (HESI) ion source was tandem to the UHPLC system. The optimized parameters of mass spectrometry were illustrated as below: spray voltage: + 3.5 kV or −2.8 kV; sheath gas pressure: 40 arb; aux gas pressure: 10 arb; sweep gas pressure: 0 arb; capillary temperature: 320°C; auxiliary gas heater temperature: 300°C; S-lens RF level: 50 V; scan mode: full MS/dd-MS^2^, 1) full MS: resolution: 70,000; automatic gain control (AGC) target: 3.0 e^6^; maximum injection time (IT): 200 ms; scan range: 80–1,200 m*/z*; 2) dd-MS/MS^2^: resolution: 17,500; AGC target: 1.0 e5; maximum IT: 50 ms; loop count: five; isolation window: 2.0 m*/z*; NCE/stepped: 20, 30, and 40; and dynamic exclusion: 10.0 s. Nitrogen was used for spray stabilization and as the collision gas in the C-trap. All data collected in profile mode were acquired and processed using Thermo Xcalibur 3.0 software. Regarding the sequence of analysis, all samples were randomized. Blank sample containing barely the solvent was added after every 20 samples, aiming to elute residuals or other impurities which were retained by the chromatography column in the previous spectra records.

### Sample Collection

Blood samples of 300 μL were collected via the orbital vein at 0 (predose); 10 min; 30 min; 1, 2, and 4 h after oral administration of 1.0 mg/kg prucalopride. The heparin sodium was added to exert the anticoagulant action, and the samples were centrifuged at 3,500 rpm for 10 min at 4°C, then all plasma samples were pooled together. Blank urine and feces samples were collected by putting the SD rats into the metabolic cages, and the rats were fasted but drunk freely. The samples after administration were collected the same as the above.

### Sample Preparation

Protein in rat plasma sample (100 µL) was precipitated with the acetonitrile (300 µL). After vortexing for 3 min, the sample was centrifuged at 13,000 rpm for 10 min. Then, the supernatant was transferred into a 1.5-ml centrifuge tube and evaporated to dryness in a vacuum centrifugal concentrator. The residue was dissolved in 50 µL of 50% methanol with vortex mixing for 1 min, and the centrifugation process was the same as the above for 5 min. Finally, 5 µL supernatant was injected into the UHPLC-MS/MS system for analysis.

The urine sample (200 µL) was diluted with 200 µL pure water, vortexing for 1 min. After centrifuging at 13,000 rpm for 10 min, the supernatant was filtered by 0.22 µm MCE microporous filter membrane into sample bottle for analysis.

The feces sample (0.2 g) was weighed precisely into the 4 ml EP tube, adding 2 ml methanol and ultrasonic dissolving, then the supernatant was transferred and centrifuged at 13,000 rpm for 10 min. After that, the supernatant was filtered by 0.22 µm nylon microporous filter membrane into the sample bottle for analysis.

### MS Data Processing

The HRMS data of all samples were processed in the Compound Discoverer 2.1 (CD, Thermo Fisher Scientific) to extract the metabolite-related dataset according to the structural correlation between the drug and its metabolites. Then, the information of blank samples as control from the above dataset was removed by setting “has no value” in blank file after the retention time alignment and database search. And the peak area of ions was equal or greater than 10^5^ in order to differentiate the metabolites from the base peaks. Next, the structure was speculated based on the fragmentation pattern and relative purified ion lists, which possessed the message of molecular formula, molecular weight, retention time, potential biotransfromation, etc. The structure and mass spectrometry were further exported to the Mass Frontier 7.0 (MF, Thermo Fisher Scientific) to observe whether the structure and fragments match.

## Results and Discussion

### Optimization of Sample Preparation

For different samples, the process of sample treatment was investigated. In order to extract and detect more metabolites of prucalopride from plasma samples, we systematically investigated the sample preparation method, including the commonly used protein precipitation (PP) with methanol and acetonitrile and liquid–liquid extraction (LLE) with ethyl acetate and n-butanol. In order to extract and detect more metabolites of prucalopride from urine samples, direct dilution (DD) with pure water, liquid–liquid extraction (LLE) with ethyl acetate, and solid phase extraction (SPE) with Oasis® HLB cartridges were done. In order to extract and detect more metabolites of prucalopride from feces samples, ultrasonic extraction with methanol, liquid–liquid extraction (LLE) with ethyl acetate, and solid phase extraction (SPE) with Oasis® HLB cartridges were done. By comparing the extraction efficiency, the treatment conditions of plasma samples (100 µL) were finally protein precipitation (PP) with acetonitrile (300 µL). Because there were a large number of metabolites in urine and the polarity of metabolites is quite different, the urine sample (200 µL) was diluted with 200 µL pure water in order to ensure that more metabolites could be detected. While for feces samples, the sample preparation was adopted by ultrasonic extraction with methanol.

### Optimization of Chromatographic and Mass Spectrometric Conditions

In order to obtain higher responses, suitable retention behaviors, and symmetrical peak shapes for the analyte, various mobile phase conditions (methanol, acetonitrile, different proportions of formic acid, ammonium acetate, and 100% water) were tested as potential mobile phases. Finally, acetonitrile–water containing 0.1% formic acid was chosen for its good peak shapes. It was also noted that gradient elution could dramatically narrow the peak shape and improve the response intensity and resolution.

For MS parameters optimizing, scans were simultaneously implemented in both positive and negative ion detection mode. In Q-Exactive instrument, there were two modes that could be used to perform an accurate qualitation. They were full MS/dd MS^2^ mode and all-ion fragmentation (AIF). All ions entered into HCD for collision under the AIF mode. Due to no selectivity for parent ions, this data acquisition mode was rarely used. While under the full MS/dd MS^2^ mode, the matching fragment information of primary and secondary mass spectra simultaneously. So the full MS/dd MS^2^ mode was adopted for metabolite profiling of prucalopride in rats. Meanwhile, the other MS parameters, such as the gas pressure, the heated temperature, and the spray voltage, were all well optimized for maximum response. The optimal parameters settings are shown in *Mass Spectrometric Conditions*.

### The Establishment of MDF and BS Model in CD

Metabolite identification, still, is a hard nut to crack at present. There are not only complex endogenous components but also abundant metabolites, which display a high degree of structural variability. The established strategy could solve the matters in the metabolite annotation to a great extent, which was proposed as follows. After the pretreatment, the data extraction was operated with the IDA-based data acquisition, which was based on the retention time alignment, database searching, scoring, etc. Then, the extracted ion lists were filtered by setting parameter in manual. Furthermore, the tentative identification was conducted.

#### Retention Time Alignment and Ion Extraction

After importing the raw files of control and samples into the soft of compound discovery, the alignment algorithm looks for matching features (chromatographic peaks with the same *m/z* × RT dimensions) in the input files. Then, the following parameters such as parent compound or compounds, number of dealkylation and dearylation steps, number and type of transformation steps, and list of possible adduct ions would be set based on the experimenter’s demands and metabolic type to search the metabolites of detected compounds. When the application finds a matching compound, it will mark the pathway for fast search. According to the above, an expected ion list was generated.

#### Matrix Filter and Noise Subtraction

The first step extracts the related data from the complex data, while the annotation of the generated ion list still is a huge project which may contain several thousand potential ion features as well as the interference of the noise and biological matrix. In order to subtract the matrix, the parameter was set with “has no value” in the blank file for screening. Furthermore, considering differentiation of the metabolites with baseline noise, we set the peak area equal or greater than 10^5^ or other ranges according to the actual requirement. By filtering, the resulting list information will be significantly reduced, which will reduce unnecessary work and improve the efficiency.

### Structural Annotation and Metabolites Identification

Structural elucidation is time consuming and labor-intensive process to manually interpret spectra; thus, the powerful analytical software is considered with its unique new features to significantly improve our workflow. Mass Frontier (MF) can offer a number of enhancements designed to increase productivity. The generated ion list after the data handling procedure from CD contains the messages of molecular formula, molecular weight, retention time, and potential transformation. And in the same window interface, the mass spectrometry is presented along with the corresponding compound information and the parent drug is used as the reference in the bottom of the mass spectrometry with the corresponding compound in the upper, which is convenient for the structure fragment alignment. Therefore, we can deduce the metabolites’ structure by fragmentation regulation of mass spectra and transformation suggested. Then, the mass spectra called out from the Xcalibur 3.0 is exported into the MF, which automatically generated fragments and detailed fragmentation and rearrangement mechanisms from the chemical structure. If the theoretical fragmentation through the MF could match the MS^2^ spectrogram, the compound was identified tentatively; if not, the ion feature was filtered manually.

### Comprehensive Characterization of Metabolites of Prucalopride *in vivo*

In this study, the plasma, urine, and feces were collected for the metabolite identification. The raw data files containing experiment group and control group were uploaded into the CD, a total of 2,717, 5,869, and 5,310 ion were extracted in the plasma, urine, and feces samples with the software running, respectively. Then, the remaining ions were handled further, just 340, 777, and 300 ions ultimately left. According to suggested transformation pathways, molecular formula, and mass spectrometry fragment ions to predict the underlying metabolite structures which can be confirmed by MF in turn, we identified 47 tentative metabolites in total. All the information of 47 metabolites of prucalopride detected by UHPLC-Q-Orbitrap HRMS is listed in Table 1. And Supplementary Figure S1 shows the extraction flow chromatography and MS/MS spectrum of prucalopride and metabolites.

**TABLE 1:** All information of 47 metabolites of prucalopride detected and annotated by UHPLC-Q-Orbitrap HRMS.

No	Formula	RT (min)	Ion mode	ES/expected (m/z)	ES/measured (m/z)	Delta (ppm)	Intensity (E)	Assignment	Fragmention (m/z)
M_0_	C_18_H_26_ClN_3_O_3_	9.787	+H	368.17354	368.17361	0.174	1.31^7^		368.17, 279.09, 196.02, 173.16, 156.14, 98.10
M_1_	C11H12N2O4	2.256	+H	237.08698	237.08664	−1.448	1.39^6^	−(C7 H14 Cl N) +(O)	237.08, 220.06, 202.05, 192.07, 174.05, 164.07, 150.05, 146.06, 136.08, 120.08, 180.07, 94.07
M_2_	C14H27N3O3	1.363	+H	286.21251	286.21222	−1.042	1.26^6^	−(C4 Cl) +(H)	286.21, 144.10, 84.08, 70.07
M_3_	C_14_H_18_ClN_3_O_2_	8.051	+H	296.11603	296.11578	−0.848	2.47^6^	−(C_4_ H_8_ O)	296.11, 279.09, 213.04, 196.01, 104.04, 84.08
M_4_	C17H24ClN3O3	8.500	+H	354.15789	354.15756	−0.948	2.71^6^	−(C H2)	354.16, 279.09, 196.02, 142.12, 124.11, 98.10
M_5_	C17H22ClN3O4	8.597	+H	368.13716	368.13687	−0.789	1.61^6^	−(C H4) +(O)	368.14, 308.12, 196.02, 156.10, 96.08
M_6_	C17H24ClN3O4	6.949	+H	370.15281	370.15259	−0.596	1.34^6^	−(C H2) +(O)	370.15, 295.08, 212.01, 194.00, 142.12, 98.10, 81.07, 69.07
M_7_	C18H24ClN3O4	9.432	+H	382.15281	382.15256	−0.655	1.15^6^	−(H2) +(O)	382.15, 350.13, 293.07, 209.99, 173.16, 156.14, 98.10, 70.07
M_8_	C17H22ClN3O5	6.871	+H	384.13207	384.13174	−0.872	2.94^6^	−(C H4) +(O2)	384.13, 366.12, 324.11, 212.01, 194.00, 156.10, 96.08
M_9_	C_18_H_26_ClN_3_O_4_	8.336	+H	384.16846	384.16791	−1.433	1.61^6^	+(O)	384.17, 295.08, 212.01, 194.00, 156.13, 98.10
M_10_	C_14_H_16_ClN_3_O_2_	8.280	+H	294.10038	294.09991	−1.602	2.92^7^	−(C_4_ H_10_ O)	294.10, 221.87, 194.00, 84.08
M_11_	C_14_H_18_ClN_3_O_2_	8.071	+H	296.11603	296.11600	−0.105	3.29^8^	−(C_4_ H_8_ O)	296.11, 279.09, 213.04, 196.01, 104.04, 84.08
M_12_	C_14_H_16_ClN_3_O_3_	7.559	+H	310.09529	310.09479	−1.631	4.56^7^	−(C_4_ H_10_)	310.09, 293.06, 227.02, 209.99, 101.10, 84.08, 82.06, 70.06
M_13_	C_14_H_16_ClN_3_O_4_	8.097	+H	326.09021	326.08896	−3.834	5.47^6^	−(C_4_ H_10_) +(O)	326.09, 229.04, 211.02, 212.01, 194.00, 98.06
M_14_	C_18_H_27_N_3_O_3_	9.711	+H	334.21251	334.21198	−1.610	3.61^6^	−(Cl) +(H)	334.21, 245.13, 162.06, 98.10, 81.07, 69.07
M_15_	C_18_H_27_N_3_O_4_	8.162	+H	350.20743	350.20676	−1.921	4.78^6^	−(Cl) +(H O)	350.20, 261.12, 178.05, 146.06, 98.10
M_16_	C_17_H_22_ClN_3_O_3_	8.720	+H	352.14224	352.14188	−1.039	8.38^7^	−(C H_4_)	352.13, 277.07, 194.00, 159.15, 142.12, 98.10, 70.07
M_17_	C_17_H_20_ClN_3_O_4_	8.825	+H	366.12151	366.12003	−4.043	8.89^7^	−(C H_6_) +(O)	366.12, 306.10, 263.06, 194.00, 156.10, 114.09, 102.06, 96.08
M_18_	C_18_H_24_ClN_3_O_3_	9.990	+H	366.15789	366.15756	−0.917	2.18^8^	−(H_2_)	366.16, 277.07, 194.00, 173.06, 156.14, 124.11, 98.10, 70.07
M_19_	C_17_H_22_ClN_3_O_4_	8.560	+H	368.13716	368.13693	−0.626	1.65^8^	−(C H_4_) +(O)	368.13, 213.04, 196.02, 138.09, 98.10, 86.06, 70.07
M_20_	C_17_H_24_ClN_3_O_4_	6.900	+H	370.15281	370.15237	−1.190	1.44^8^	−(C H_2_) +(O)	370.15, 352.14, 295.08, 212.01, 194.00, 159.15, 142.12, 98.10, 70.07
M_21_	C_18_H_24_ClN_3_O_4_	9.366	+H	382.15281	382.15237	−1.153	2.73^8^	−(H_2_) +(O)	382.15, 293.07, 209.99, 173.16, 156.14, 141.14, 98.10, 70.07
M_22_	C_17_H_22_ClN_3_O_5_	6.859	+H	384.13207	384.13162	−1.184	3.65^8^	−(C H_4_) +(O_2_)	384.13, 366.12, 324.11, 295.08, 212.01, 194.00, 156.10, 96.08
M_23_	C_18_H_26_ClN_3_O_4_	8.201	+H	384.16846	384.16785	−1.589	2.89^9^	+(O)	384.16, 352.14, 295.08, 212.01, 194.01, 173.16, 166.00, 156.13, 98.09, 70.06
M_24_	C_18_H_26_ClN_3_O_4_	10.101	+H	384.16846	384.16800	−1.199	2.28^9^	+(O)	384.17, 352.14, 308.12, 279.09, 196.02, 154.12, 96.08, 70.07
M_25_	C_18_H_22_ClN_3_O_5_	8.825	+H	396.13207	396.13153	−1.376	3.79^7^	−(H_4_) +(O_2_)	396.13, 307.04, 223.97, 173.16, 156.14, 141.14, 98.10, 70.07
M_26_	C_18_H_24_ClN_3_O_5_	9.578	+H	398.14772	398.14603	−4.257	9.06^6^	−(H_2_) +(O_2_)	398.15, 309.06,2 93.07, 209.99194.00, 154.12, 96.08, 70.07
M_27_	C_18_H_24_ClN_3_O_5_	7.990	+H	398.14772	398.14740	−−0.816	9.06^6^	−(H_2_) +(O_2_)	398.15, 380.14, 291.05, 225.09, 173.16, 156.14, 98.10, 70.07
M_28_	C_19_H_25_N_3_O_4_	10.303	+K	398.14766	398.14911	3.630	9.06^6^	−(H Cl) +(C O)	398.14, 266.09, 182.05, 98.09, 85.02, 73.02, 70.06, 69.03, 67.05
M_29_	C_17_H_22_ClN_3_O_6_	7.532	+H	400.12698	400.12634	−1.623	2.97^7^	−(C H_4_) +(O_3_)	400.13, 382.11, 328.10, 295.08, 212.02, 194.00, 154.09, 94.07
M_30_	C_18_H_26_ClN_3_O_5_	9.447	+H	400.16337	400.16278	−1.487	3.84^6^	+(O_2_)	400.17, 311.08, 228.01, 209.99, 173.16, 173.17, 98.10, 73.03
M_31_	C_18_H_26_ClN_3_O_5_	9.280	+H	400.16337	400.16302	−0.887	3.84^6^	+(O_2_)	400.17, 311.08, 251.06, 213.12, 196.02, 89.06, 73.03
M_32_	C_20_H_28_ClN_3_O_4_	8.600	+H	410.18411	410.18387	−0.586	1.50^7^	+(C_2_ H_2_ O)	410.18, 321.10, 238.03, 196.02, 156.14, 98.10, 70.07
M_33_	C_18_H_24_ClN_3_O_6_	7.896	+H	414.14263	414.14157	−2.583	1.00^6^	−(H_2_) +(O_3_)	414.14, 370.15, 197.10, 173.16, 156.14, 98.10, 70.07
M_34_	C_24_H_50_N_2_O_3_	11.117	+K	453.34530	453.34296	−5.166	5.17^6^	−(Cl N) +(C_6_ H_24_)	453.35, 435.33, 322.25, 277.18, 28.16, 209.17, 114.09, 96.08, 69.07
M_35_	C_24_H_34_ClN_3_O_9_	7.315	+H	544.20563	544.20508	−1.018	5.86^6^	+(C_6_ H_8_ O_6_)	544.21, 455.13, 410.18, 372.05, 196.02, 156.14, 98.10, 70.07
M_36_	C_24_H_32_ClN_3_O_10_	7.088	+H	558.18489	558.18420	−0.698	5.87^6^	+(C_6_ H_6_ O_7_)	558.18, 382.15, 293.07, 173.16, 156.14, 98.10, 85.03
M_37_	C_24_H_34_ClN_3_O_10_	8.253	+H	560.20054	560.19952	−1.836	9.62^5^	+(C_6_ H_8_ O_7_)	560.18, 368.17, 196.02, 156.14, 98.10, 70.07
M_38_	C_13_H_21_N_3_O_4_	1.009	+H	284.16048	284.16068	−1.487	1.58^6^	−(C_5_ H_5_ Cl) +(O)	284.16, 267.14,255. 13, 225.12, 209.09, 156.07,142.09, 116.07, 114.07, 95.06, 84.08, 72.08, 70.07
M_39_	C_14_H_18_ClN_3_O_2_	8.052	+H	296.11603	296.11591	−0.105	9.17^5^	−(C_4_ H_8_ O)	296.11, 279.09, 213.04, 196.01, 104.04, 84.08
M_40_	C_18_H_24_ClN_3_O_3_	9.987	+H	366.15789	366.15744	−1.245	4.09^6^	−(H_2_)	366.16, 301.18, 277.07, 194.00, 173.16, 156.14, 98.10, 81.07, 69.07
M_41_	C_17_H_20_ClN_3_O_4_	8.813	+H	366.12151	366.12103	−1.312	1.62^6^	−(C H6) +(O)	366.12, 306.10, 277.07, 194.00, 156.10, 96.08
M_42_	C_17_H_22_ClN_3_O_4_	8.568	+H	368.13716	368.13687	−0.789	4.86^6^	−(C H_4_) +(O)	368.13, 308.12, 279.09, 265.07, 196.02, 156.10
M_43_	C_17_H_24_ClN_3_O_4_	6.956	+H	370.15281	370.15262	−0.515	1.22^6^	−(C H_2_) +(O)	370.15, 324.19, 295.08, 212.01, 194.00, 142.12, 98.10, 93.08
M_44_	C_18_H_24_ClN_3_O_4_	9.413	+H	382.15281	382.15192	−2.33	1.64^6^	−(H2) +(O)	382.15, 293.07, 209.99, 173.16, 156.14, 98.10, 70.07
M_45_	C_17_H_22_ClN_3_O_5_	6.868	+H	384.13207	384.13156	−1.341	2.06^6^	−(C H_4_) +(O_2_)	384.13, 366.12, 324.11, 212.01, 194.00, 156.10, 136.08
M_46_	C_18_H_26_ClN_3_O_4_	8.323	+H	384.16846	384.16791	−1.433	3.52^7^	+(O)	384.17, 295.08, 212.01, 194.00, 173.16, 156.14, 124.11, 98.10
M_47_	C_18_H_26_ClN_3_O_4_	7.880	+H	384.16846	384.16800	−1.199	3.52^7^	+(O)	384.17, 295.08, 212.01, 194.00, 156.14, 98.10

#### Identification of Metabolites in Rat Plasma

**FIGURE 2:**
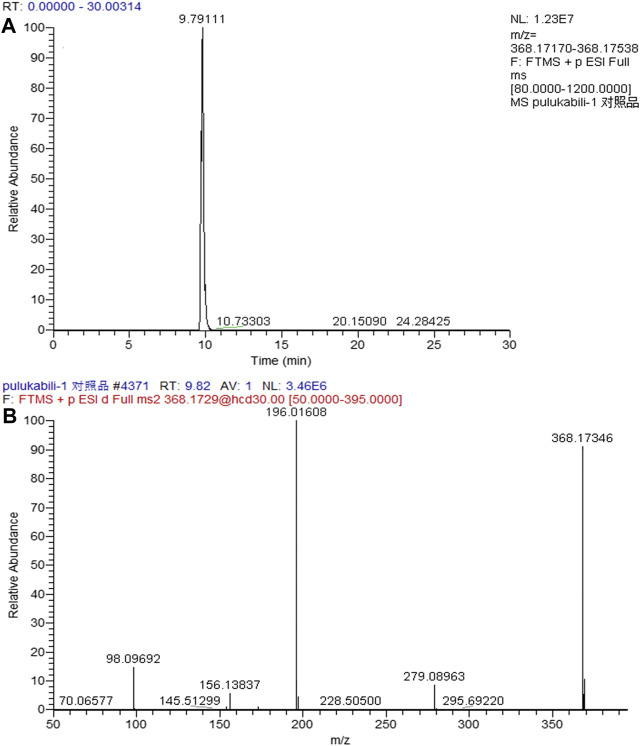
The chromatogram **(A)** and mass spectrogram **(B)** of prucalopride in positive ion mode.

In the positive ion mode, the ion flow chromatograph of prucalopride reference (C_18_H_26_ClN_3_O_3_) was extracted, and the excimer ion peak of *m/z* 368.17354 [M + H]^+^ was generated (t_*R*_ = 9.978 min, error: 0.174 ppm), as shown in Figure 2.

**FIGURE 3:**
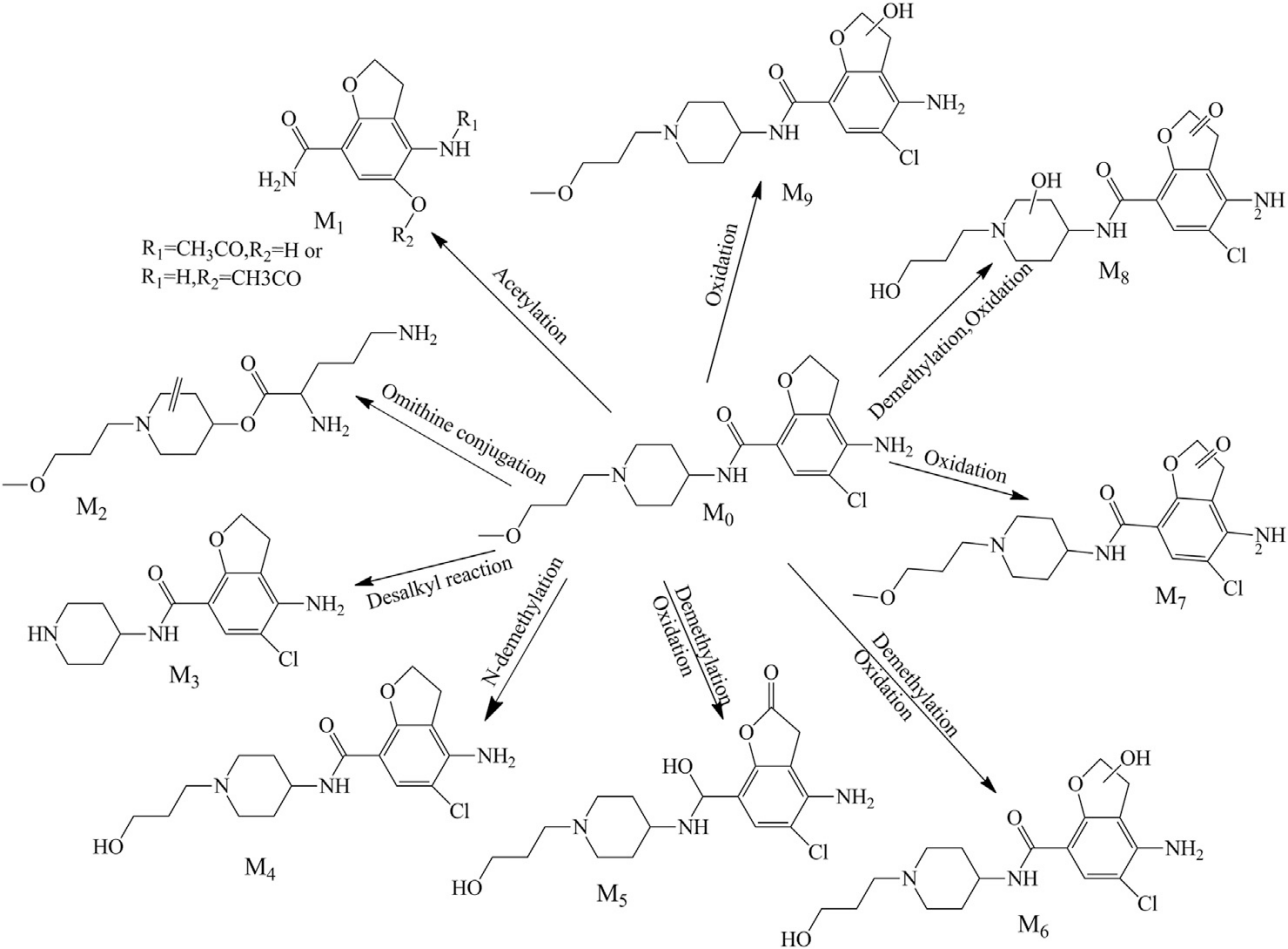
Proposed metabolite pathway of prucalopride in the plasma.

There were nine metabolites (M_1_-M_9_; Figure 3) identified tentatively in the plasma with the information summarized in Table 1. Metabolites M_1_ and M_2_ mainly occurred the break of bond in the “N” linked with piperidine ring, M_1_ (a part of prucalopride with benzofuran ring) was the ion at *m/z* 237.08664, which is the product of acetylation with the reaction site in amino group or hydroxyl group, and M_2_ was detected at 1.363 min with *m/z* 286.21222, which was the other part of parent drug and bound with ornithine in vivo. The fitted chemical formula of M_3_ was C_14_H_18_ClN_3_O_2_, a difference of C_4_H_8_O with prucalopride which indicated the loss of alkyl chain moiety, and the characteristic ions were in accordance with the speculative structure. The M_4_ was found at 8.500 with *m/z* 354.15756 (with a mass shift of 14.02 Da, just lost of a methyl group), so this metabolite showed the characteristic fragments with *m/z* 279.09, 196.02, and 98.10. M_5_ and M_6_ (*m/z* 368.13687 and *m/z* 370.15259) were the oxidation products in piperidine ring or furan ring with the methyl group leaving. M_7_ presented at 9.432 min with *m/z* 382.15256, and only oxidation in furan ring was happened; thus, this metabolite had common fragments with the parent drug, *m/z* 173.16, 156.14, 98.10, and 70.07. M_8_ was yielded via oxidation in piperidine ring and furan ring and demethylation of the alkyl chain. Metabolite M_9_ was the oxidation product in the furan ring and presented precursor ion *m/z* 384.16846 with RT 8.336 min. Due to the tiny structural changes, the characteristic fragment ions (*m/z* 173.16, 156.13, 98.09, and 70.06) were detected.

#### Identification of Metabolites in Rat Urine

**FIGURE 4:**
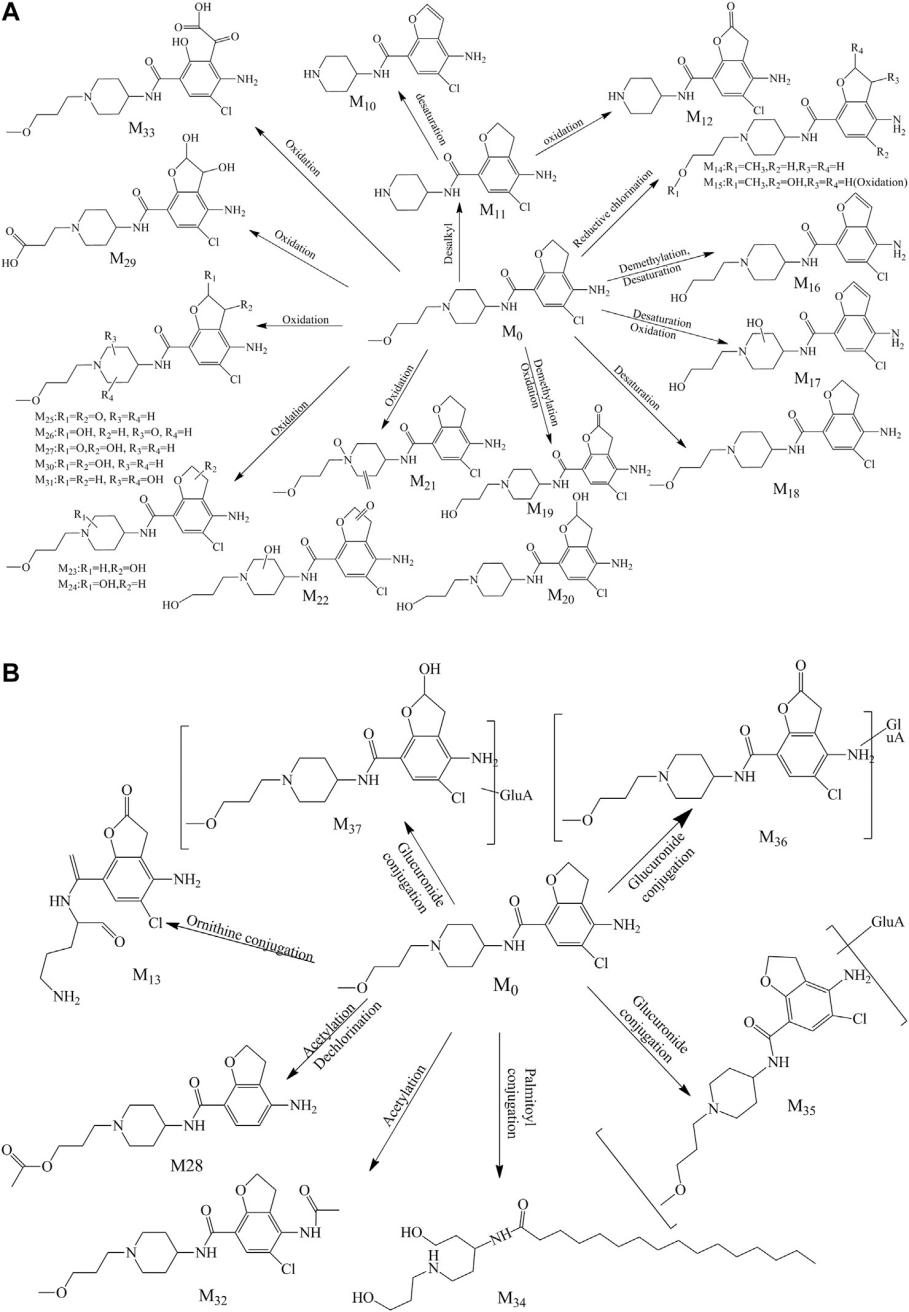
**(A)** Proposed phase I metabolite pathway of prucalopride in the urine. **(B)** Proposed phase II metabolite pathway of prucalopride in the urine.

A total of 28 metabolites (M_10_–M_37_; Figures 4A,B) were found in the urine which involved complex metabolic reaction. Metabolites M_10_–M_12_ (RT 8.280, 8.071, and 7.559 min) were detected as the leaving of the alkyl chain moiety with *m/z* 294.09991, 296.11600, and 310.09479, respectively. And, compared with the metabolite M_11_, M_10_ took off two hydrogen atoms to generate a double bond in furan ring, and M_12_ was oxidized in the furan ring. Metabolite M_13_ (RT 8.097, *m/z* 326.08896) was also the result of the ornithine conjugation, but different from the metabolite M_2_, the ornithine was combined with the acylamino group of the other side of the drug structure. The parent drug prucalopride was a chlorine-containing compound; thus, oxidative dechlorination and reductive dechlorination happened when it was metabolized and yielded the metabolite M_14_ and M_15_ at 9.711 and 8.162 min with *m/z* 334.21198 and 350.20676.

Metabolite M_16_ was detected at 8.720 min with the mass charge ratio 352.14188. And the fitted molecular formula was C_17_H_22_ClN_3_O_3_, just a difference of 2H with the M_4_ (C_17_H_24_ClN_3_O_3_). The typical fragment ions (*m/z* 352.13, 277.07, and 194.00) that indicated the part of furan ring had a deviation of 2 Da with the metabolite M_4_ suggesting that M_16_ lost 2H from the furan ring in the basis of M_4A_.

Metabolites M_17_, M_19_, and M_20_ were the products of the demethylation and were found at 8.825, 8.560, and 6.900 min with *m/z* 366.12003, 368.13693, and 370.15237, while oxidation or dehydrogenation also took place in different positions of the piperidine ring or furan ring. Metabolite M_18_ just took off two hydrogen atoms in the basis of parent drug; therefore, *m/z* 277.07 (a mass shift of 2.02 Da, dehydrogenated from the ion *m/z* 279.09), 194.00 (a mass shift of 2.02 Da, dehydrogenated from the ion *m/z* 156.02), 173.06, 156.14, 98.10, and 70.07 were detected which were the typical ion fragments of the prucalopride.

The oxidation in the “N” of piperidine ring also happened to generate metabolite M_21_ (RT 9.366 and *m/z* 382.15237), with oxidation, dehydrogenation, or demethylation in other regions. M_22_ was found at 6.859 min with *m/z* 384.13162, and this compound might be the same substance with the M8 according to the comparison of MS^2^ spectrum. And this assumption was confirmed by the MF. M_23_ was the oxidation product in the furan ring and presented precursor ion *m/z* 384.16785 with RT 8.201 min. The mass charge ratio and retention time were approaching between M_9_ and M_23_ which suggested that they might possess the same structure. The MS^2^ spectra of both were analogous and the result from MF confirmed the above assumption. M_24_ was the isomer of M_23_ with the oxidation position in the piperidine ring and exhibited precursor ion *m/z* 384.16800 with RT 10.101 min. And it showed the characteristic fragment ions (*m/z* 173.16, 156.13, 98.09, and 70.06).

Metabolites M_25_, M_26_, M_27_, M_30_, and M_31_ all possessed two oxidation sites in piperidine ring or furan ring which was a kind of main metabolic pathway of the parent molecule, their mass charge ratios were 396.13153, 398.14740, 398.14603, 400.16302, and 400.16278, and the retention time was 8.825, 9.578, 7.990, 9.447, and 9.280, respectively. In the basis of M_30_, the demethylation and oxidation in the alkyl side chain happen to emerge M_29_, which was found at 7.532 min with *m/z* 400.12634. The [M + K] ion of the metabolite M_28_ was *m/z* 398.14911 and was detected at 10.303 min, the acetylation occurred with the hydroxyl group after the demethylation with the hydrogen atom dropping out in furan ring and chlorine atom leaving away the benzene ring through reductive dechlorination.

M_32_ was an acetylated product of the parent drug, combined with the amino group of benzene ring and detected at the 8.600 min with the *m/z* 410.18387. Typical fragments (*m/z* 196.02, 156.14, 98.10, and 70.07) were found in the MS^2^ of this metabolite. M_33_ (*m/z* 414.14157, RT 7.896) was a ring-opening compound with the furan ring opening and oxidation conducted. M_34_ was an interesting metabolite which was a palmitic acid derivative exhibited at 11.117 min with *m/z* 453.34296. Such a way to metabolize was rare in the current literature reports and united closely with the endogenous substances. Therefore, the discovery of a metabolic mode like this was meaningful for the metabolite exploration.

The metabolites of M_35_, M_36_, and M_37_ were the phase II metabolites identified as glucuronic acid conjugates preliminarily. The detailed information was summarized in Table 1. The molecular weight of M_35_ was 176 Da more than the parent drug, it was a glucuronic acid conjugate obviously. The fitted molecular formulas for M_36_ and M_37_ were C_24_H_32_ClN_3_O_10_ and C_24_H_34_ClN_3_O_10_, indicating that an oxidation happened in the basis of M_35_ (C_24_H_34_ClN_3_O_9_). And the structure was exported to the MF to confirm the matching between the structure and the MS^2^ spectrum, and the outcome corroborated our speculation.

#### Identification of Metabolites in Rat Feces Samples

**FIGURE 5:**
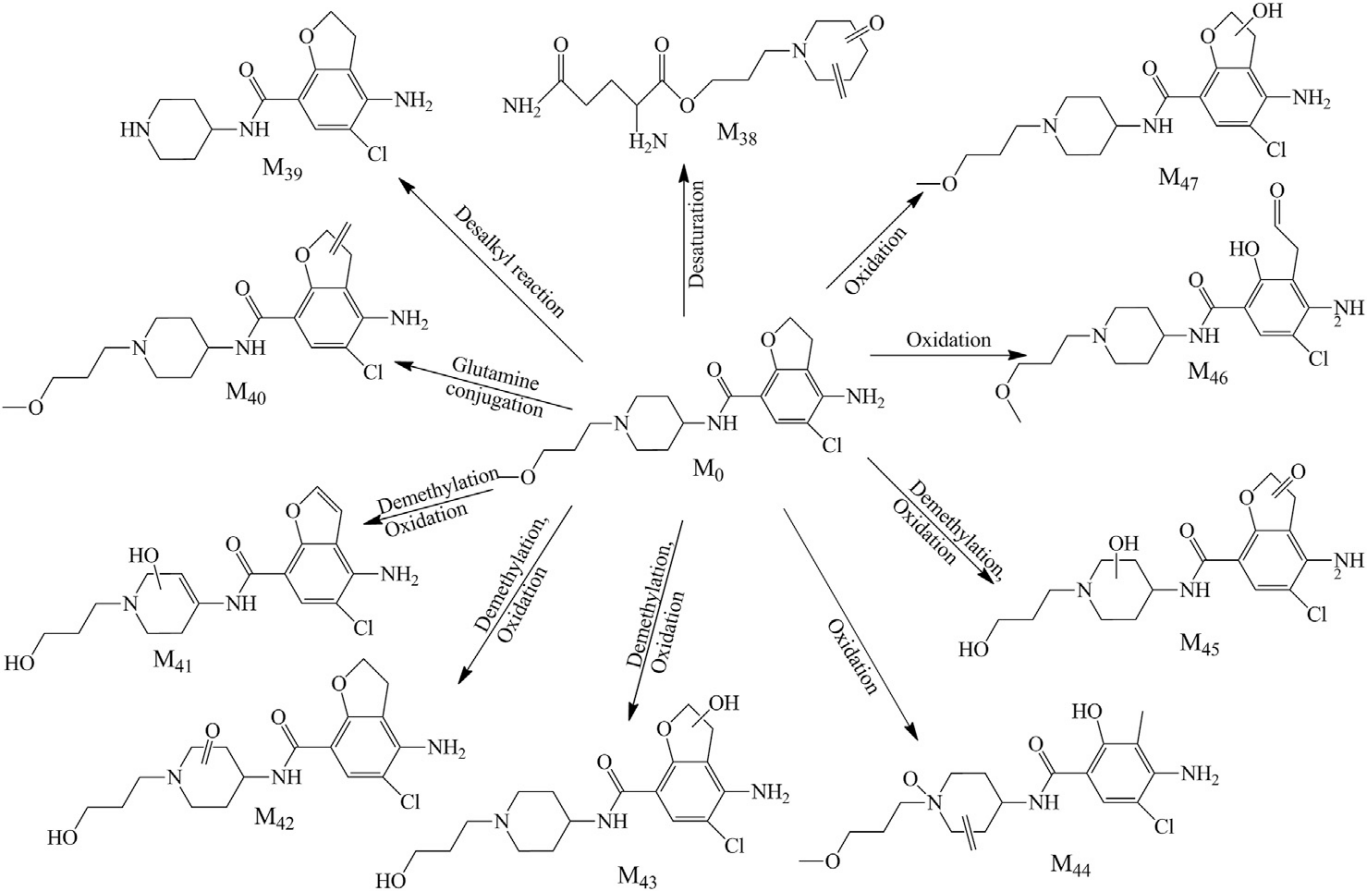
Proposed metabolite pathway of prucalopride in the feces.

In the feces samples, 10 metabolites (M_38_–M_47_, Figure 5) were found in total. Metabolite 38 was the glutamine conjugate combined with the part of alkyl chain, this metabolite was found at 1.009 min with *m/z* 284.16068. M_39_ was detected at 8.052 with *m/z* 296.11591. The retention time and mass charge ratio were close to the M_3_, and the MS^2^ spectrum was analogous; thus, we assumed that the M_39_ and M_3_ were the same substance. Metabolite M_40_ (RT 9.987, *m/z* 366.15744) was the reactive product of dehydrogenation and generated typical fragment ions *m/z* 194.00, 173.16, 156.14, and 98.10. The fitted molecular formula for metabolite M_41_ was C_17_H_20_ClN_3_O_4,_ which was found in the urine (M_17_) as well. Through the comparison of both, M_41_ was considered as the same substance with M_17_. M_42_ and M_43_ were yielded *via* demethylation and oxidation in different sites, with RT 8.568 and 6.956 min and *m/z* 368.13687 and 370.15262, separately. The metabolite M_44_ was C_18_H_24_ClN_3_O_4_, and this metabolite was detected in the plasma and urine as well. The retention time and MS^2^ spectrum were matching; thus, these metabolites were the same substance. M_45_ was detected at 6.868 min with *m/z* 384.13156. This compound was generated through the demethylation and oxidation in the piperidine ring or furan ring which was found in the plasma and urine as well. The precursor ion of M_46_ was *m/z* 384.16791 along with RT 8.323, the hydroxylation reaction happened in the furan ring, and the fragment ions (*m/z* 194.00, 173.16, 156.14, and 98.10) were detected as the diagnostic ions. M_47_ was the result of the oxidative ring–opening reaction, with RT 7.880 min and *m/z* 384.16800. The detailed information is showed in Table 1.

### Metabolite Profiling of Prucalopride

**FIGURE 6:**
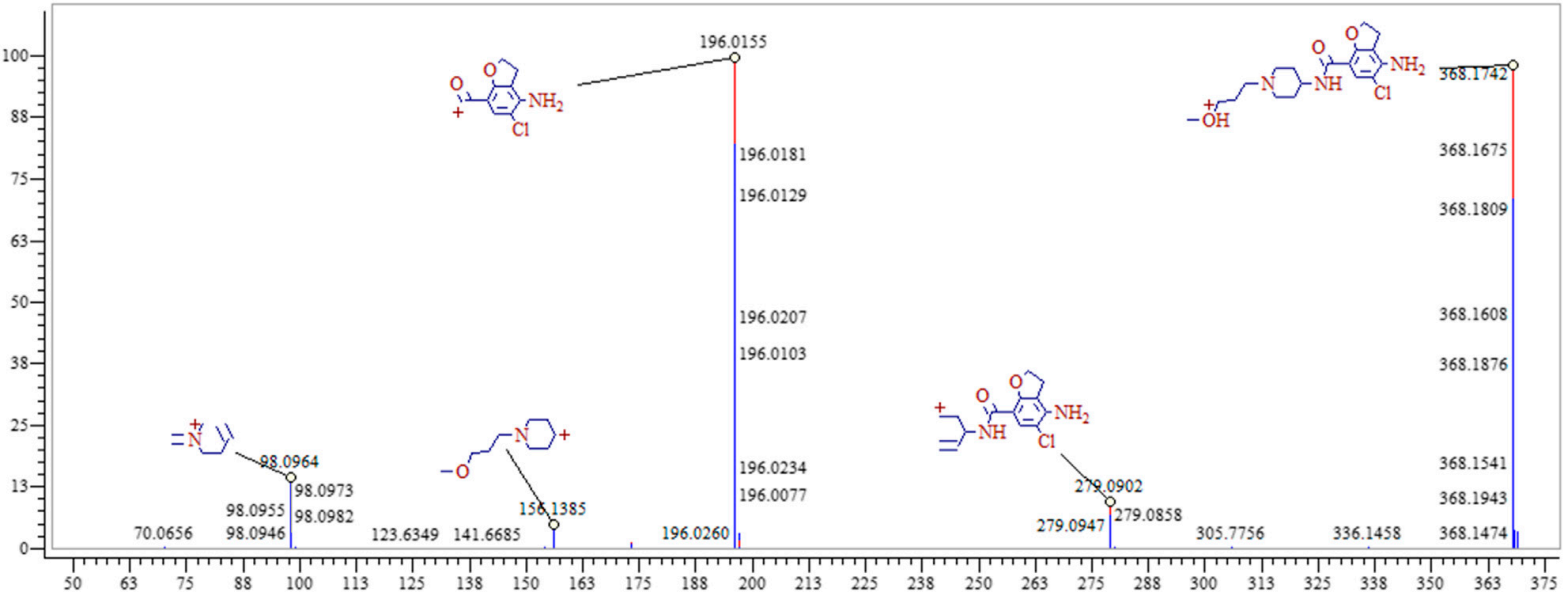
The MS^2^ spectrum and corresponding fragment ions of prucalopride.

The entire process optimization strategy was applied for comprehensive in vivo metabolite profiling of prucalopride in rats, and 47 tentative metabolites were identified in total. The MS2 spectrum and corresponding fragment ions of prucalopride in Figure 6. By analyzing metabolite structures, they mainly occurred in phase I metabolic reaction including oxidation reaction, dehydration, desaturation, and glucuronide conjugation, the typical phase Ⅱ metabolite reaction. But beyond that we also found some interesting phenomena that the drug could be metabolized by combining with the ornithine and glutamine, and some important endogenous amino acids. Besides, the palmitoyl conjugation was also discovered in the urine and feces. As shown in Figure 2, the structural formula of prucalopride was C_18_H_26_ClN_3_O_3_, with MS spectrum including the characteristic product ions of the *m/z* 279.09, *m/z* 196.02, *m/z* 173.16, *m/z* 156.12, *m/z* 98.10, and *m/z* 70.07. And based on the drug and metabolites’ structural characteristics, the changes mainly contained: oxidation or desaturation of piperidine ring or furan ring in diffident positions, demethylation of alkyl chain moiety in piperidine ring, separation of alkyl side chain, amide bond breaking, opening of piperidine ring or furan ring and conjugation reaction combined with the above.

## Conclusion

In this study, an entire process optimization strategy was proposed for comprehensive in vivo metabolite profiling of prucalopride in rat plasma, urine, and feces samples after oral administration based on ultra-performance liquid chromatography with Q-Exactive hybrid quadrupole–orbitrap high-resolution mass spectrometry. The strategy was composed of sample collection, optimization sample preparation, and chromatographic and mass spectrometry conditions, establishment of MDF and BS model in CD, and structural annotation and metabolites identification. The proposed strategy was applied into the prucalopride and 47 metabolites were identified in total, among which 41 metabolites were not previously reported. Compared with other current methods, this strategy is time saving and easily processable. And this method not only can solve the crucial problem obstructing in the data processing and identification of metabolites more efficiently, but also provide convenience for confirmation of the structural reliability. Especially for identification of complex components’ metabolites, for example, natural medicine, this method could extract metabolite-related dataset based on the components one by one and obtain respective related data, and this is a great breakthrough in the field of the metabolite identification.

## Data Availability

The original contributions presented in the study are included in the article/Supplementary Material, further inquiries can be directed to the corresponding authors.

## Abbreviations:

AIF, all-ion fragmentation; BS, background subtraction; CD, compound discovery; DD, direct dilution; HESI, heated electrospray ionization; HRMS, high-resolution mass spectrometry; IDA, information-dependent acquisition; LLE, liquid–liquid extraction; MDF, mass defect filter; PP, protein precipitation; SPE, solid phase extraction; SRM, selected reaction monitoring; UHPLC, ultra-performance liquid chromatography.
