# Supplementary material for: An Entire Process Optimization Strategy for Comprehensive In Vivo Metabolite Profiling of Prucalopride in Rats Based on Ultra-Performance Liquid Chromatography With Q-Exactive Hybrid Quadrupole–Orbitrap High-Resolution Mass Spectrometry
Source: Front Pharmacol. 2021 May 7;12:610226. doi: 10.3389/fphar.2021.610226 (PMC8138455; doi:10.3389/fphar.2021.610226)
Supplement: Supplementary file 1 [file DataSheet1.PDF]

Figure S1 The extraction flow chromatographys and MS/MS spectrum of prucalopride and metabolites.

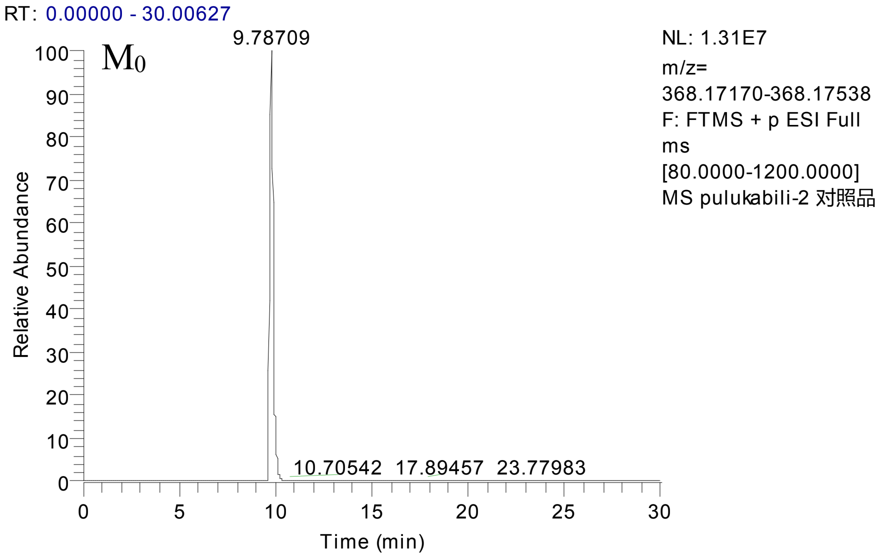

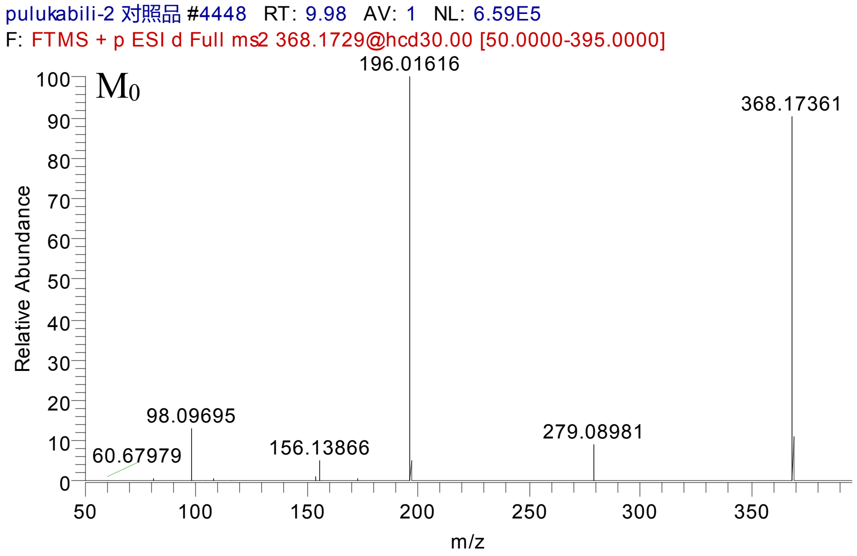

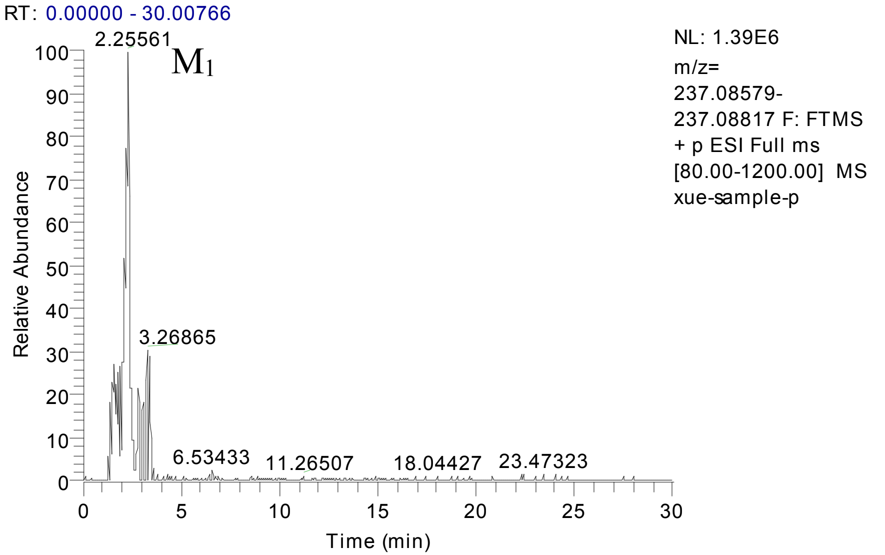

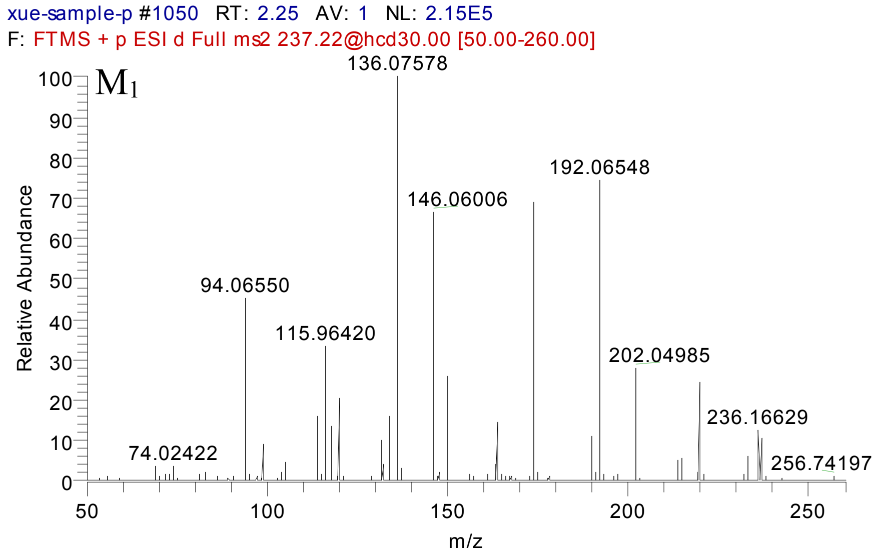

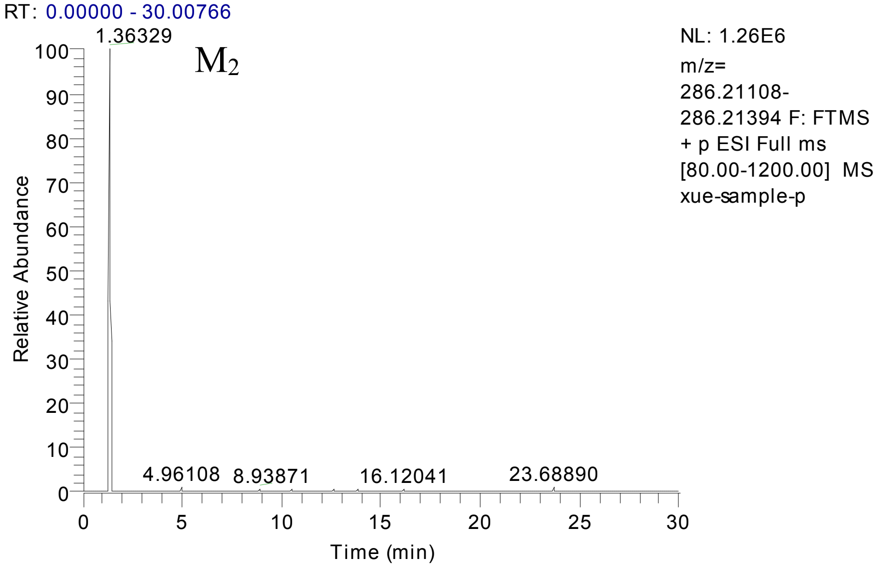
RT: 0.00000 - 30.00766
M2
1.36329
4.96108
8.93871
16.12041
23.68890
NL: 1.26E6
m/z= 286.21108-286.21394 F: FTMS + p ESI Full ms [80.00-1200.00] MS xue-sample-p
Relative Abundance
Time (min)

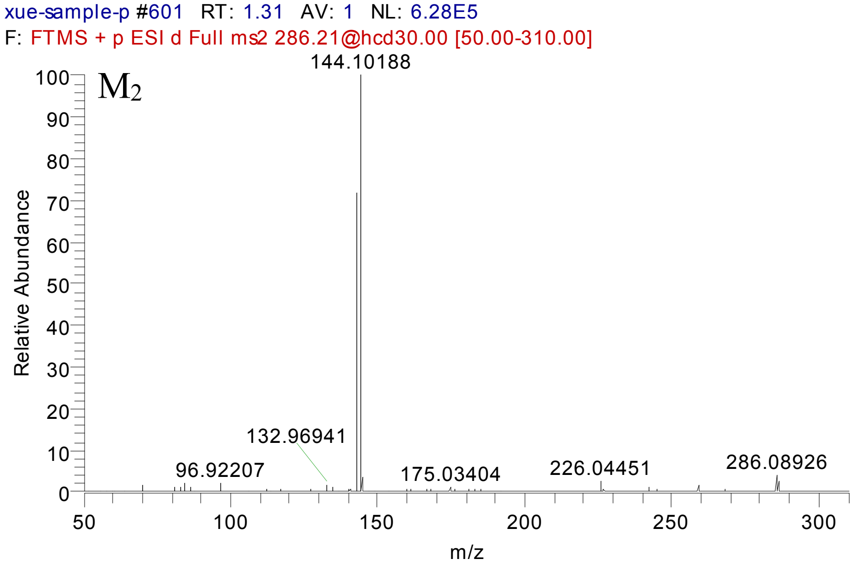
xue-sample-p #601 RT: 1.31 AV: 1 NL: 6.28E5
F: FTMS + p ESI d Full ms2 286.21@hcd30.00 [50.00-310.00]
M2
144.10188
132.96941
96.92207
175.03404
226.04451
286.08926
Relative Abundance
m/z

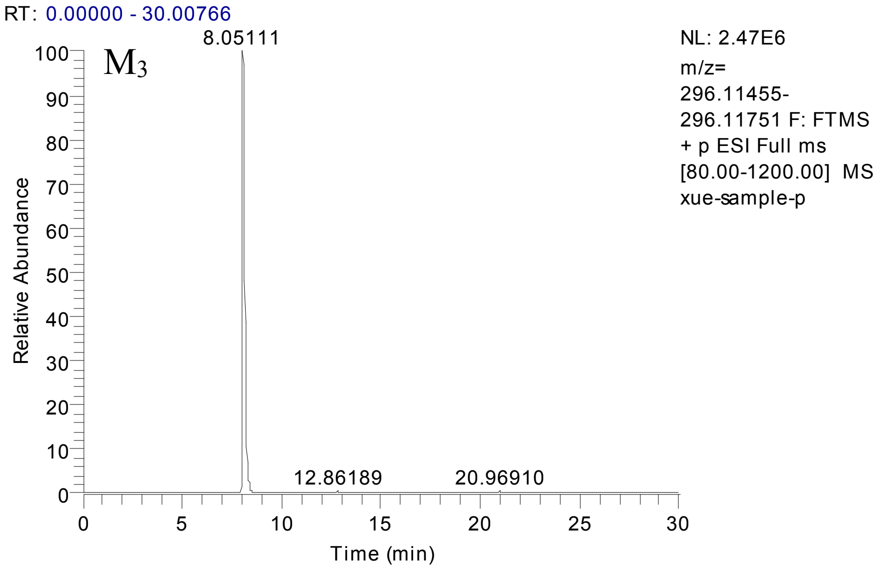
RT: 0.00000 - 30.00766
M3
8.05111
12.86189
20.96910
NL: 2.47E6
m/z= 296.11455-296.11751 F: FTMS + p ESI Full ms [80.00-1200.00] MS xue-sample-p
Relative Abundance
Time (min)

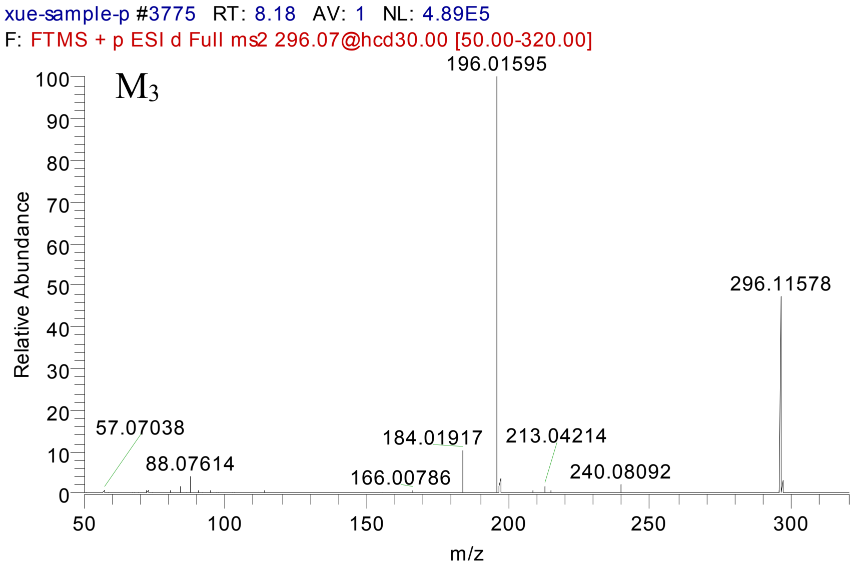
xue-sample-p #3775 RT: 8.18 AV: 1 NL: 4.89E5
F: FTMS + p ESI d Full ms2 296.07@hcd30.00 [50.00-320.00]
M3
196.01595
296.11578
57.07038
88.07614
184.01917
213.04214
166.00786
240.08092
Relative Abundance
m/z

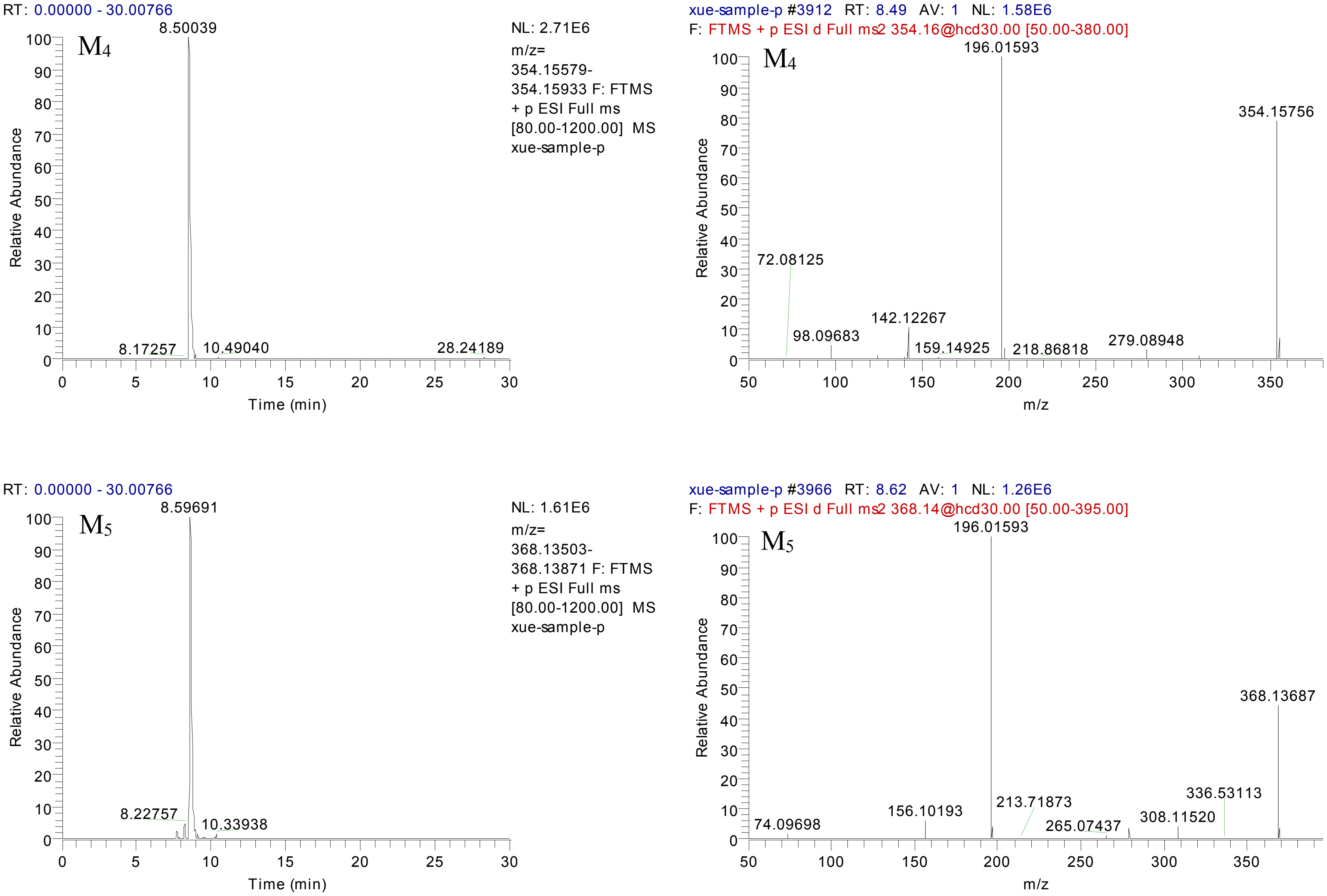
RT: 0.00000 - 30.00766
M4
8.50039
NL: 2.71E6
m/z= 354.15579-354.15933 F: FTMS + p ESI Full ms [80.00-1200.00] MS xue-sample-p
8.17257
10.49040
28.24189
Relative Abundance
Time (min)
xue-sample-p #3912 RT: 8.49 AV: 1 NL: 1.58E6
F: FTMS + p ESI d Full ms2 354.16@hcd30.00 [50.00-380.00]
M4
196.01593
354.15756
72.08125
98.09683
142.12267
159.14925
218.86818
279.08948
Relative Abundance
m/z
RT: 0.00000 - 30.00766
M5
8.59691
NL: 1.61E6
m/z= 368.13503-368.13871 F: FTMS + p ESI Full ms [80.00-1200.00] MS xue-sample-p
8.22757
10.33938
Relative Abundance
Time (min)
xue-sample-p #3966 RT: 8.62 AV: 1 NL: 1.26E6
F: FTMS + p ESI d Full ms2 368.14@hcd30.00 [50.00-395.00]
M5
196.01593
368.13687
336.53113
213.71873
156.10193
308.11520
74.09698
265.07437
Relative Abundance
m/z

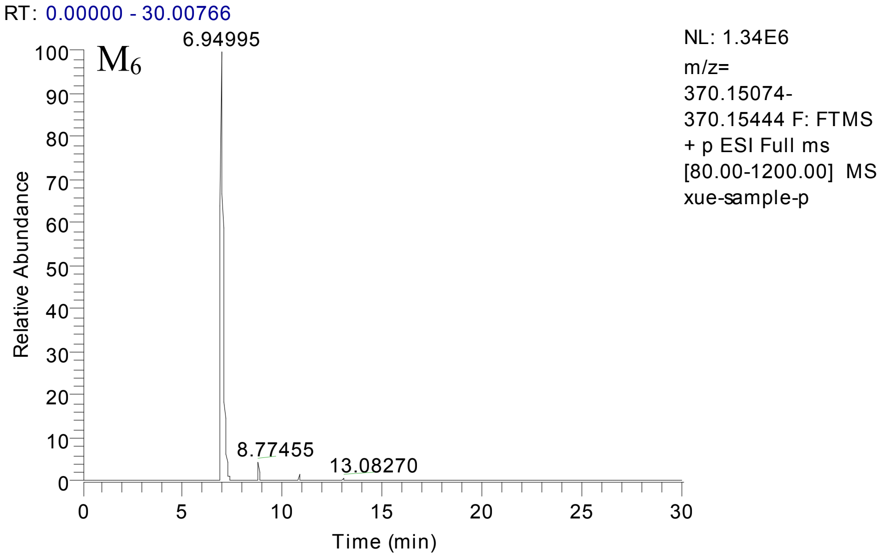
RT: 0.00000 - 30.00766
M6
6.94995
NL: 1.34E6
m/z=
370.15074-
370.15444 F: FTMS
+ p ESI Full ms
[80.00-1200.00] MS
xue-sample-p
8.77455
13.08270
Relative Abundance
Time (min)

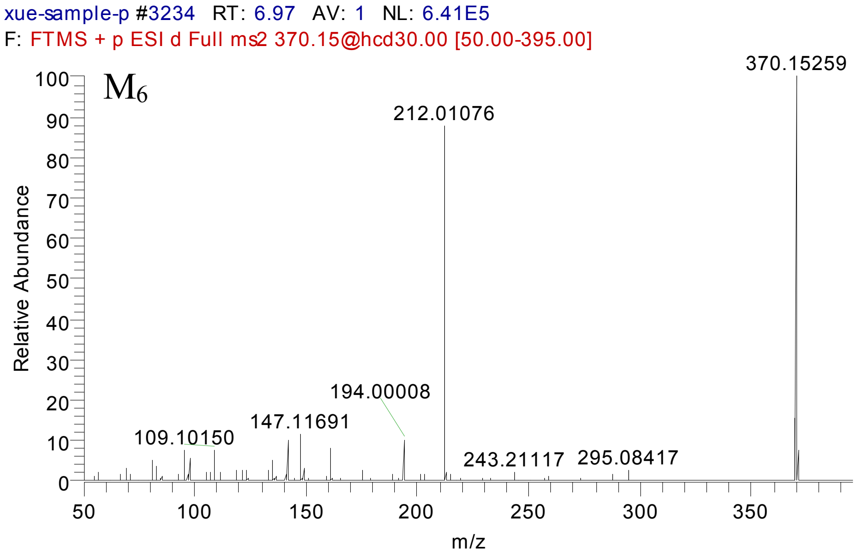
xue-sample-p #3234 RT: 6.97 AV: 1 NL: 6.41E5
F: FTMS + p ESI d Full ms2 370.15@hcd30.00 [50.00-395.00]
M6
370.15259
212.01076
194.00008
147.11691
109.10150
243.21117
295.08417
Relative Abundance
m/z

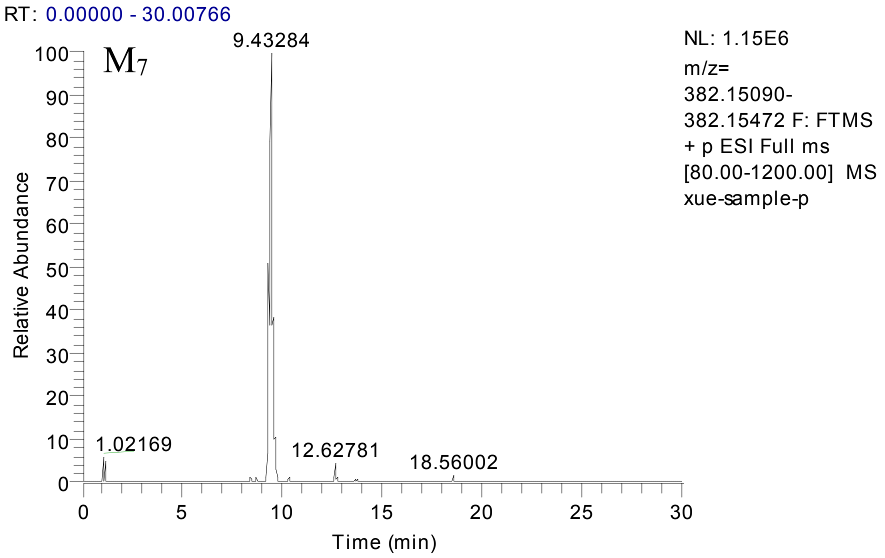
RT: 0.00000 - 30.00766
M7
9.43284
NL: 1.15E6
m/z=
382.15090-
382.15472 F: FTMS
+ p ESI Full ms
[80.00-1200.00] MS
xue-sample-p
1.02169
12.62781
18.56002
Relative Abundance
Time (min)

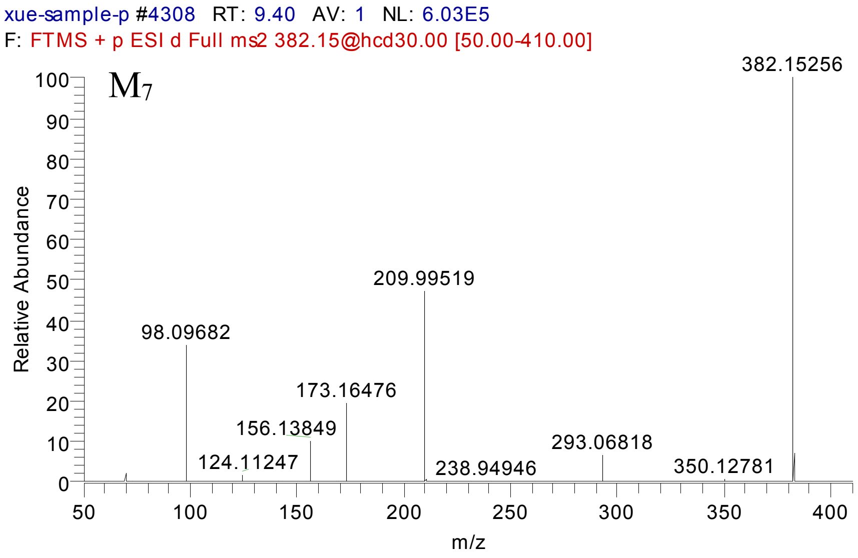
xue-sample-p #4308 RT: 9.40 AV: 1 NL: 6.03E5
F: FTMS + p ESI d Full ms2 382.15@hcd30.00 [50.00-410.00]
M7
382.15256
209.99519
98.09682
173.16476
156.13849
124.11247
238.94946
293.06818
350.12781
Relative Abundance
m/z

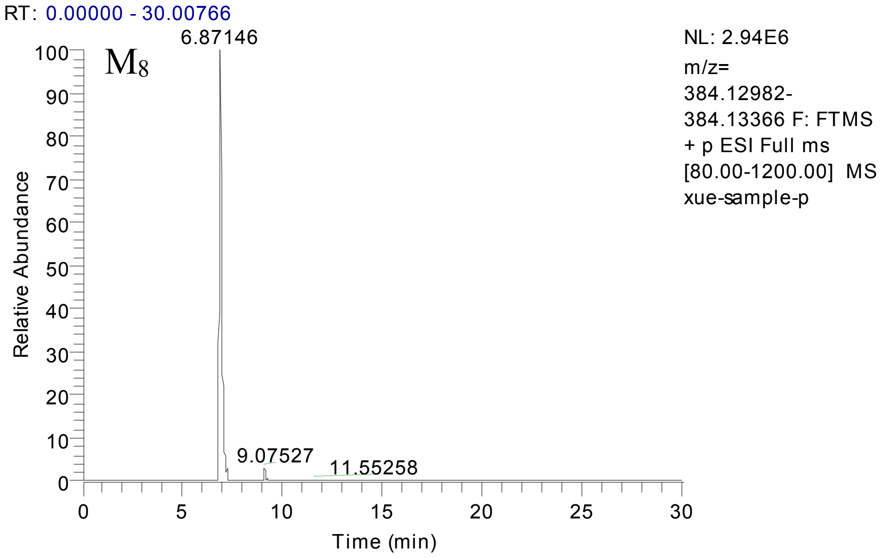
RT: 0.00000 - 30.00766
M8
6.87146
NL: 2.94E6
m/z=
384.12982-
384.13366 F: FTMS
+ p ESI Full ms
[80.00-1200.00] MS
xue-sample-p
9.07527
11.55258
Relative Abundance
Time (min)

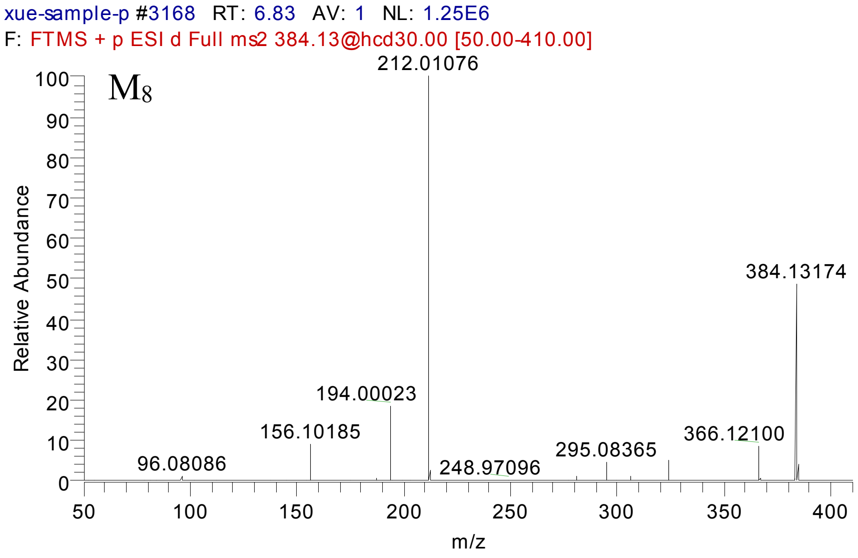
xue-sample-p #3168 RT: 6.83 AV: 1 NL: 1.25E6
F: FTMS + p ESI d Full ms2 384.13@hcd30.00 [50.00-410.00]
M8
212.01076
384.13174
194.00023
156.10185
366.12100
295.08365
96.08086
248.97096
Relative Abundance
m/z

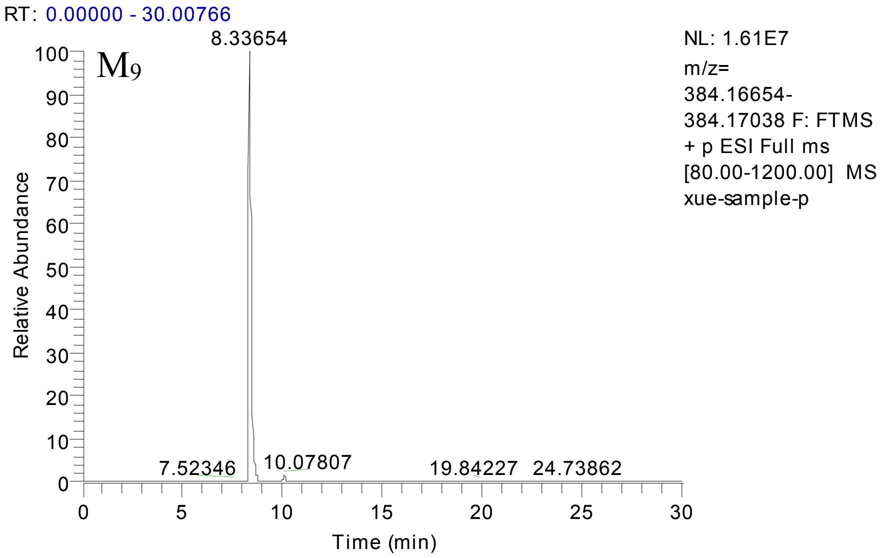
RT: 0.00000 - 30.00766
M9
8.33654
NL: 1.61E7
m/z=
384.16654-
384.17038 F: FTMS
+ p ESI Full ms
[80.00-1200.00] MS
xue-sample-p
7.52346
10.07807
19.84227
24.73862
Relative Abundance
Time (min)

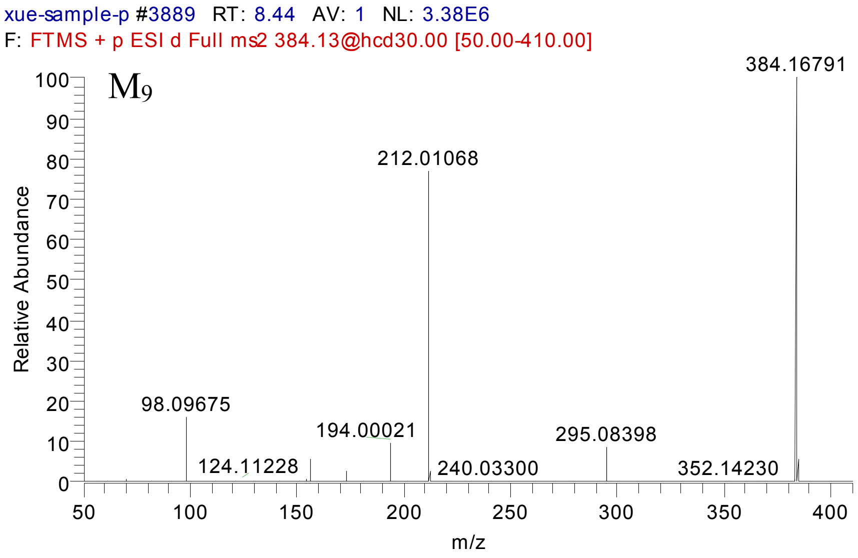
xue-sample-p #3889 RT: 8.44 AV: 1 NL: 3.38E6
F: FTMS + p ESI d Full ms2 384.13@hcd30.00 [50.00-410.00]
M9
384.16791
212.01068
98.09675
194.00021
295.08398
124.11228
240.03300
352.14230
Relative Abundance
m/z

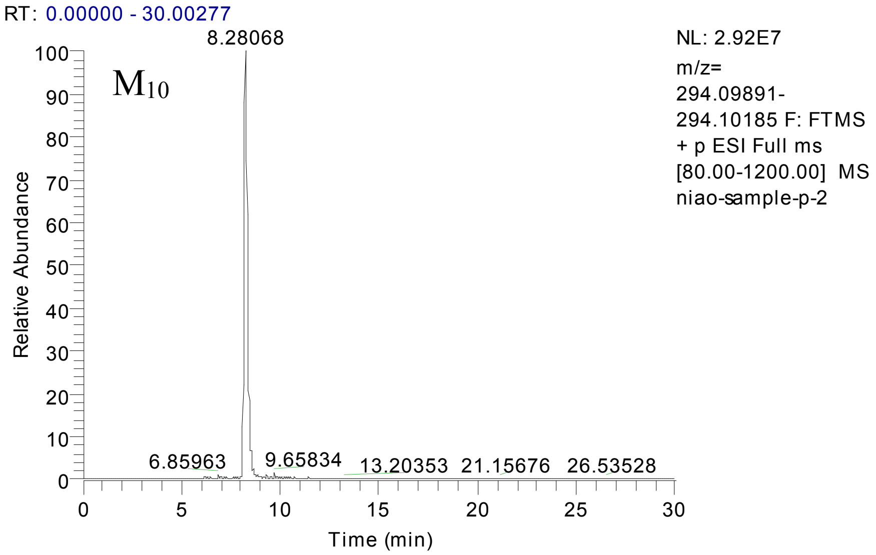
RT: 0.00000 - 30.00277
M10
8.28068
NL: 2.92E7
m/z=
294.09891-
294.10185 F: FTMS
+ p ESI Full ms
[80.00-1200.00] MS
niao-sample-p-2
6.85963
9.65834
13.20353
21.15676
26.53528
Relative Abundance
Time (min)

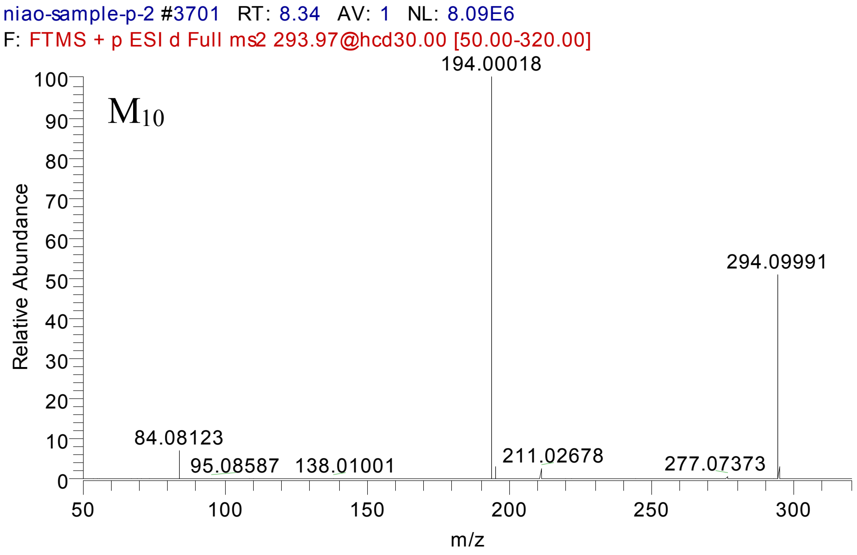
niao-sample-p-2 #3701 RT: 8.34 AV: 1 NL: 8.09E6
F: FTMS + p ESI d Full ms2 293.97@hcd30.00 [50.00-320.00]
M10
194.00018
294.09991
84.08123
95.08587
138.01001
211.02678
277.07373
Relative Abundance
m/z

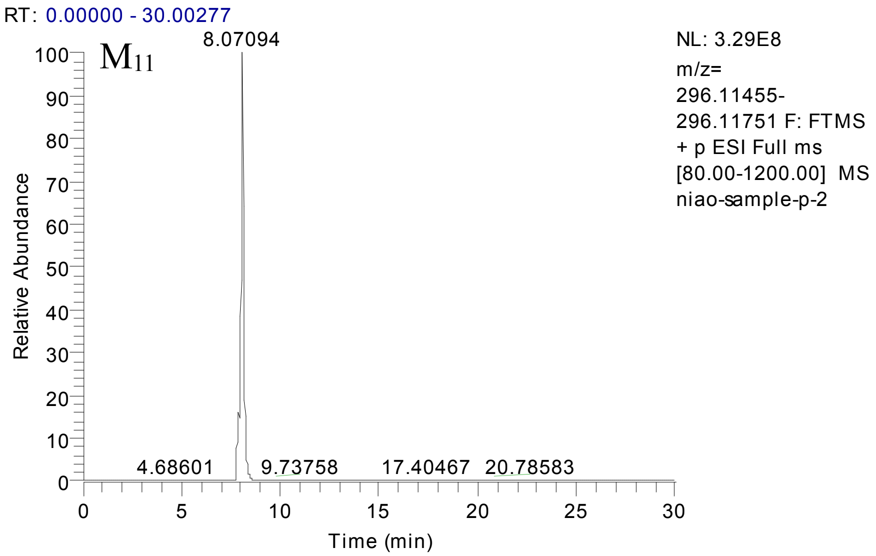
RT: 0.00000 - 30.00277
M11
8.07094
NL: 3.29E8
m/z=
296.11455-
296.11751 F: FTMS
+ p ESI Full ms
[80.00-1200.00] MS
niao-sample-p-2
4.68601
9.73758
17.40467
20.78583
Relative Abundance
Time (min)

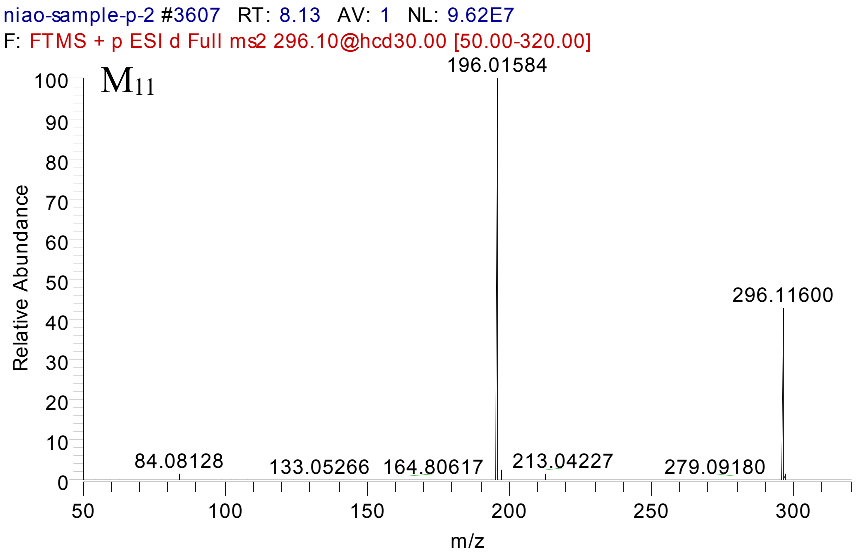
niao-sample-p-2 #3607 RT: 8.13 AV: 1 NL: 9.62E7
F: FTMS + p ESI d Full ms2 296.10@hcd30.00 [50.00-320.00]
M11
196.01584
296.11600
84.08128
133.05266
164.80617
213.04227
279.09180
Relative Abundance
m/z

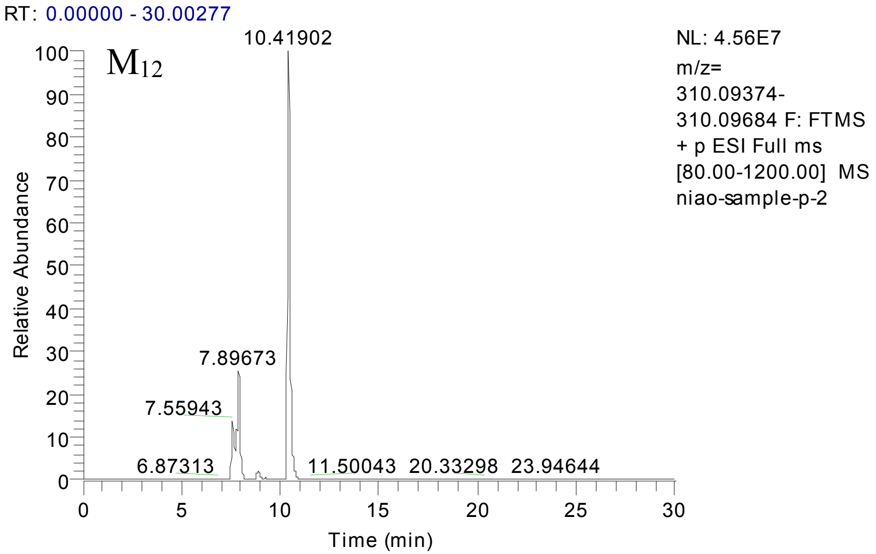
RT: 0.00000 - 30.00277
M12
10.41902
NL: 4.56E7
m/z=
310.09374-
310.09684 F: FTMS
+ p ESI Full ms
[80.00-1200.00] MS
niao-sample-p-2
7.89673
7.55943
6.87313
11.50043
20.33298
23.94644
Relative Abundance
Time (min)

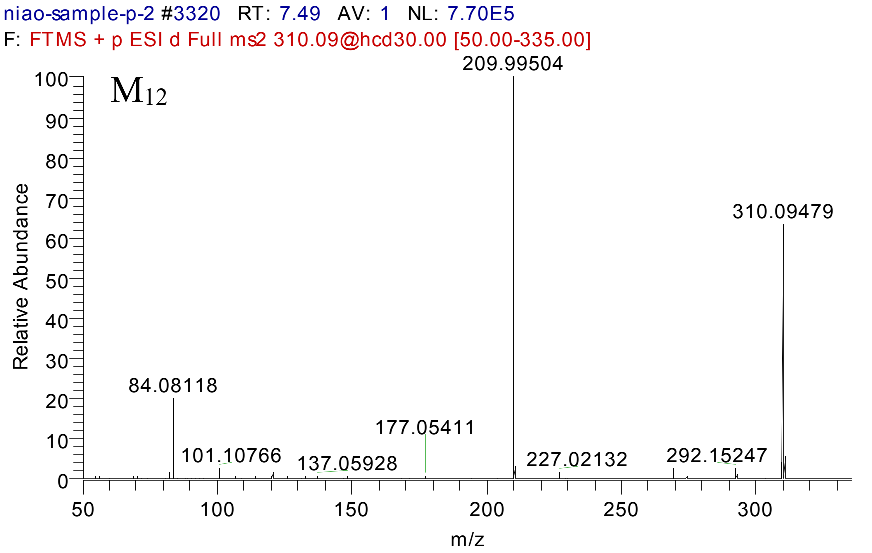
niao-sample-p-2 #3320 RT: 7.49 AV: 1 NL: 7.70E5
F: FTMS + p ESI d Full ms2 310.09@hcd30.00 [50.00-335.00]
M12
209.99504
310.09479
84.08118
177.05411
101.10766
137.05928
227.02132
292.15247
Relative Abundance
m/z

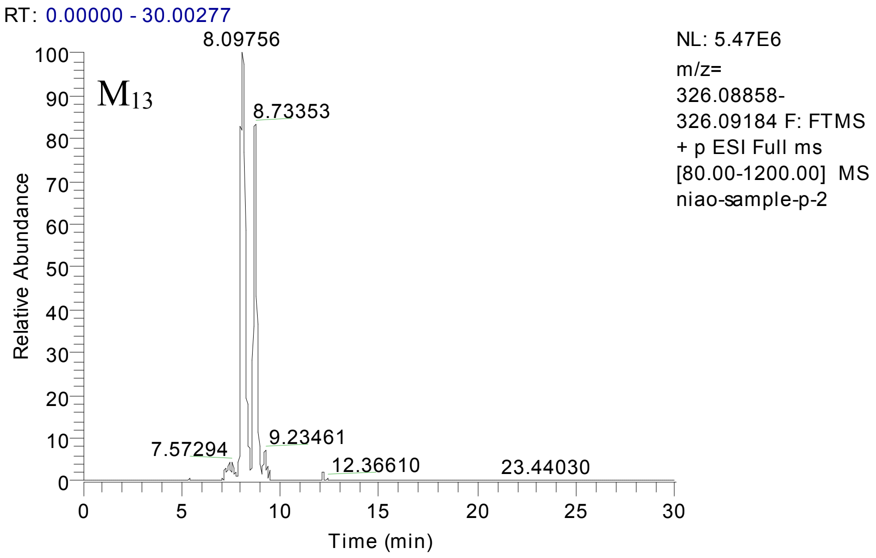
RT: 0.00000 - 30.00277
M13
8.09756
8.73353
NL: 5.47E6
m/z=
326.08858-
326.09184 F: FTMS
+ p ESI Full ms
[80.00-1200.00] MS
niao-sample-p-2
7.57294
9.23461
12.36610
23.44030
Relative Abundance
Time (min)

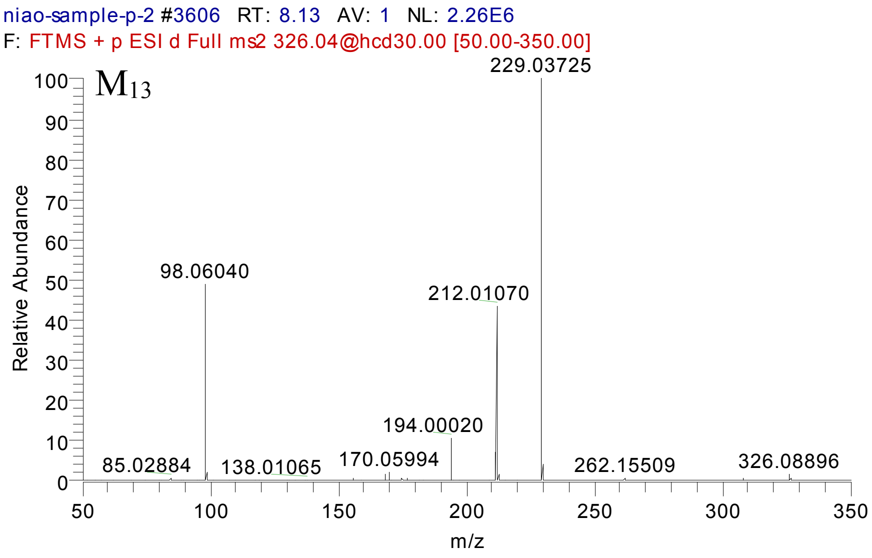
niao-sample-p-2 #3606 RT: 8.13 AV: 1 NL: 2.26E6
F: FTMS + p ESI d Full ms2 326.04@hcd30.00 [50.00-350.00]
M13
229.03725
98.06040
212.01070
194.00020
85.02884
138.01065
170.05994
262.15509
326.08896
Relative Abundance
m/z

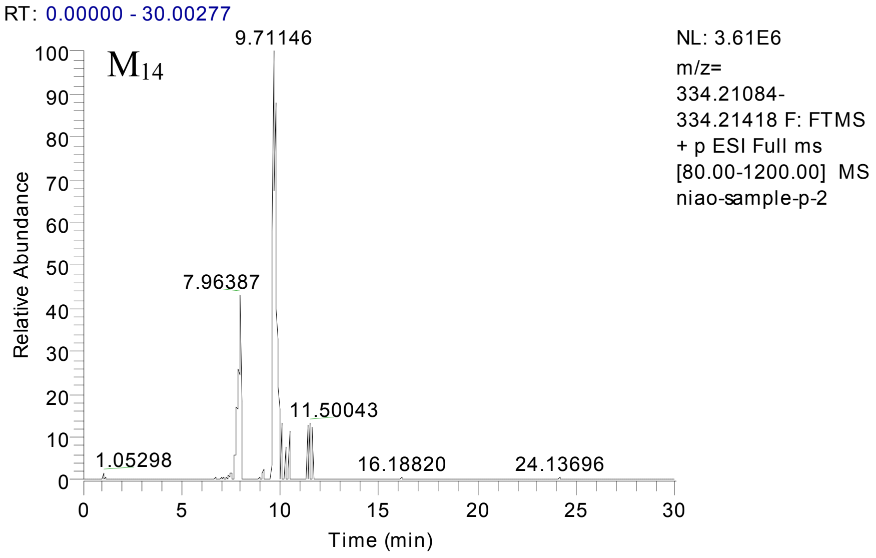
RT: 0.00000 - 30.00277
M14
NL: 3.61E6
m/z=
334.21084-
334.21418 F: FTMS
+ p ESI Full ms
[80.00-1200.00] MS
niao-sample-p-2
9.71146
7.96387
11.50043
1.05298
16.18820
24.13696
Relative Abundance
Time (min)
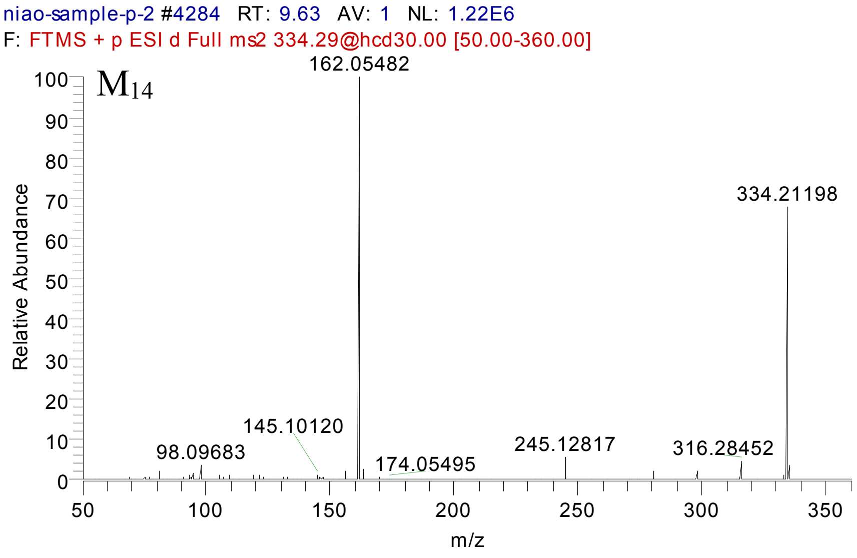
niao-sample-p-2 #4284 RT: 9.63 AV: 1 NL: 1.22E6
F: FTMS + p ESI d Full ms2 334.29@hcd30.00 [50.00-360.00]
M14
162.05482
334.21198
145.10120
98.09683
174.05495
245.12817
316.28452
Relative Abundance
m/z
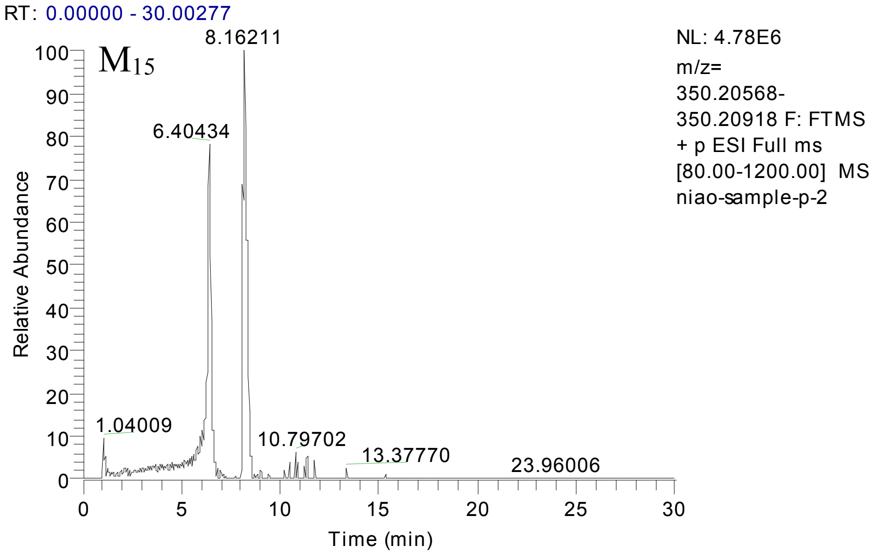
RT: 0.00000 - 30.00277
M15
NL: 4.78E6
m/z=
350.20568-
350.20918 F: FTMS
+ p ESI Full ms
[80.00-1200.00] MS
niao-sample-p-2
8.16211
6.40434
1.04009
10.79702
13.37770
23.96006
Relative Abundance
Time (min)
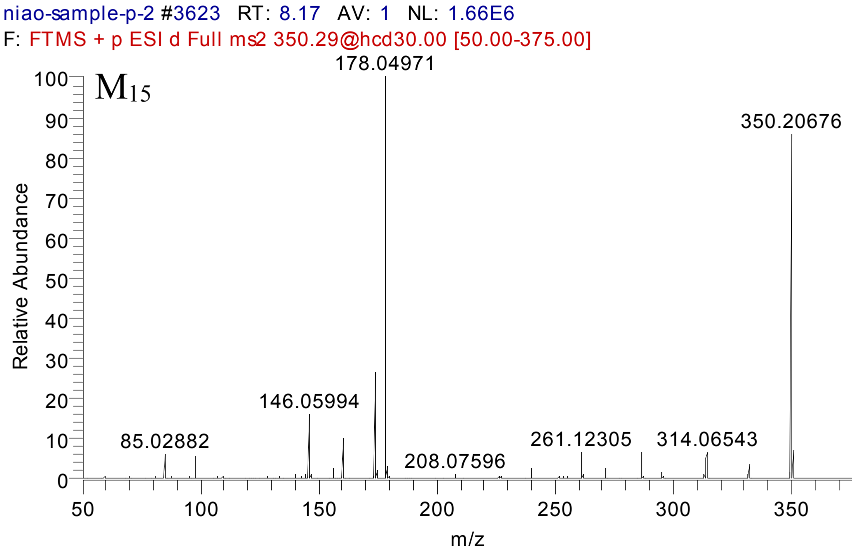
niao-sample-p-2 #3623 RT: 8.17 AV: 1 NL: 1.66E6
F: FTMS + p ESI d Full ms2 350.29@hcd30.00 [50.00-375.00]
M15
178.04971
350.20676
146.05994
85.02882
208.07596
261.12305
314.06543
Relative Abundance
m/z

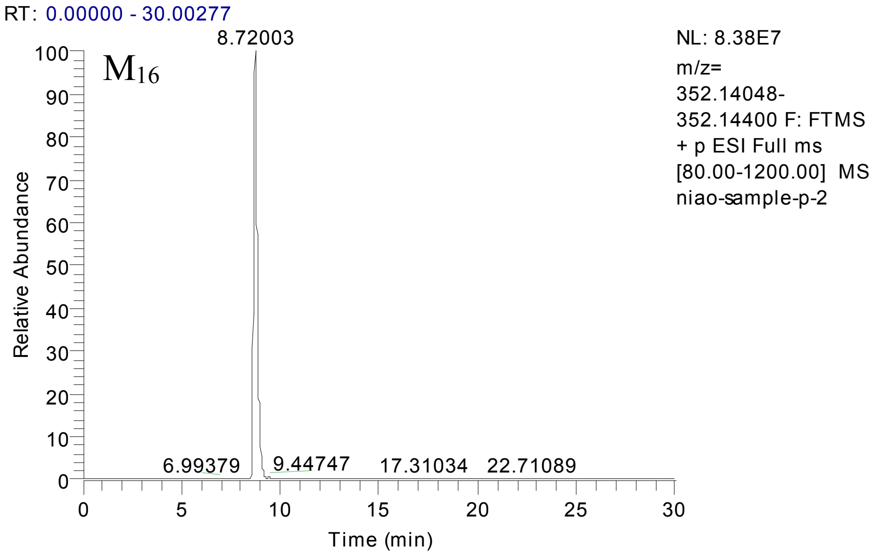
RT: 0.00000 - 30.00277
NL: 8.38E7
m/z= 352.14048-352.14400 F: FTMS + p ESI Full ms [80.00-1200.00] MS niao-sample-p-2
M16
8.72003
6.99379
9.44747
17.31034
22.71089
Relative Abundance
Time (min)

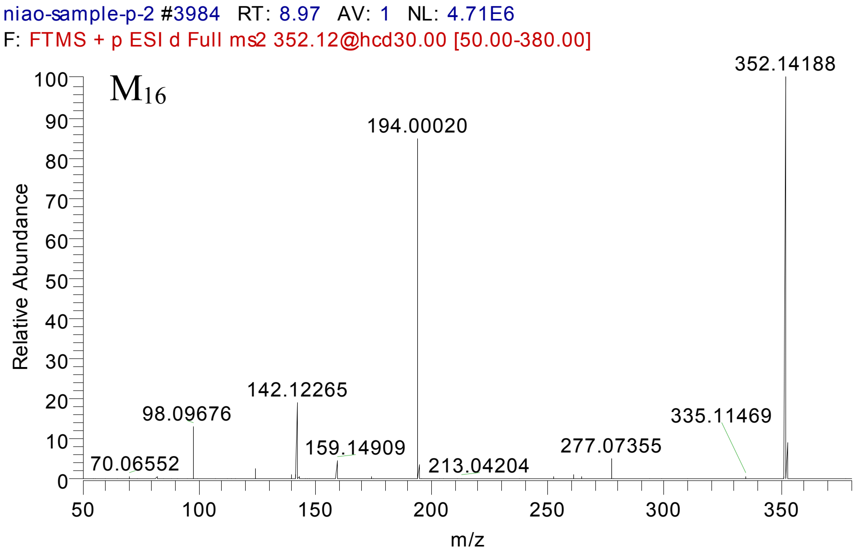
niao-sample-p-2 #3984 RT: 8.97 AV: 1 NL: 4.71E6
F: FTMS + p ESI d Full ms2 352.12@hcd30.00 [50.00-380.00]
M16
352.14188
194.00020
142.12265
98.09676
335.11469
277.07355
159.14909
70.06552
213.04204
Relative Abundance
m/z

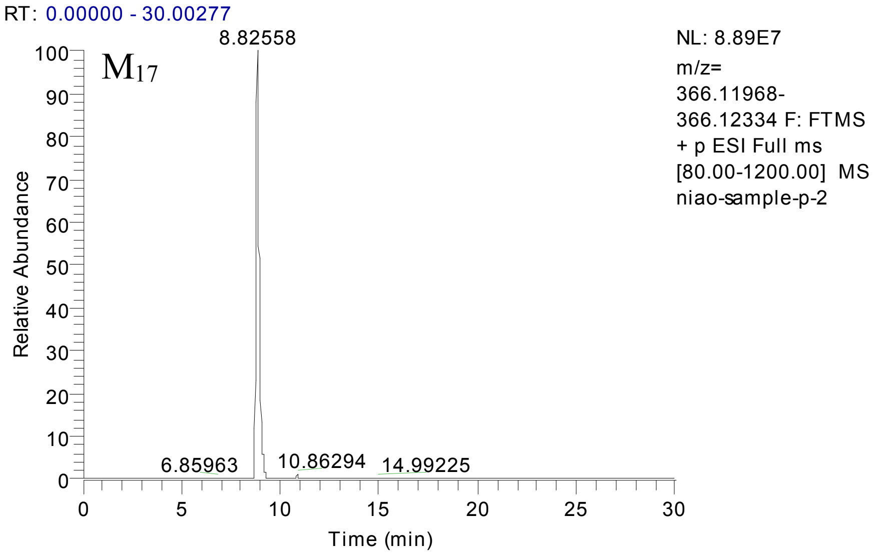
RT: 0.00000 - 30.00277
NL: 8.89E7
m/z= 366.11968-366.12334 F: FTMS + p ESI Full ms [80.00-1200.00] MS niao-sample-p-2
M17
8.82558
6.85963
10.86294
14.99225
Relative Abundance
Time (min)

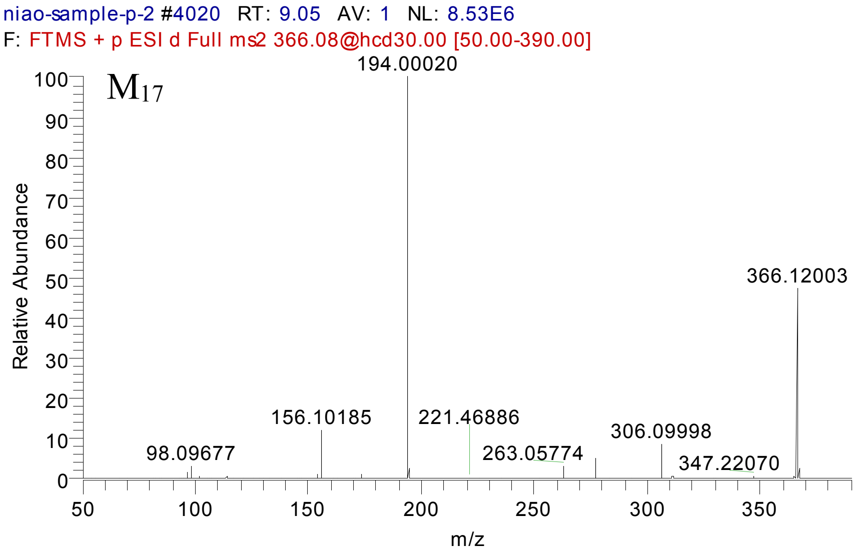
niao-sample-p-2 #4020 RT: 9.05 AV: 1 NL: 8.53E6
F: FTMS + p ESI d Full ms2 366.08@hcd30.00 [50.00-390.00]
M17
194.00020
366.12003
156.10185
221.46886
306.09998
98.09677
263.05774
347.22070
Relative Abundance
m/z

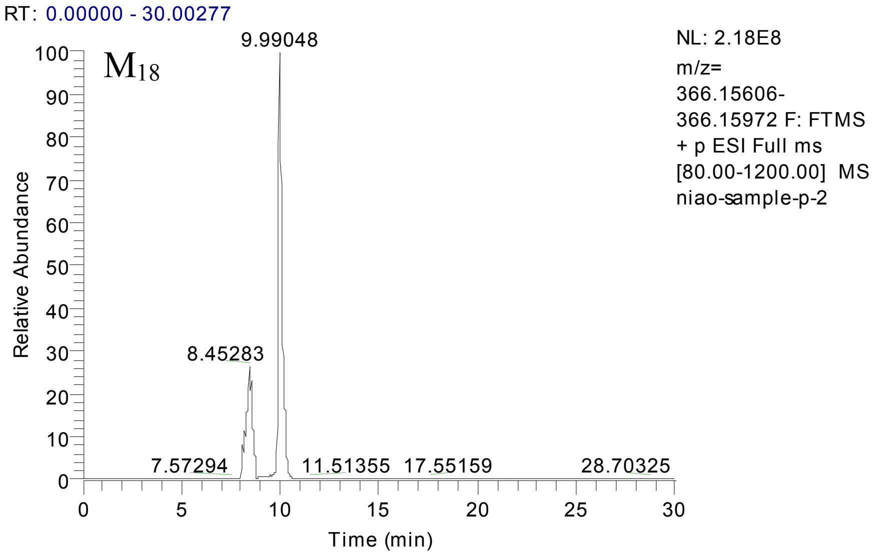
RT: 0.00000 - 30.00277
M18
9.99048
NL: 2.18E8
m/z=
366.15606-
366.15972 F: FTMS
+ p ESI Full ms
[80.00-1200.00] MS
niao-sample-p-2
8.45283
7.57294
11.51355
17.55159
28.70325
Relative Abundance
Time (min)

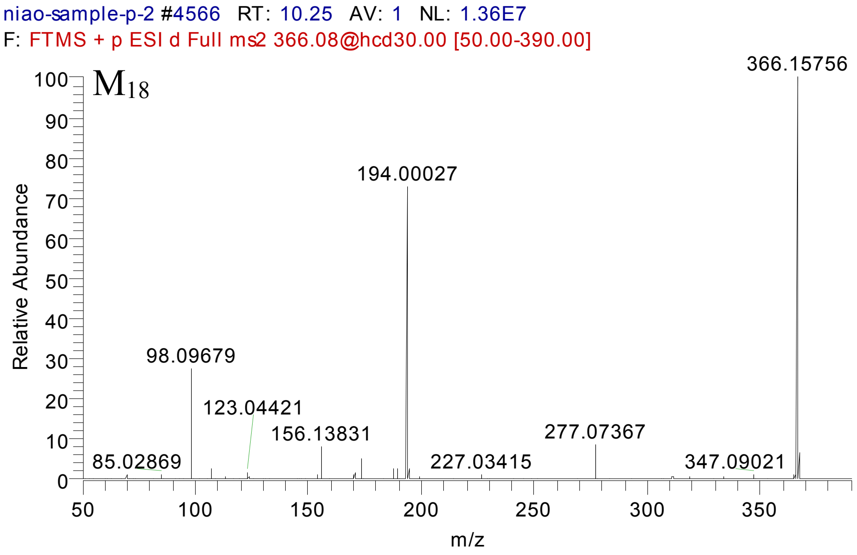
niao-sample-p-2 #4566 RT: 10.25 AV: 1 NL: 1.36E7
F: FTMS + p ESI d Full ms2 366.08@hcd30.00 [50.00-390.00]
M18
366.15756
194.00027
98.09679
123.04421
156.13831
277.07367
85.02869
227.03415
347.09021
Relative Abundance
m/z

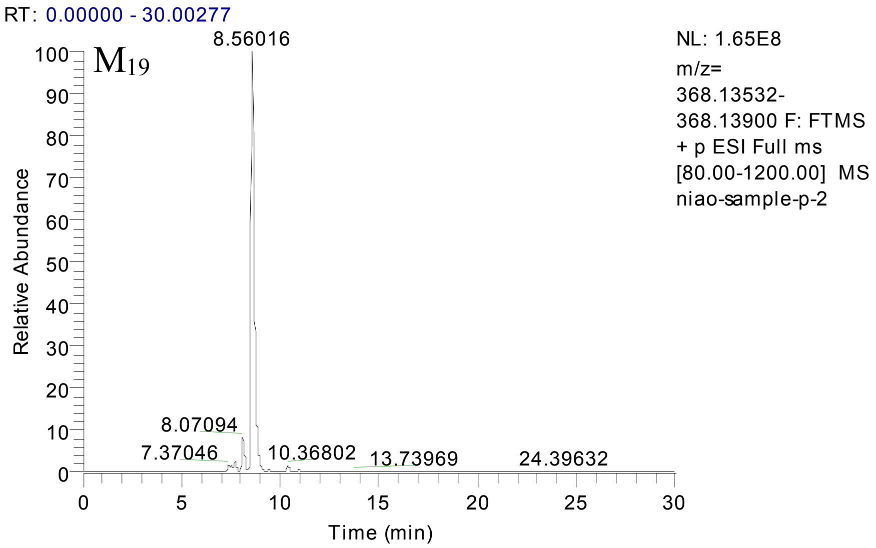
RT: 0.00000 - 30.00277
M19
8.56016
NL: 1.65E8
m/z=
368.13532-
368.13900 F: FTMS
+ p ESI Full ms
[80.00-1200.00] MS
niao-sample-p-2
8.07094
7.37046
10.36802
13.73969
24.39632
Relative Abundance
Time (min)

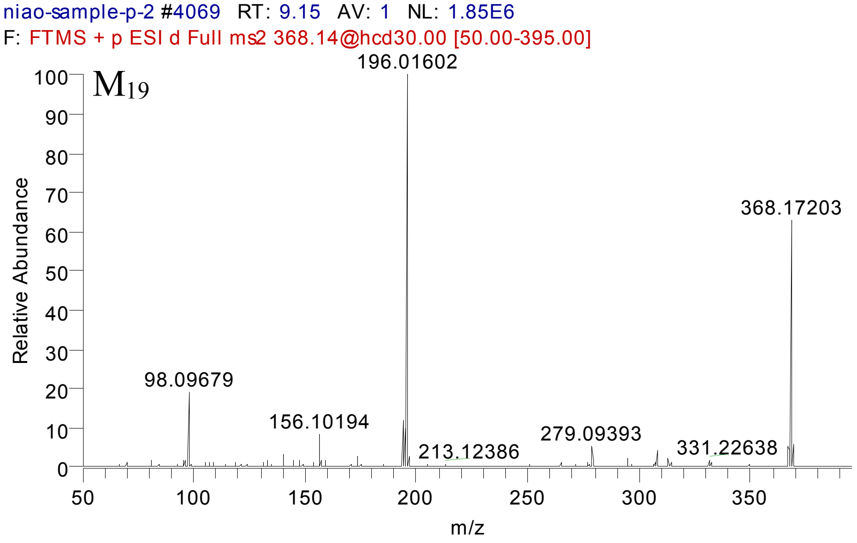
niao-sample-p-2 #4069 RT: 9.15 AV: 1 NL: 1.85E6
F: FTMS + p ESI d Full ms2 368.14@hcd30.00 [50.00-395.00]
M19
196.01602
368.17203
98.09679
156.10194
213.12386
279.09393
331.22638
Relative Abundance
m/z

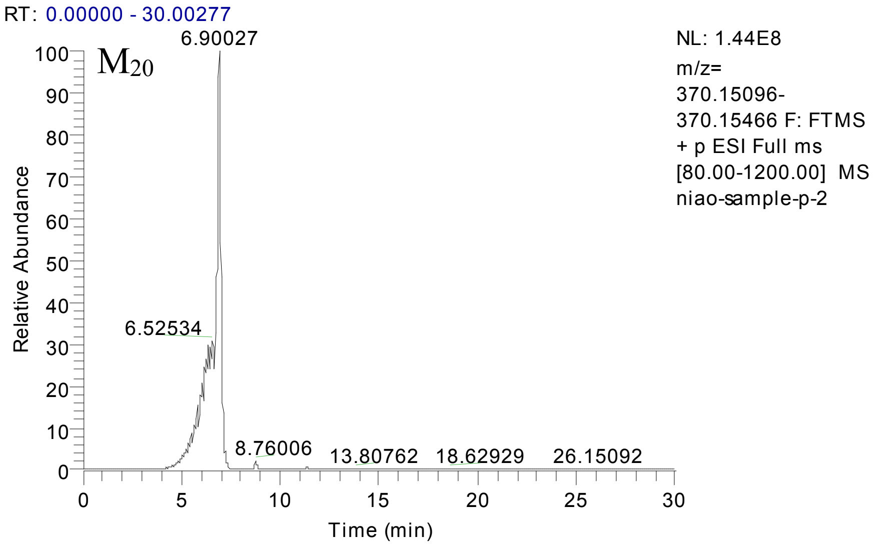
RT: 0.00000 - 30.00277
M20
6.90027
6.52534
8.76006
13.80762
18.62929
26.15092
NL: 1.44E8
m/z=
370.15096-
370.15466 F: FTMS
+ p ESI Full ms
[80.00-1200.00] MS
niao-sample-p-2
Relative Abundance
Time (min)

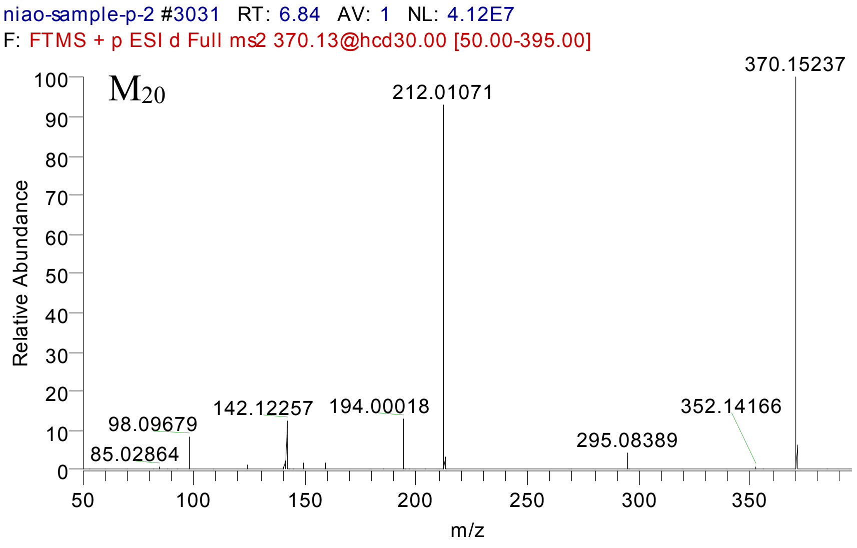
niao-sample-p-2 #3031 RT: 6.84 AV: 1 NL: 4.12E7
F: FTMS + p ESI d Full ms2 370.13@hcd30.00 [50.00-395.00]
M20
370.15237
212.01071
98.09679
142.12257
194.00018
352.14166
85.02864
295.08389
Relative Abundance
m/z

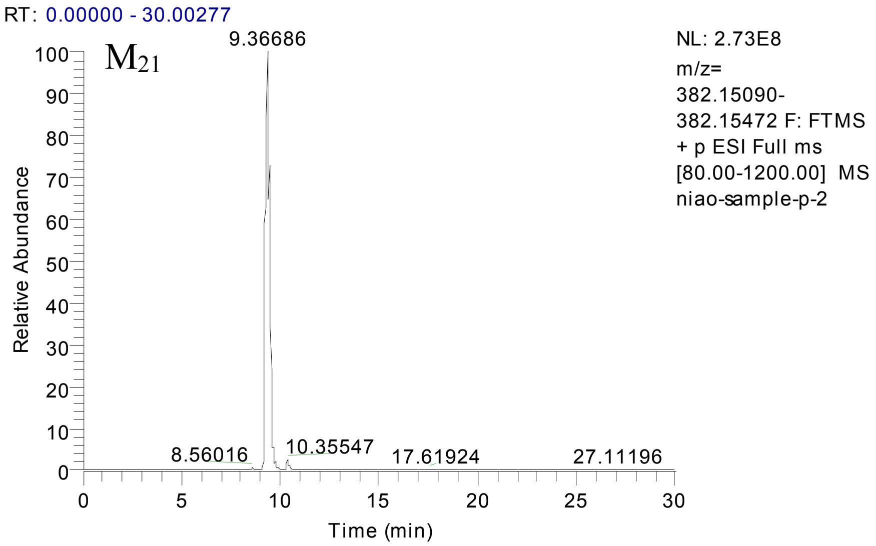
RT: 0.00000 - 30.00277
M21
9.36686
8.56016
10.35547
17.61924
27.11196
NL: 2.73E8
m/z=
382.15090-
382.15472 F: FTMS
+ p ESI Full ms
[80.00-1200.00] MS
niao-sample-p-2
Relative Abundance
Time (min)

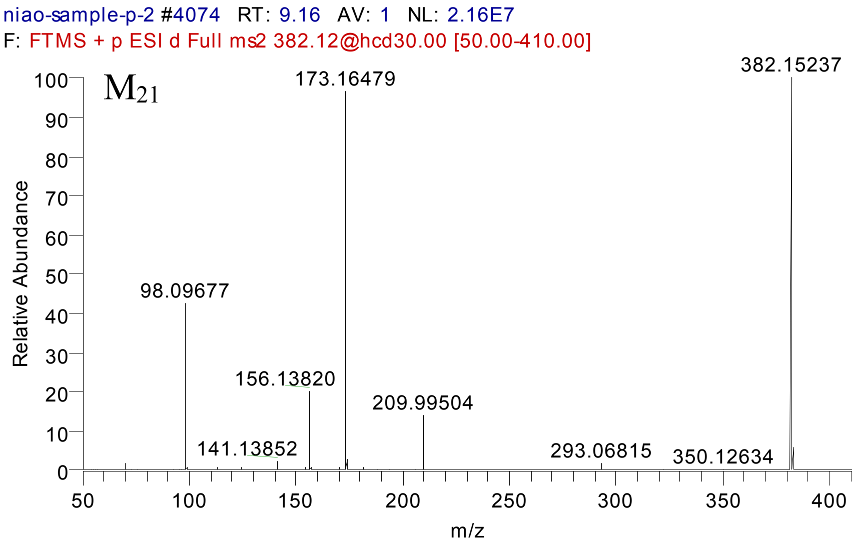
niao-sample-p-2 #4074 RT: 9.16 AV: 1 NL: 2.16E7
F: FTMS + p ESI d Full ms2 382.12@hcd30.00 [50.00-410.00]
M21
382.15237
173.16479
98.09677
156.13820
209.99504
141.13852
293.06815
350.12634
Relative Abundance
m/z

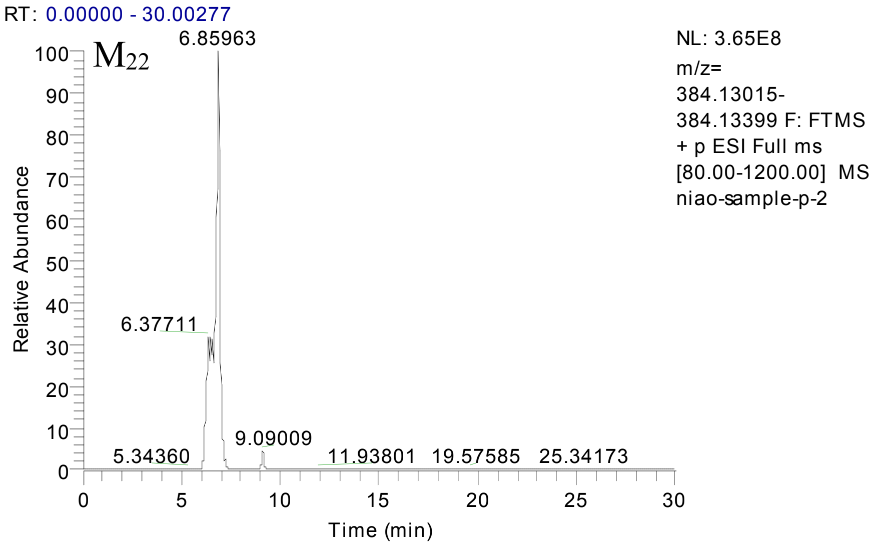
RT: 0.00000 - 30.00277
M22
6.85963
NL: 3.65E8
m/z=
384.13015-
384.13399 F: FTMS
+ p ESI Full ms
[80.00-1200.00] MS
niao-sample-p-2
6.37711
5.34360
9.09009
11.93801
19.57585
25.34173
Relative Abundance
Time (min)

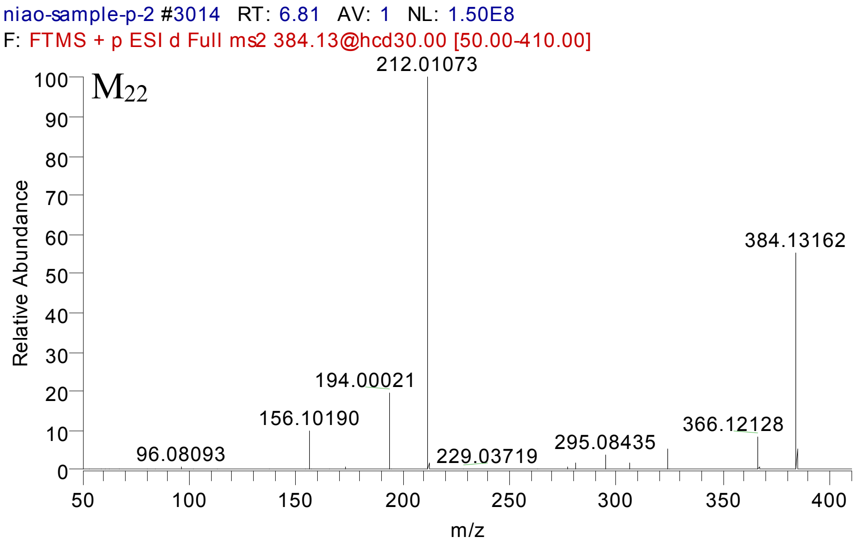
niao-sample-p-2 #3014 RT: 6.81 AV: 1 NL: 1.50E8
F: FTMS + p ESI d Full ms2 384.13@hcd30.00 [50.00-410.00]
M22
212.01073
384.13162
194.00021
156.10190
96.08093
229.03719
295.08435
366.12128
Relative Abundance
m/z

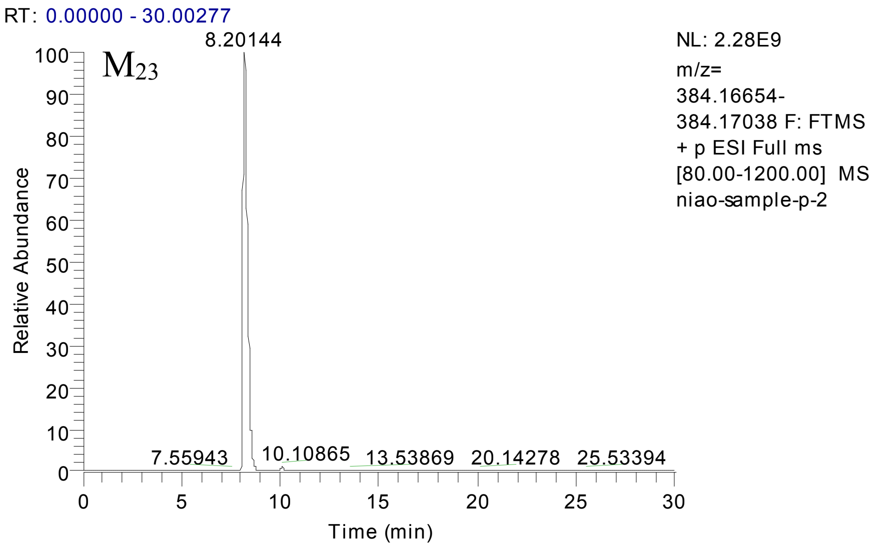
RT: 0.00000 - 30.00277
M23
8.20144
NL: 2.28E9
m/z=
384.16654-
384.17038 F: FTMS
+ p ESI Full ms
[80.00-1200.00] MS
niao-sample-p-2
7.55943
10.10865
13.53869
20.14278
25.53394
Relative Abundance
Time (min)

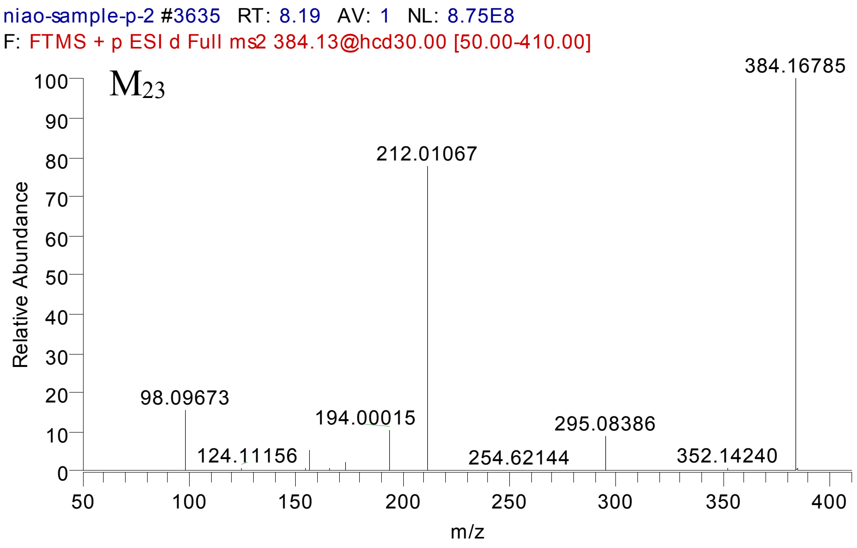
niao-sample-p-2 #3635 RT: 8.19 AV: 1 NL: 8.75E8
F: FTMS + p ESI d Full ms2 384.13@hcd30.00 [50.00-410.00]
M23
384.16785
212.01067
98.09673
124.11156
194.00015
254.62144
295.08386
352.14240
Relative Abundance
m/z

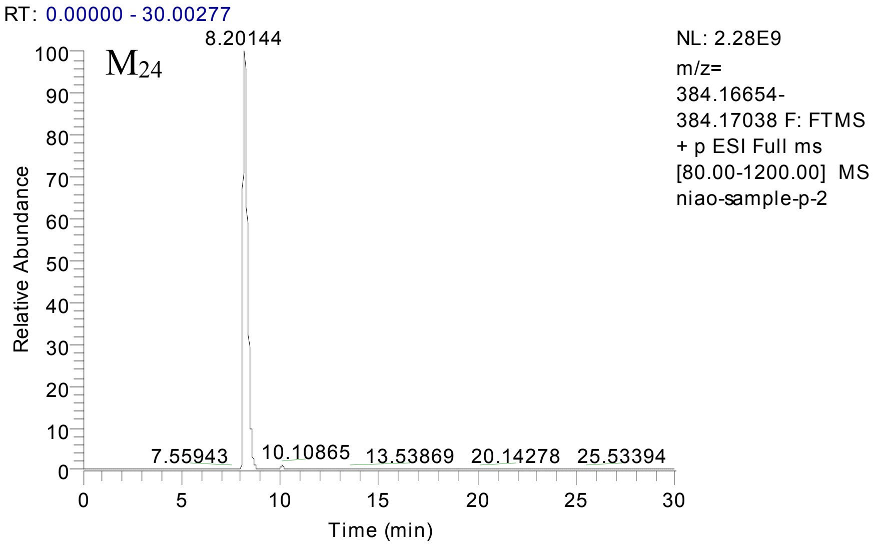
RT: 0.00000 - 30.00277
M24
8.20144
NL: 2.28E9
m/z=
384.16654-
384.17038 F: FTMS
+ p ESI Full ms
[80.00-1200.00] MS
niao-sample-p-2
7.55943
10.10865
13.53869
20.14278
25.53394
Relative Abundance
Time (min)

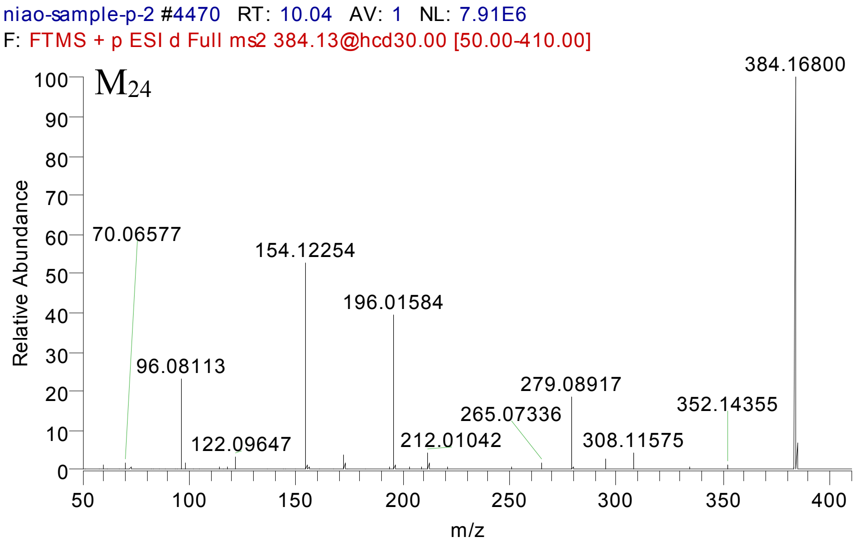
niao-sample-p-2 #4470 RT: 10.04 AV: 1 NL: 7.91E6
F: FTMS + p ESI d Full ms2 384.13@hcd30.00 [50.00-410.00]
M24
384.16800
70.06577
154.12254
196.01584
96.08113
279.08917
265.07336
352.14355
122.09647
212.01042
308.11575
Relative Abundance
m/z

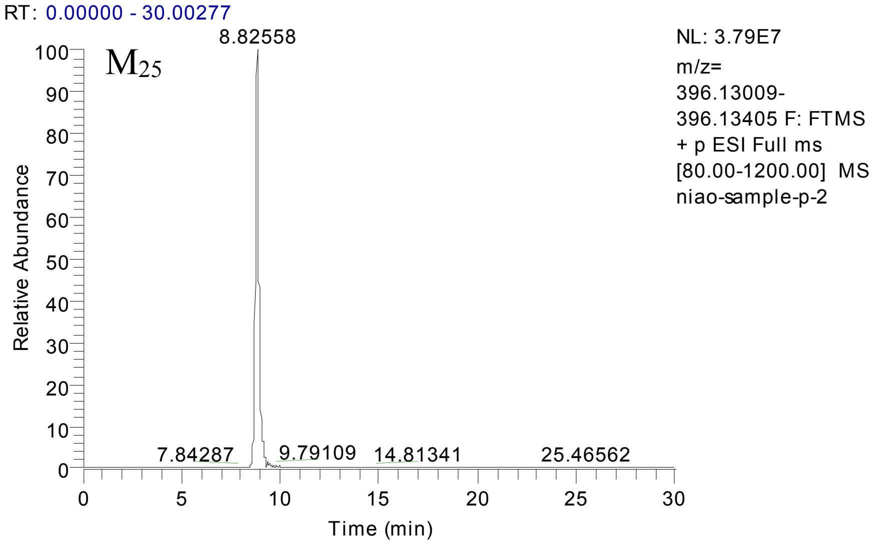
RT: 0.00000 - 30.00277
M25
8.82558
NL: 3.79E7
m/z=
396.13009-
396.13405 F: FTMS
+ p ESI Full ms
[80.00-1200.00] MS
niao-sample-p-2
7.84287
9.79109
14.81341
25.46562
Relative Abundance
Time (min)

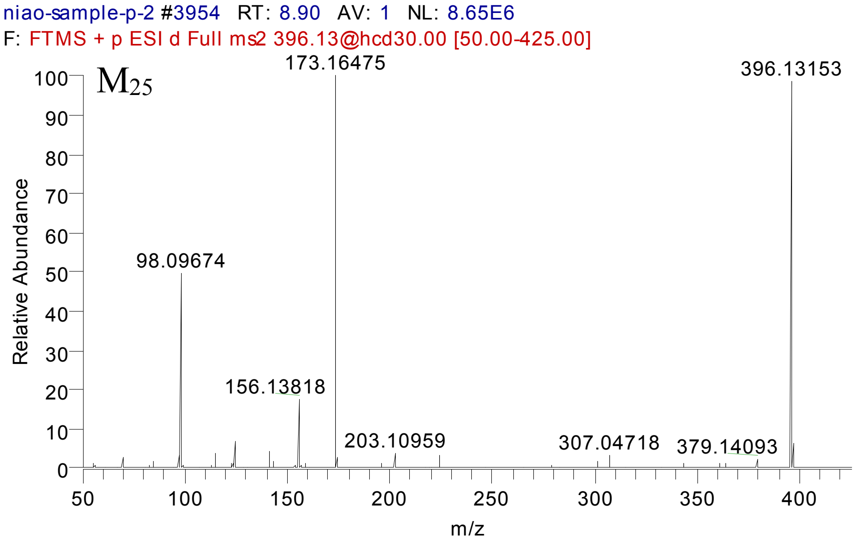
niao-sample-p-2 #3954 RT: 8.90 AV: 1 NL: 8.65E6
F: FTMS + p ESI d Full ms2 396.13@hcd30.00 [50.00-425.00]
M25
173.16475
396.13153
98.09674
156.13818
203.10959
307.04718
379.14093
Relative Abundance
m/z

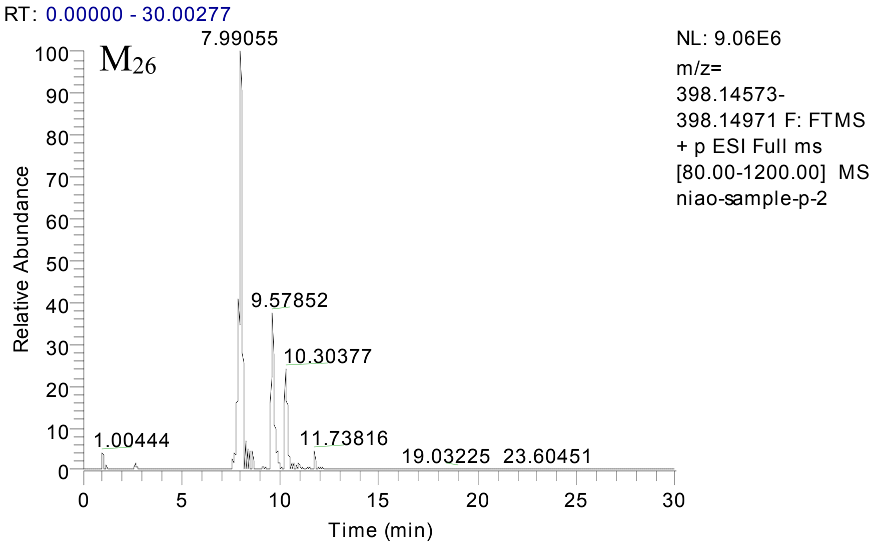
RT: 0.00000 - 30.00277
M26
7.99055
NL: 9.06E6
m/z=
398.14573-
398.14971 F: FTMS
+ p ESI Full ms
[80.00-1200.00] MS
niao-sample-p-2
9.57852
10.30377
1.00444
11.73816
19.03225 23.60451
Relative Abundance
Time (min)

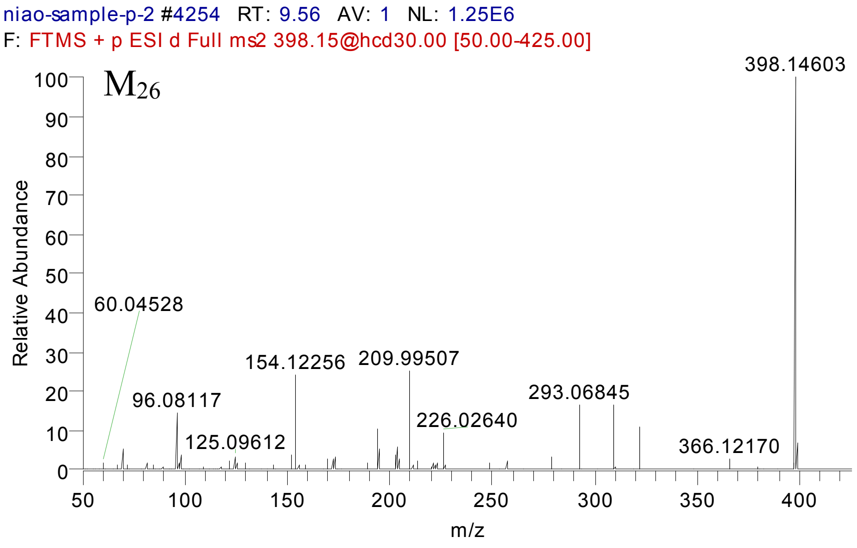
niao-sample-p-2 #4254 RT: 9.56 AV: 1 NL: 1.25E6
F: FTMS + p ESI d Full ms2 398.15@hcd30.00 [50.00-425.00]
M26
398.14603
60.04528
154.12256 209.99507
96.08117
226.02640
293.06845
125.09612
366.12170
Relative Abundance
m/z

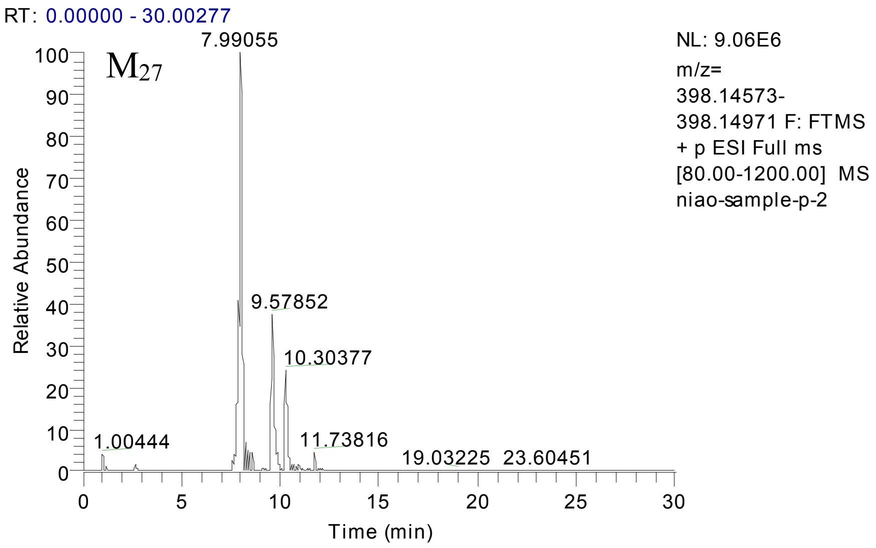
RT: 0.00000 - 30.00277
M27
7.99055
NL: 9.06E6
m/z=
398.14573-
398.14971 F: FTMS
+ p ESI Full ms
[80.00-1200.00] MS
niao-sample-p-2
9.57852
10.30377
1.00444
11.73816
19.03225 23.60451
Relative Abundance
Time (min)

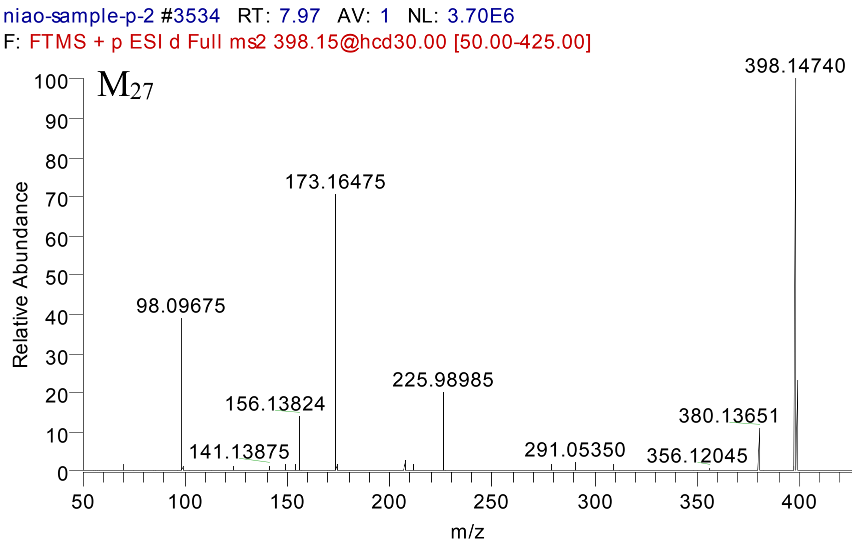
niao-sample-p-2 #3534 RT: 7.97 AV: 1 NL: 3.70E6
F: FTMS + p ESI d Full ms2 398.15@hcd30.00 [50.00-425.00]
M27
398.14740
173.16475
98.09675
225.98985
156.13824
380.13651
141.13875
291.05350
356.12045
Relative Abundance
m/z

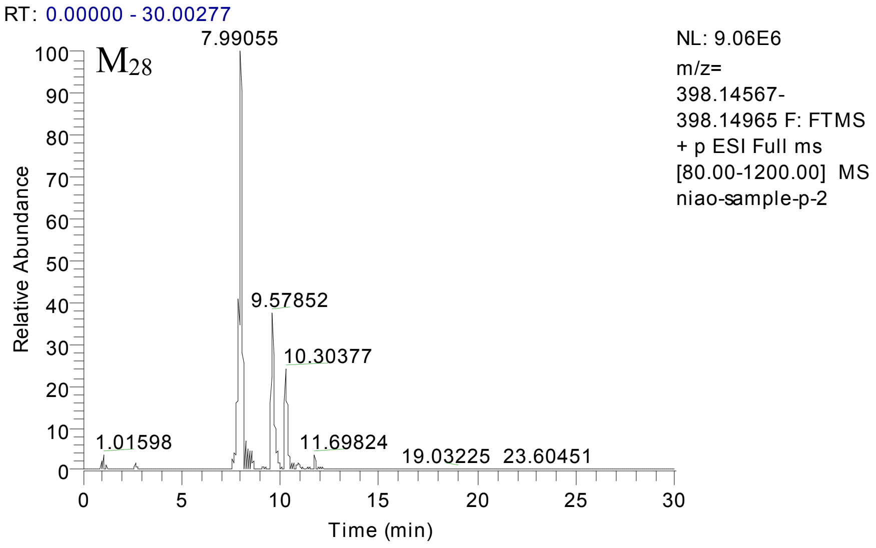

RT: 0.00000 - 30.00277
M28
7.99055
NL: 9.06E6
m/z=
398.14567-
398.14965 F: FTMS
+ p ESI Full ms
[80.00-1200.00] MS
niao-sample-p-2
9.57852
10.30377
1.01598
11.69824
19.03225
23.60451
Relative Abundance
Time (min)

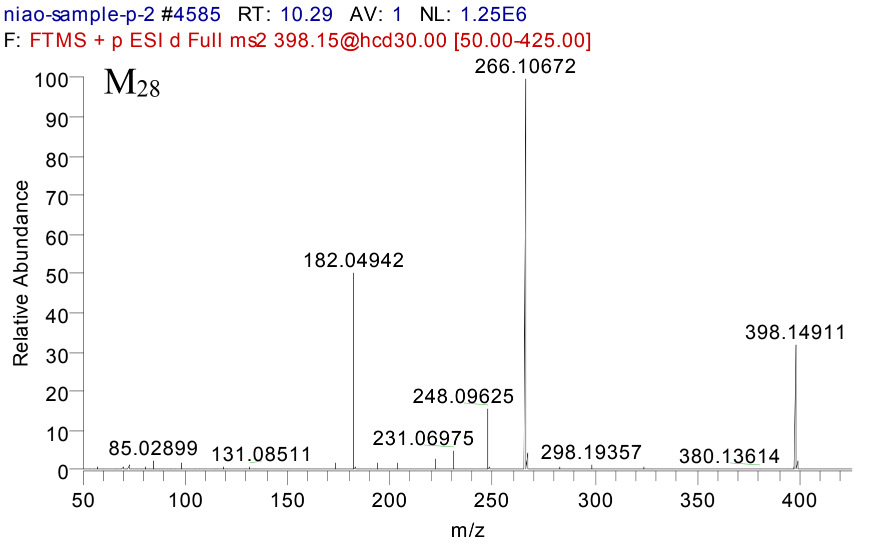

niao-sample-p-2 #4585 RT: 10.29 AV: 1 NL: 1.25E6
F: FTMS + p ESI d Full ms2 398.15@hcd30.00 [50.00-425.00]
M28
266.10672
182.04942
398.14911
248.09625
231.06975
85.02899
131.08511
298.19357
380.13614
Relative Abundance
m/z

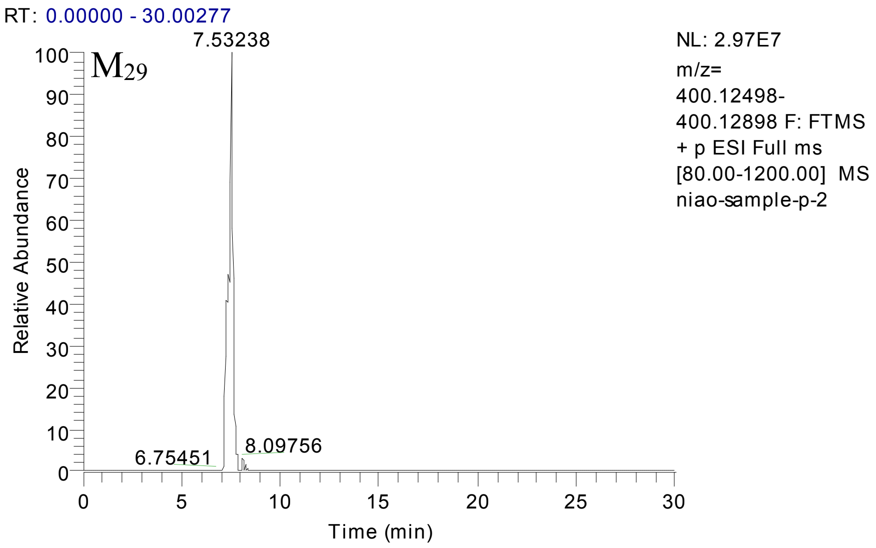

RT: 0.00000 - 30.00277
M29
7.53238
NL: 2.97E7
m/z=
400.12498-
400.12898 F: FTMS
+ p ESI Full ms
[80.00-1200.00] MS
niao-sample-p-2
6.75451
8.09756
Relative Abundance
Time (min)

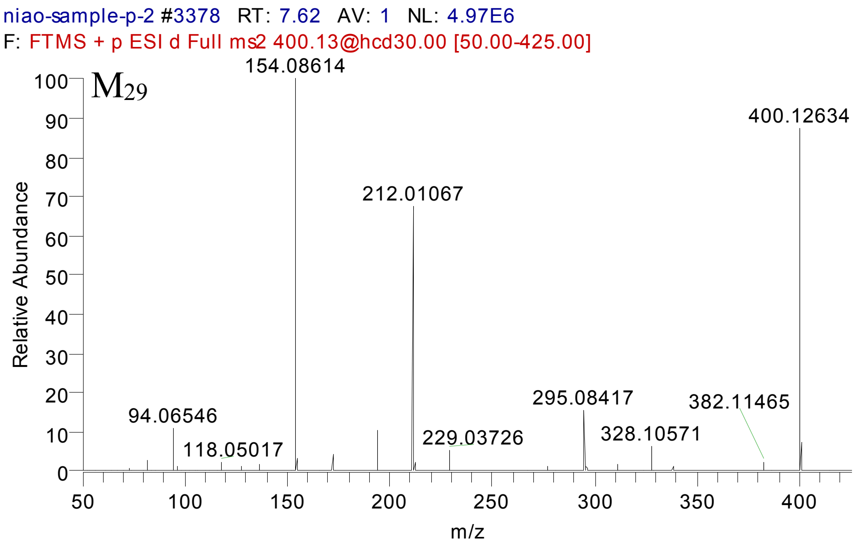

niao-sample-p-2 #3378 RT: 7.62 AV: 1 NL: 4.97E6
F: FTMS + p ESI d Full ms2 400.13@hcd30.00 [50.00-425.00]
M29
154.08614
400.12634
212.01067
295.08417
382.11465
94.06546
229.03726
328.10571
118.05017
Relative Abundance
m/z

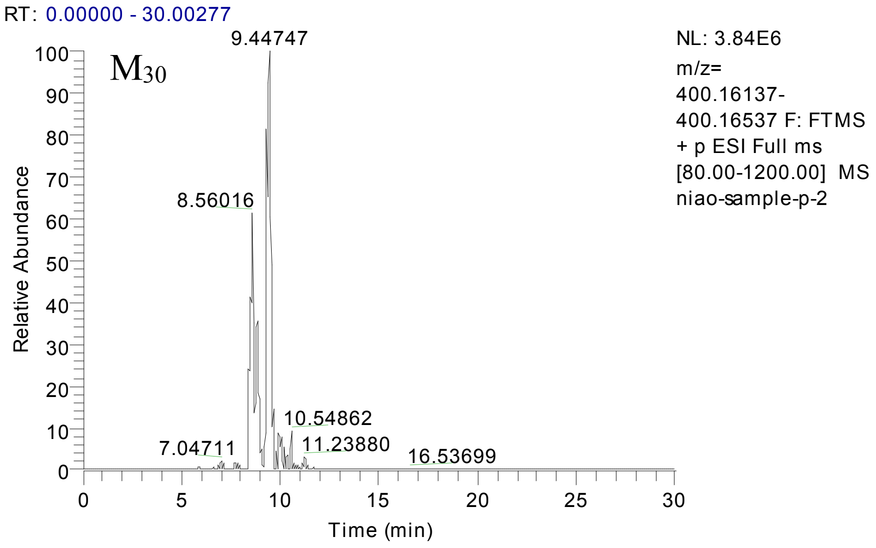
RT: 0.00000 - 30.00277
M30
9.44747
8.56016
7.04711
10.54862
11.23880
16.53699
NL: 3.84E6
m/z=
400.16137-
400.16537 F: FTMS
+ p ESI Full ms
[80.00-1200.00] MS
niao-sample-p-2
Relative Abundance
Time (min)

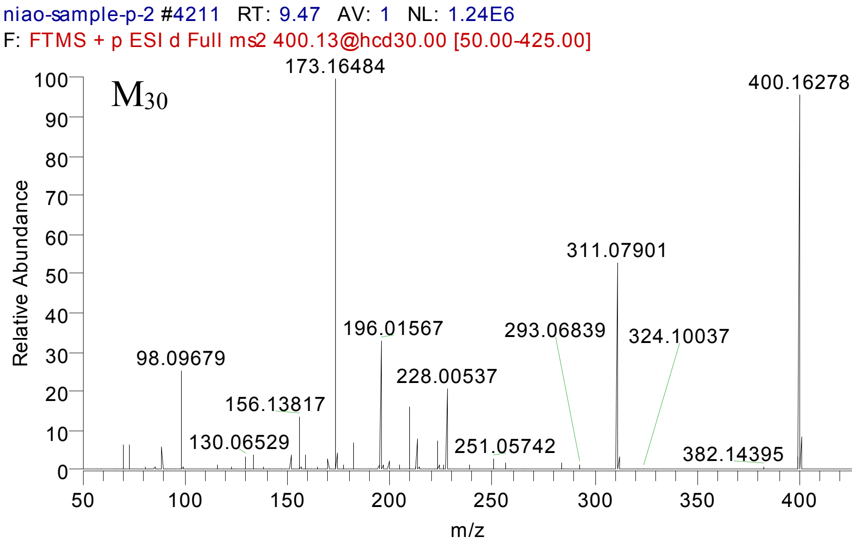
niao-sample-p-2 #4211 RT: 9.47 AV: 1 NL: 1.24E6
F: FTMS + p ESI d Full ms2 400.13@hcd30.00 [50.00-425.00]
M30
173.16484
400.16278
311.07901
196.01567
293.06839
324.10037
98.09679
228.00537
156.13817
130.06529
251.05742
382.14395
Relative Abundance
m/z

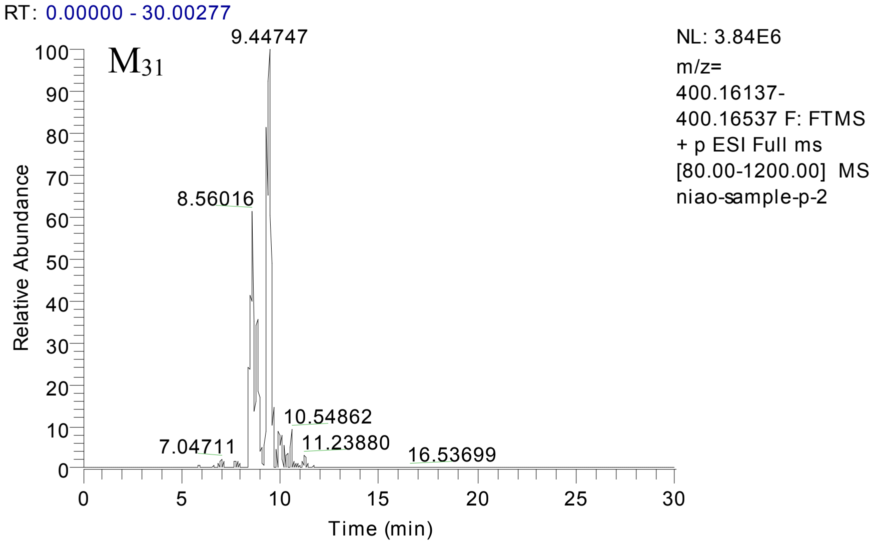
RT: 0.00000 - 30.00277
M31
9.44747
8.56016
7.04711
10.54862
11.23880
16.53699
NL: 3.84E6
m/z=
400.16137-
400.16537 F: FTMS
+ p ESI Full ms
[80.00-1200.00] MS
niao-sample-p-2
Relative Abundance
Time (min)

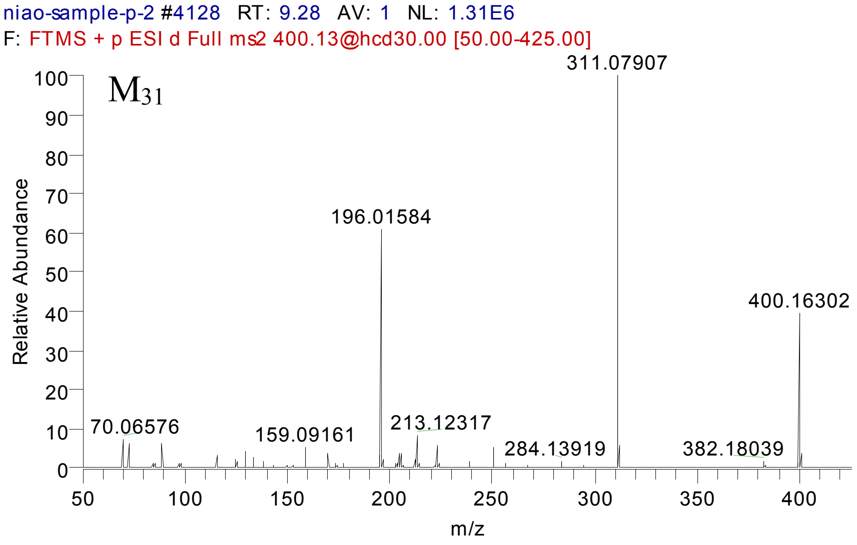
niao-sample-p-2 #4128 RT: 9.28 AV: 1 NL: 1.31E6
F: FTMS + p ESI d Full ms2 400.13@hcd30.00 [50.00-425.00]
M31
311.07907
196.01584
400.16302
70.06576
159.09161
213.12317
284.13919
382.18039
Relative Abundance
m/z

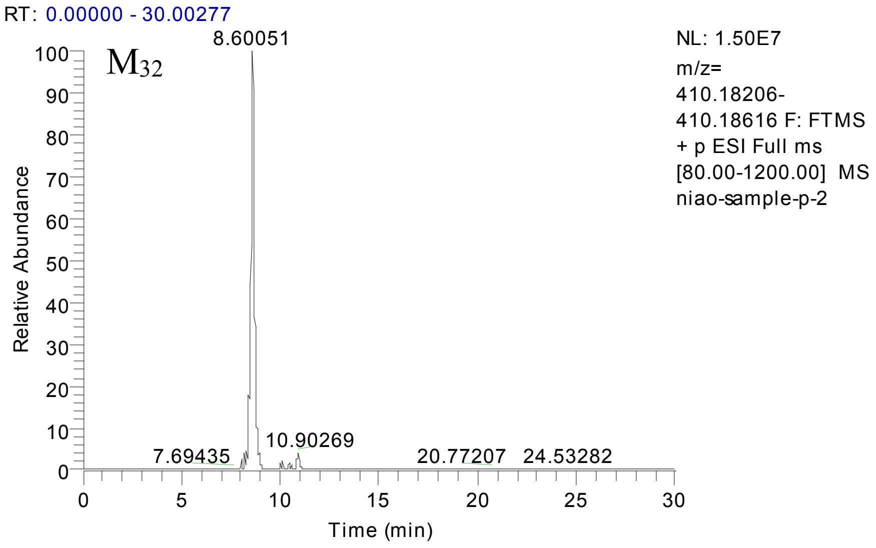

RT: 0.00000 - 30.00277
M32
8.60051
NL: 1.50E7
m/z=
410.18206-
410.18616 F: FTMS
+ p ESI Full ms
[80.00-1200.00] MS
niao-sample-p-2
7.69435
10.90269
20.77207
24.53282
Relative Abundance
Time (min)

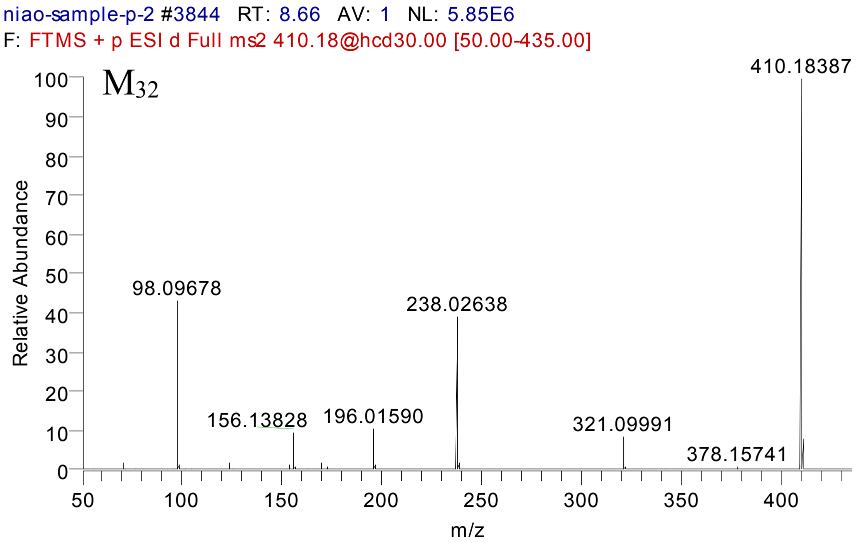

niao-sample-p-2 #3844 RT: 8.66 AV: 1 NL: 5.85E6
F: FTMS + p ESI d Full ms2 410.18@hcd30.00 [50.00-435.00]
M32
410.18387
98.09678
238.02638
156.13828
196.01590
321.09991
378.15741
Relative Abundance
m/z

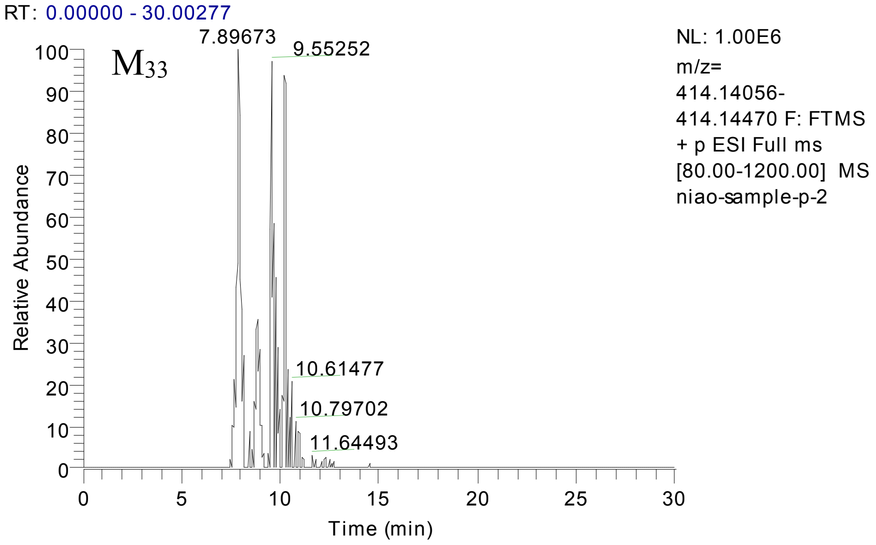

RT: 0.00000 - 30.00277
M33
7.89673
9.55252
NL: 1.00E6
m/z=
414.14056-
414.14470 F: FTMS
+ p ESI Full ms
[80.00-1200.00] MS
niao-sample-p-2
10.61477
10.79702
11.64493
Relative Abundance
Time (min)

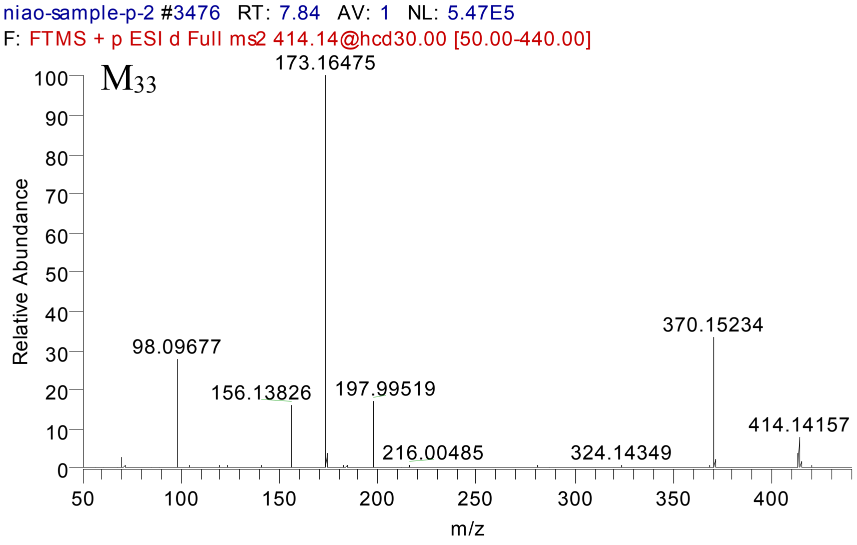

niao-sample-p-2 #3476 RT: 7.84 AV: 1 NL: 5.47E5
F: FTMS + p ESI d Full ms2 414.14@hcd30.00 [50.00-440.00]
M33
173.16475
370.15234
98.09677
156.13826
197.99519
414.14157
216.00485
324.14349
Relative Abundance
m/z

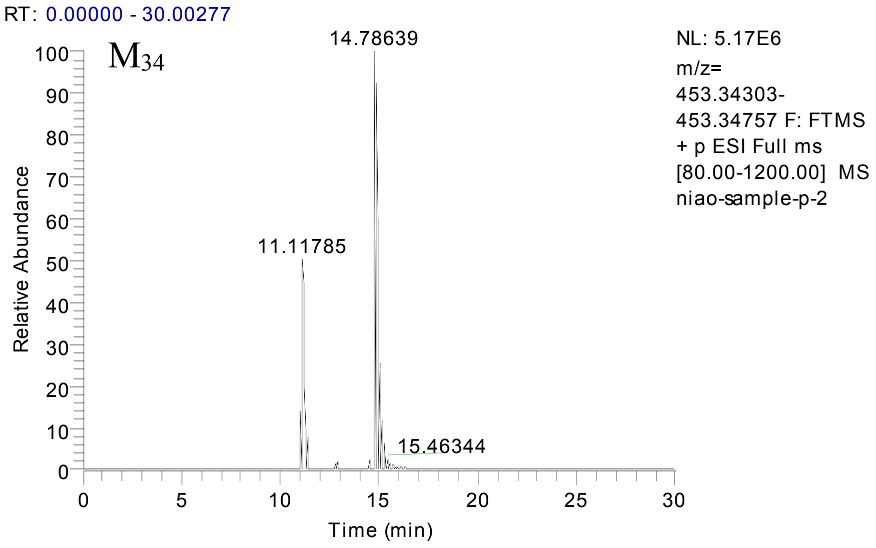
RT: 0.00000 - 30.00277
M34
14.78639
11.11785
15.46344
NL: 5.17E6
m/z=
453.34303-
453.34757 F: FTMS
+ p ESI Full ms
[80.00-1200.00] MS
niao-sample-p-2
Relative Abundance
Time (min)

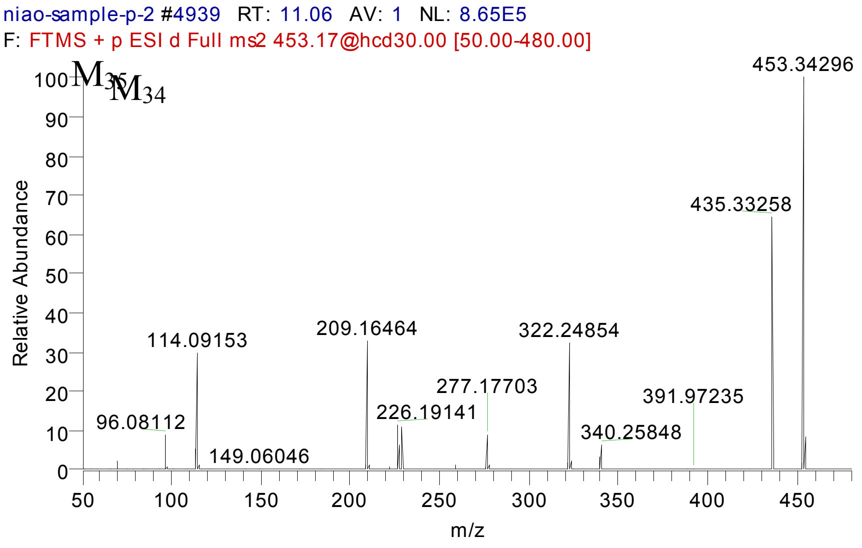
niao-sample-p-2 #4939 RT: 11.06 AV: 1 NL: 8.65E5
F: FTMS + p ESI d Full ms2 453.17@hcd30.00 [50.00-480.00]
M35
M34
453.34296
435.33258
114.09153
209.16464
322.24854
277.17703
391.97235
226.19141
96.08112
340.25848
149.06046
Relative Abundance
m/z

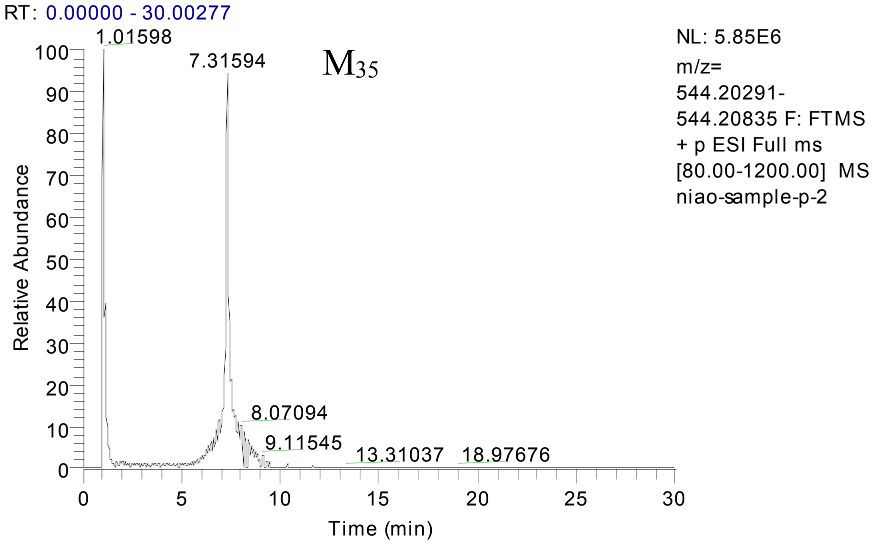
RT: 0.00000 - 30.00277
1.01598
7.31594
M35
8.07094
9.11545
13.31037
18.97676
NL: 5.85E6
m/z=
544.20291-
544.20835 F: FTMS
+ p ESI Full ms
[80.00-1200.00] MS
niao-sample-p-2
Relative Abundance
Time (min)

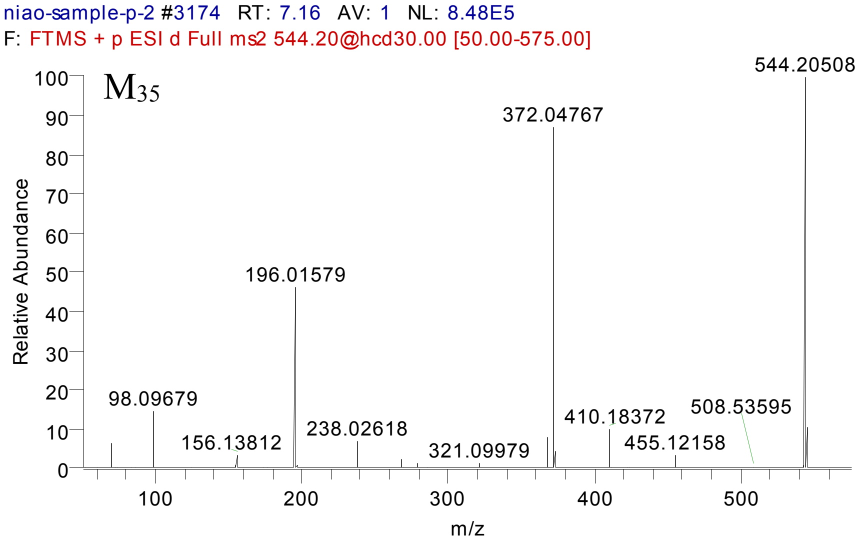
niao-sample-p-2 #3174 RT: 7.16 AV: 1 NL: 8.48E5
F: FTMS + p ESI d Full ms2 544.20@hcd30.00 [50.00-575.00]
M35
544.20508
372.04767
196.01579
98.09679
156.13812
238.02618
321.09979
410.18372
455.12158
508.53595
Relative Abundance
m/z

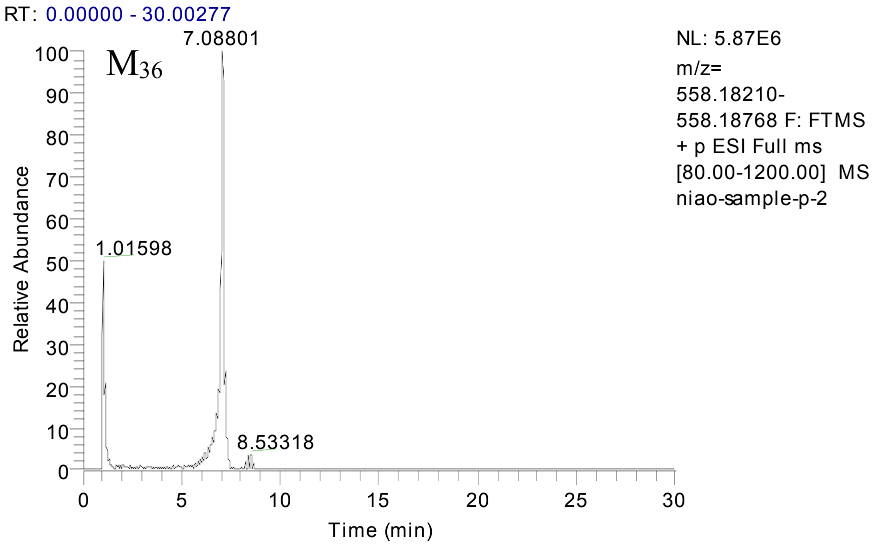
RT: 0.00000 - 30.00277
M36
7.08801
1.01598
8.53318
NL: 5.87E6
m/z=
558.18210-
558.18768 F: FTMS
+ p ESI Full ms
[80.00-1200.00] MS
niao-sample-p-2
Relative Abundance
Time (min)

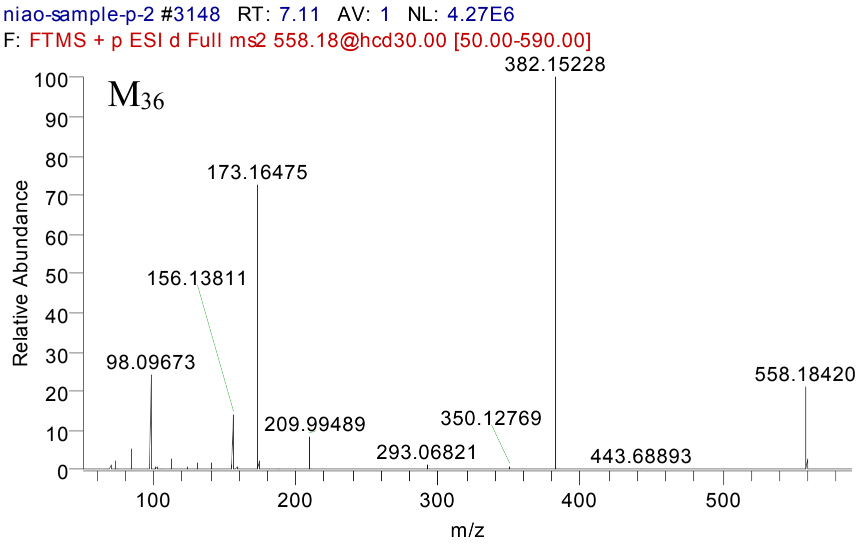
niao-sample-p-2 #3148 RT: 7.11 AV: 1 NL: 4.27E6
F: FTMS + p ESI d Full ms2 558.18@hcd30.00 [50.00-590.00]
M36
382.15228
173.16475
156.13811
98.09673
209.99489
350.12769
293.06821
443.68893
558.18420
Relative Abundance
m/z

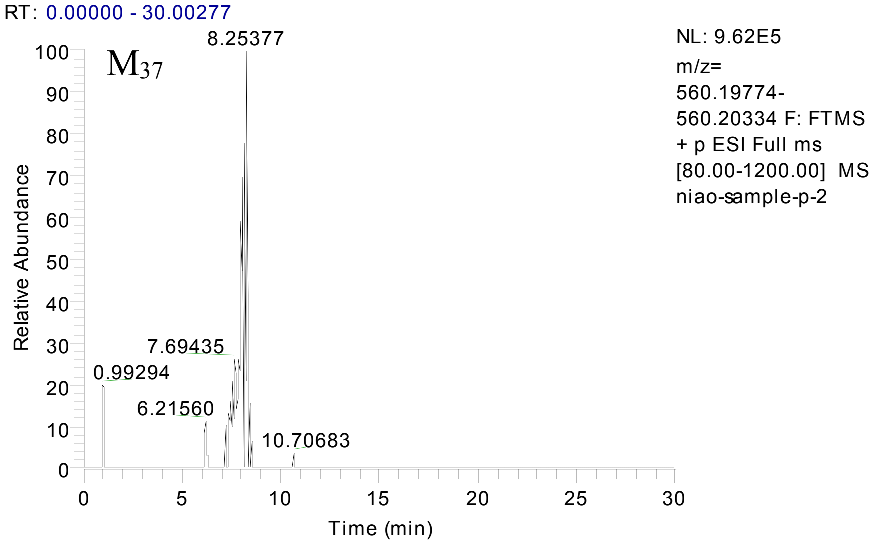
RT: 0.00000 - 30.00277
M37
8.25377
7.69435
0.99294
6.21560
10.70683
NL: 9.62E5
m/z=
560.19774-
560.20334 F: FTMS
+ p ESI Full ms
[80.00-1200.00] MS
niao-sample-p-2
Relative Abundance
Time (min)

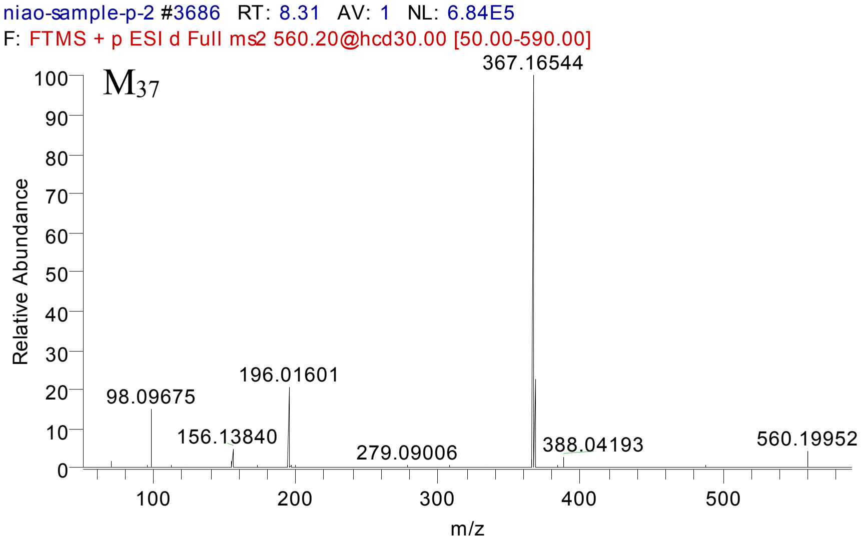
niao-sample-p-2 #3686 RT: 8.31 AV: 1 NL: 6.84E5
F: FTMS + p ESI d Full ms2 560.20@hcd30.00 [50.00-590.00]
M37
367.16544
196.01601
98.09675
156.13840
279.09006
388.04193
560.19952
Relative Abundance
m/z

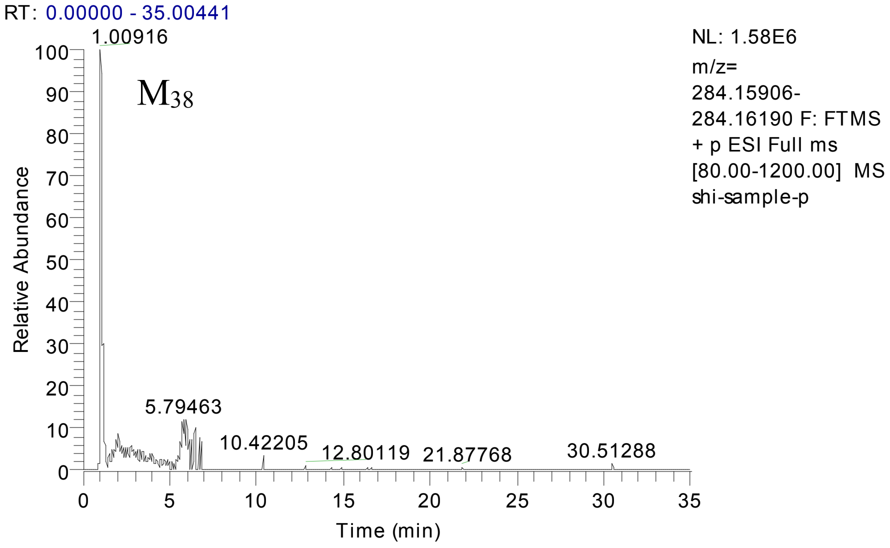
RT: 0.00000 - 35.00441
NL: 1.58E6
m/z=
284.15906-
284.16190 F: FTMS
+ p ESI Full ms
[80.00-1200.00] MS
shi-sample-p
M38
1.00916
5.79463
10.42205
12.80119
21.87768
30.51288
Relative Abundance
Time (min)

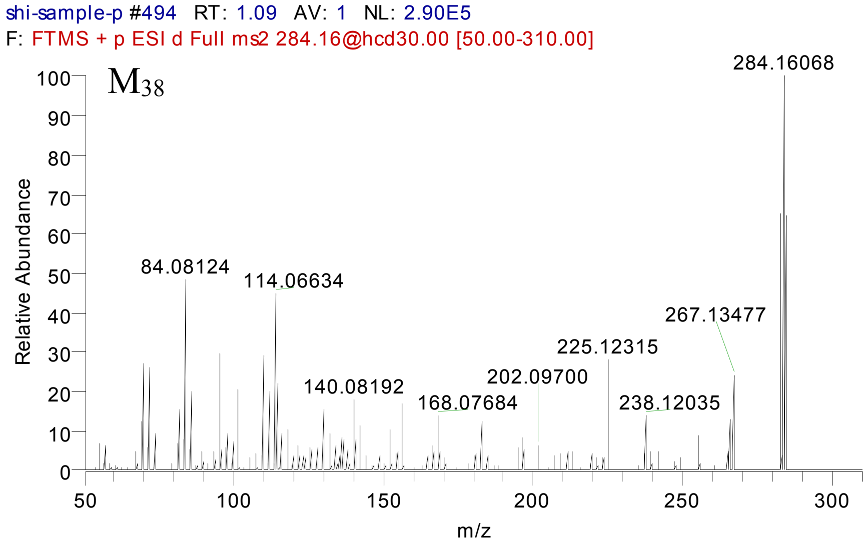
shi-sample-p #494 RT: 1.09 AV: 1 NL: 2.90E5
F: FTMS + p ESI d Full ms2 284.16@hcd30.00 [50.00-310.00]
M38
284.16068
84.08124
114.06634
267.13477
225.12315
202.09700
140.08192
168.07684
238.12035
Relative Abundance
m/z

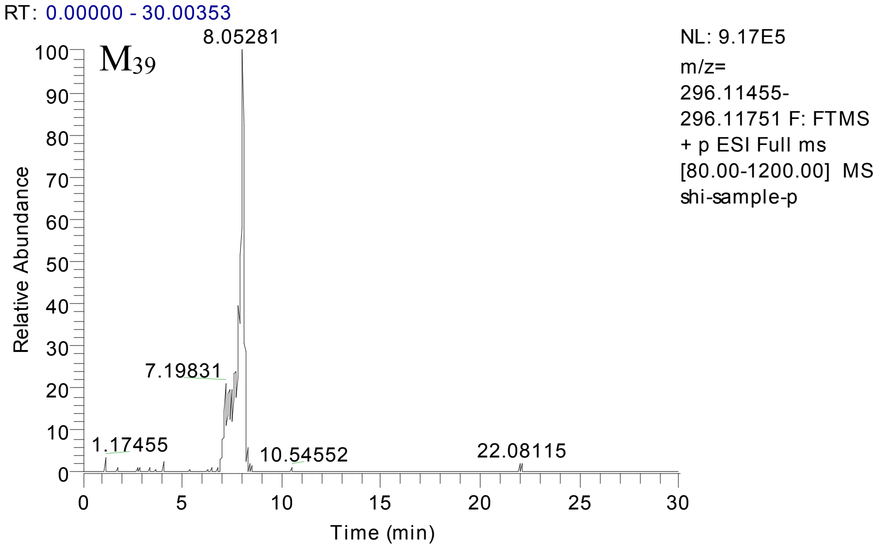
RT: 0.00000 - 30.00353
NL: 9.17E5
m/z=
296.11455-
296.11751 F: FTMS
+ p ESI Full ms
[80.00-1200.00] MS
shi-sample-p
M39
8.05281
7.19831
1.17455
10.54552
22.08115
Relative Abundance
Time (min)

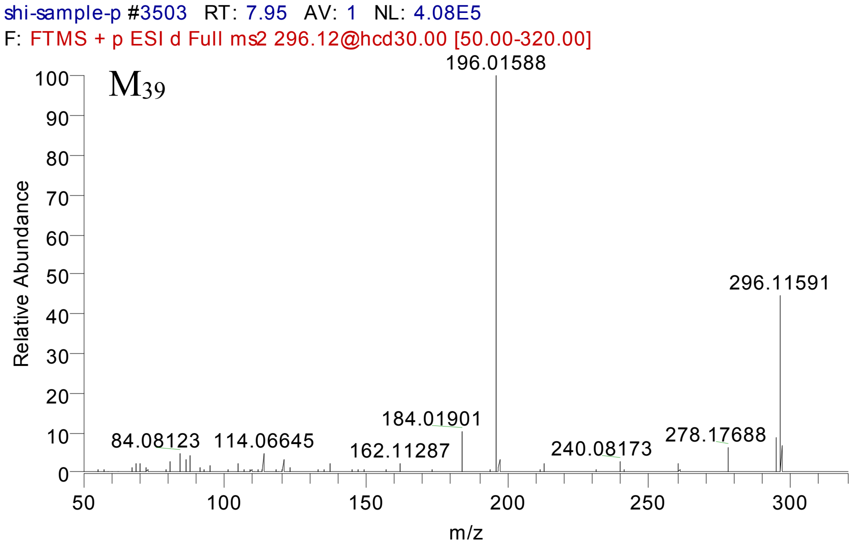
shi-sample-p #3503 RT: 7.95 AV: 1 NL: 4.08E5
F: FTMS + p ESI d Full ms2 296.12@hcd30.00 [50.00-320.00]
M39
196.01588
296.11591
184.01901
84.08123
114.06645
162.11287
240.08173
278.17688
Relative Abundance
m/z

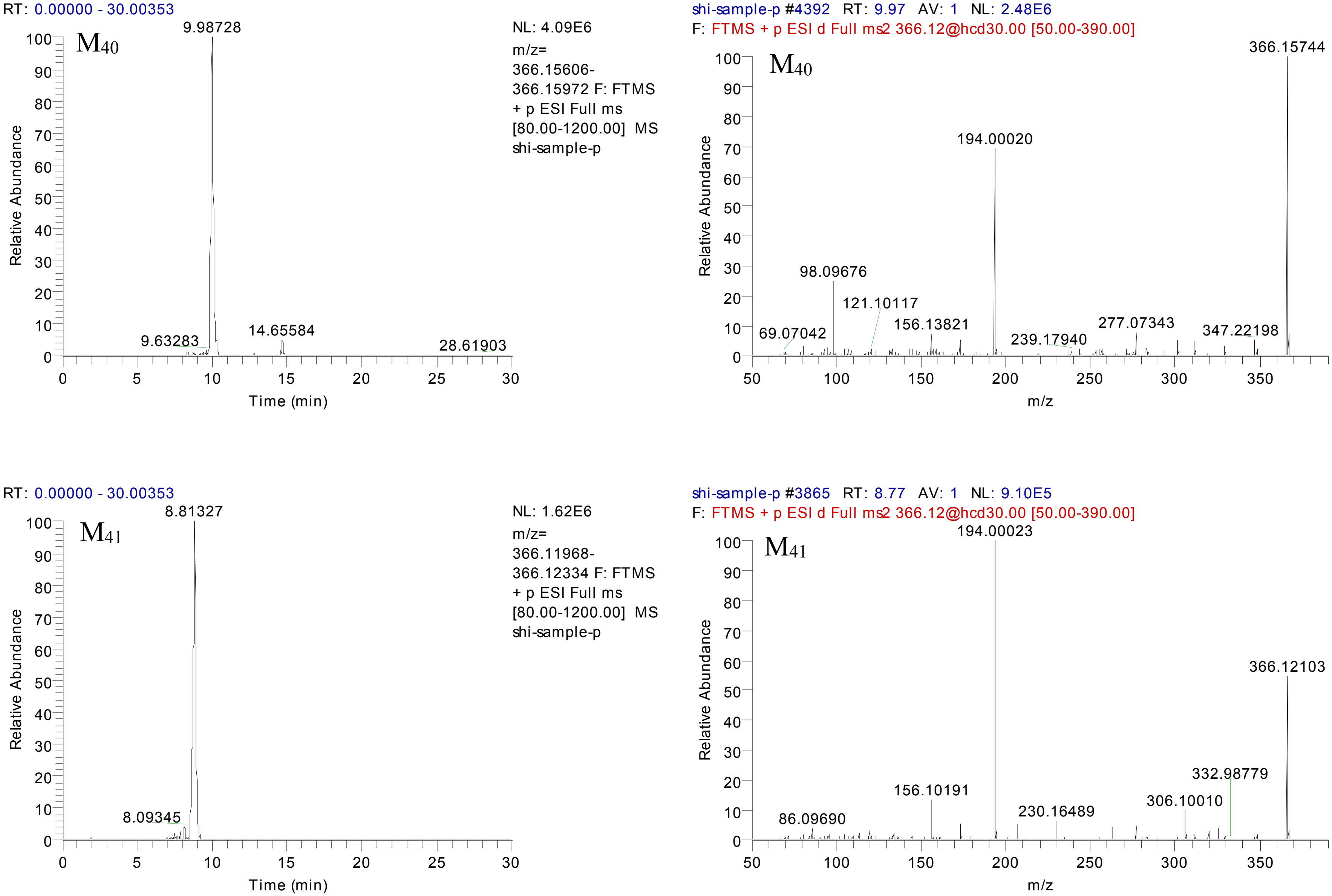
RT: 0.00000 - 30.00353
M40
9.98728
NL: 4.09E6
m/z=
366.15606-
366.15972 F: FTMS
+ p ESI Full ms
[80.00-1200.00] MS
shi-sample-p
9.63283
14.65584
28.61903
Relative Abundance
Time (min)
shi-sample-p #4392 RT: 9.97 AV: 1 NL: 2.48E6
F: FTMS + p ESI d Full ms2 366.12@hcd30.00 [50.00-390.00]
366.15744
194.00020
98.09676
121.10117
69.07042
156.13821
239.17940
277.07343
347.22198
m/z
RT: 0.00000 - 30.00353
M41
8.81327
NL: 1.62E6
m/z=
366.11968-
366.12334 F: FTMS
+ p ESI Full ms
[80.00-1200.00] MS
shi-sample-p
8.09345
shi-sample-p #3865 RT: 8.77 AV: 1 NL: 9.10E5
F: FTMS + p ESI d Full ms2 366.12@hcd30.00 [50.00-390.00]
194.00023
366.12103
332.98779
156.10191
306.10010
230.16489
86.09690

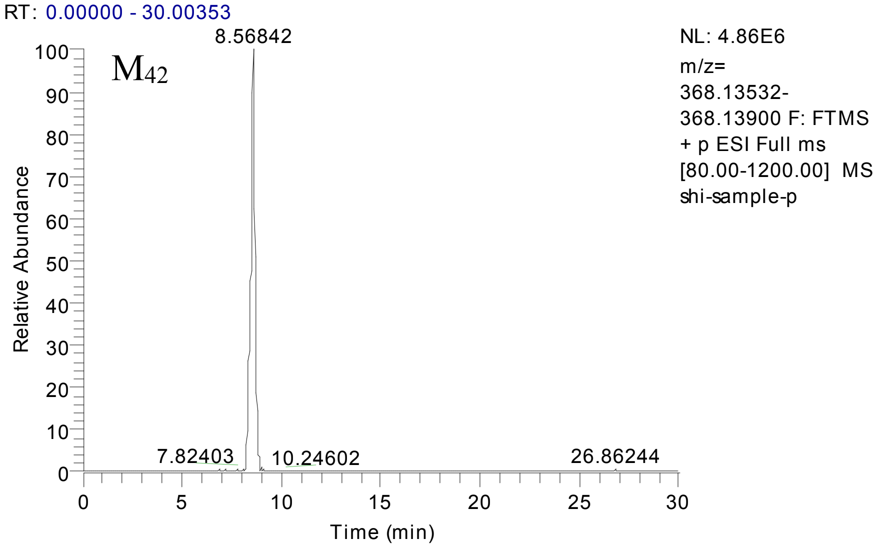
RT: 0.00000 - 30.00353
M42
8.56842
NL: 4.86E6
m/z=
368.13532-
368.13900 F: FTMS
+ p ESI Full ms
[80.00-1200.00] MS
shi-sample-p
7.82403
10.24602
26.86244
Relative Abundance
Time (min)

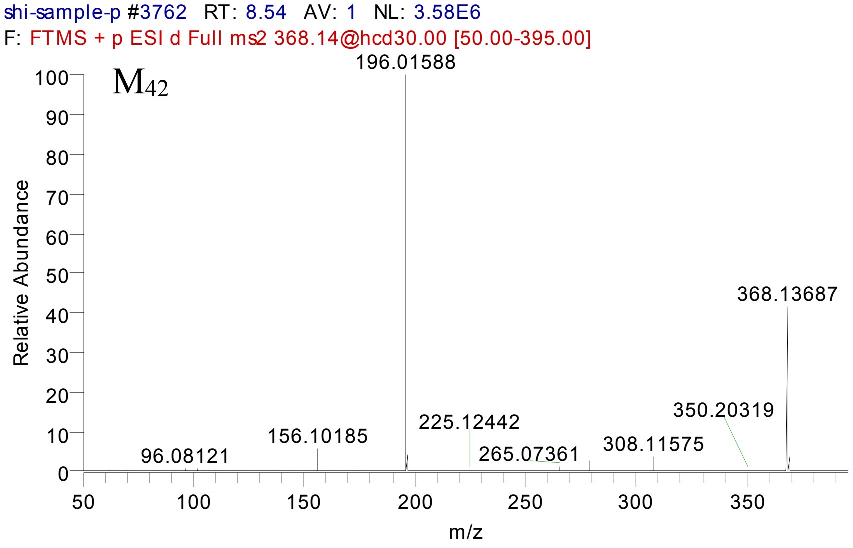
shi-sample-p #3762 RT: 8.54 AV: 1 NL: 3.58E6
F: FTMS + p ESI d Full ms2 368.14@hcd30.00 [50.00-395.00]
M42
196.01588
368.13687
350.20319
225.12442
156.10185
265.07361
308.11575
96.08121
Relative Abundance
m/z

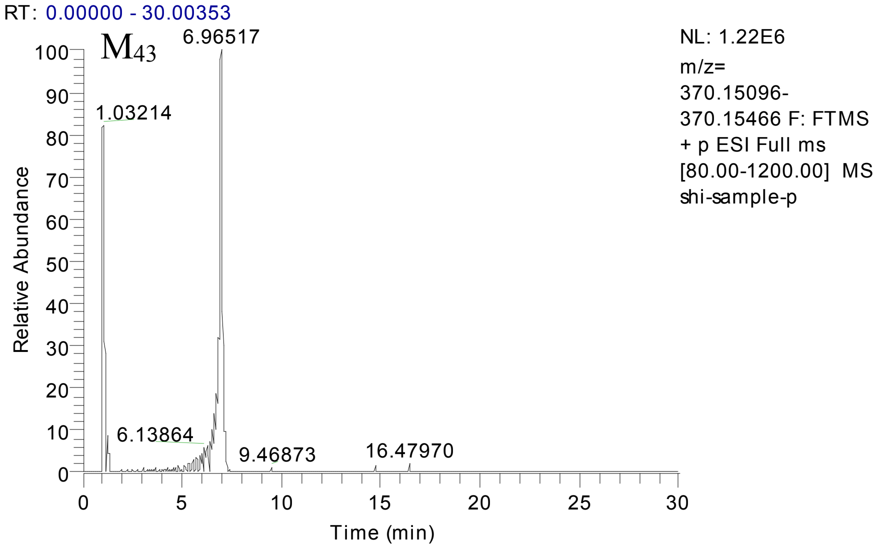
RT: 0.00000 - 30.00353
M43
6.96517
NL: 1.22E6
m/z=
370.15096-
370.15466 F: FTMS
+ p ESI Full ms
[80.00-1200.00] MS
shi-sample-p
1.03214
6.13864
9.46873
16.47970
Relative Abundance
Time (min)

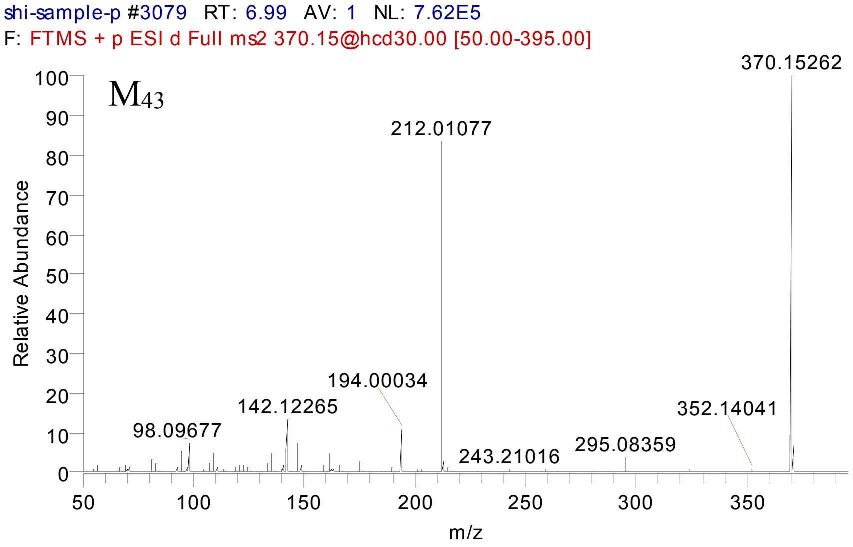
shi-sample-p #3079 RT: 6.99 AV: 1 NL: 7.62E5
F: FTMS + p ESI d Full ms2 370.15@hcd30.00 [50.00-395.00]
M43
370.15262
212.01077
194.00034
142.12265
352.14041
98.09677
295.08359
243.21016
Relative Abundance
m/z

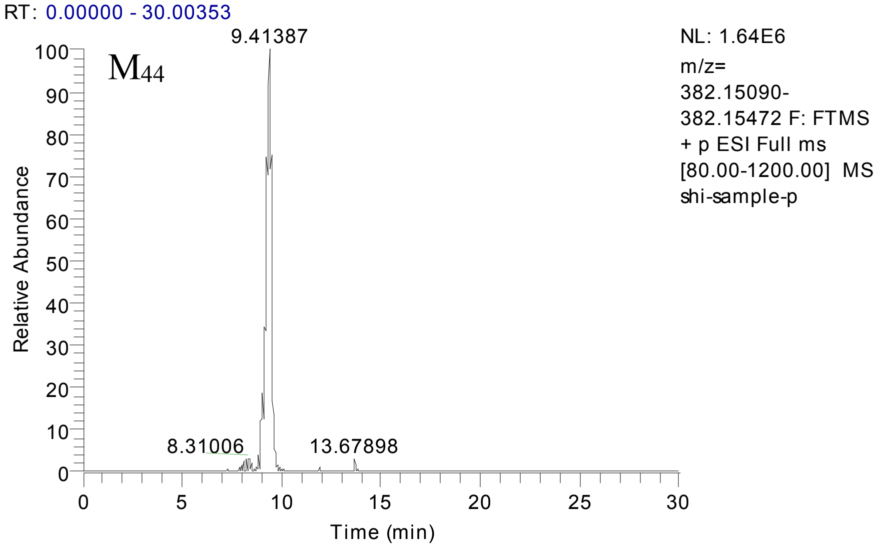
RT: 0.00000 - 30.00353
NL: 1.64E6
m/z= 382.15090-382.15472 F: FTMS + p ESI Full ms [80.00-1200.00] MS shi-sample-p
M44
9.41387
8.31006
13.67898
Relative Abundance
Time (min)

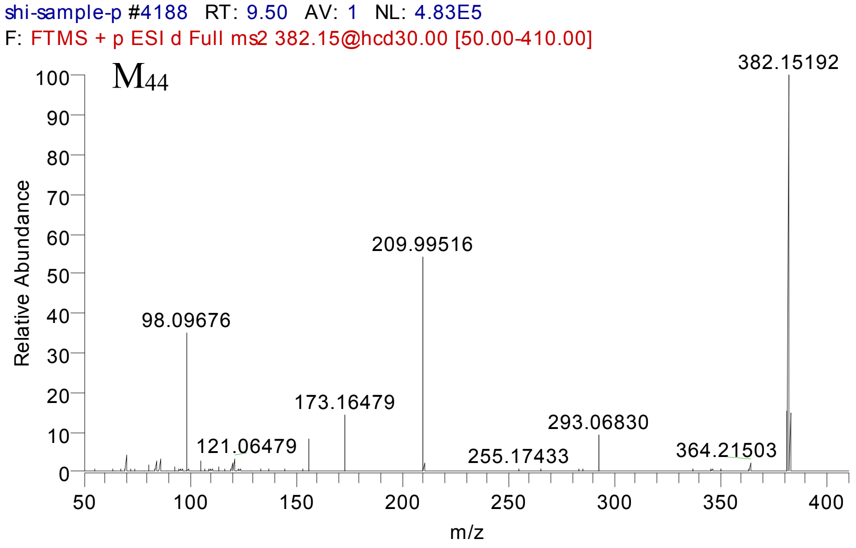
shi-sample-p #4188 RT: 9.50 AV: 1 NL: 4.83E5
F: FTMS + p ESI d Full ms2 382.15@hcd30.00 [50.00-410.00]
M44
382.15192
209.99516
98.09676
173.16479
293.06830
121.06479
255.17433
364.21503
Relative Abundance
m/z

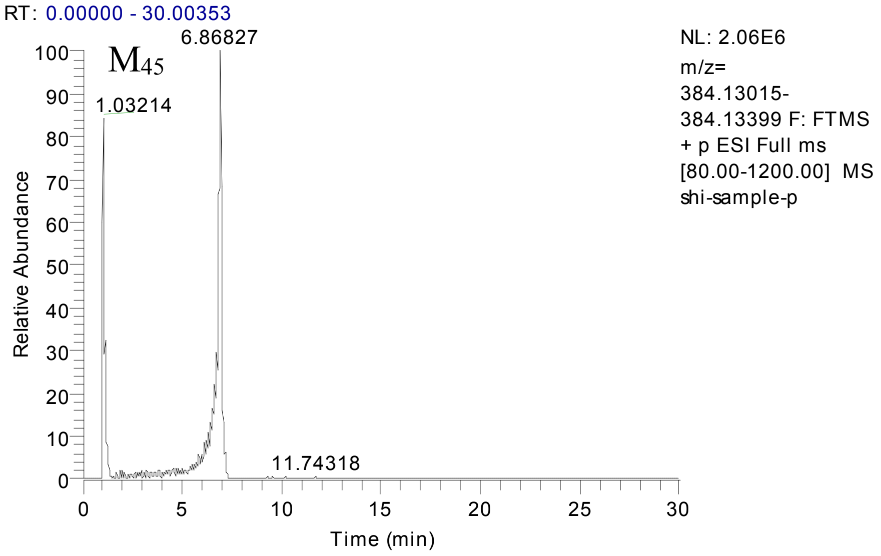
RT: 0.00000 - 30.00353
NL: 2.06E6
m/z= 384.13015-384.13399 F: FTMS + p ESI Full ms [80.00-1200.00] MS shi-sample-p
M45
6.86827
1.03214
11.74318
Relative Abundance
Time (min)

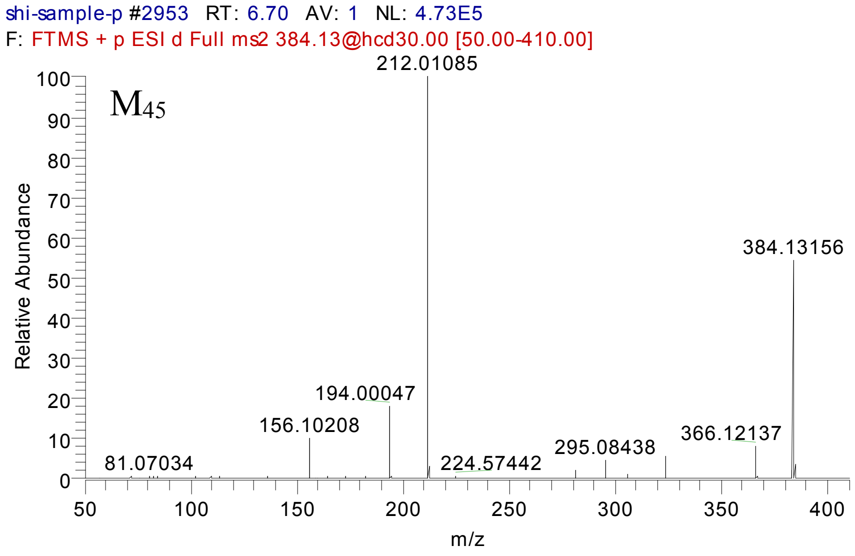
shi-sample-p #2953 RT: 6.70 AV: 1 NL: 4.73E5
F: FTMS + p ESI d Full ms2 384.13@hcd30.00 [50.00-410.00]
M45
212.01085
384.13156
194.00047
156.10208
366.12137
295.08438
81.07034
224.57442
Relative Abundance
m/z

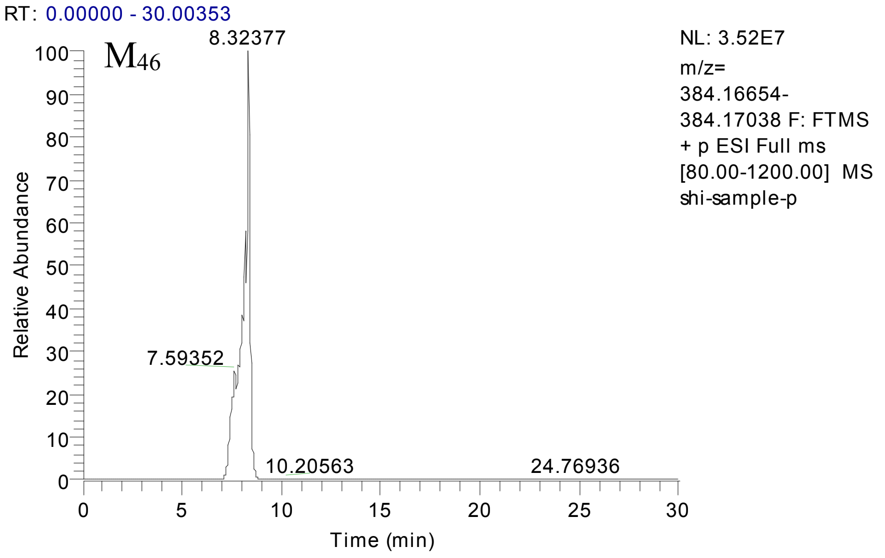
RT: 0.00000 - 30.00353
M46
8.32377
NL: 3.52E7
m/z=
384.16654-
384.17038 F: FTMS
+ p ESI Full ms
[80.00-1200.00] MS
shi-sample-p
7.59352
10.20563
24.76936
Relative Abundance
Time (min)

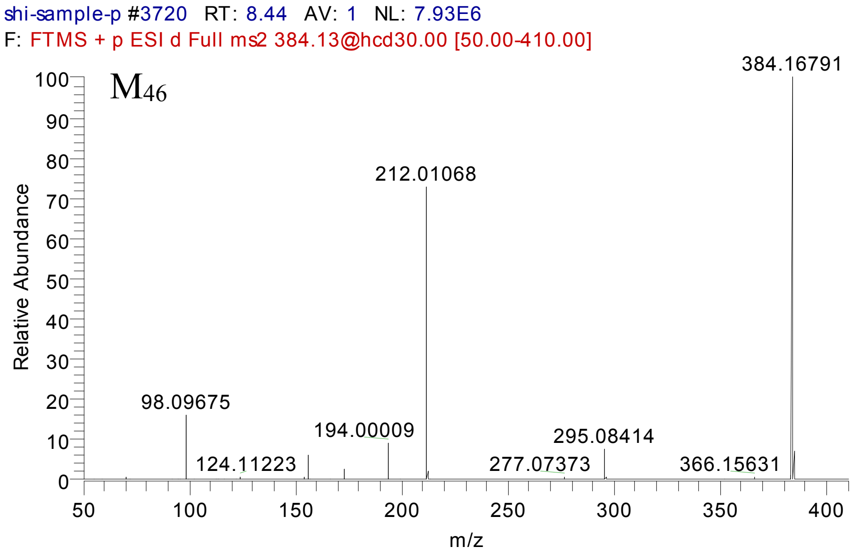
shi-sample-p #3720 RT: 8.44 AV: 1 NL: 7.93E6
F: FTMS + p ESI d Full ms2 384.13@hcd30.00 [50.00-410.00]
M46
384.16791
212.01068
98.09675
194.00009
295.08414
124.11223
277.07373
366.15631
Relative Abundance
m/z

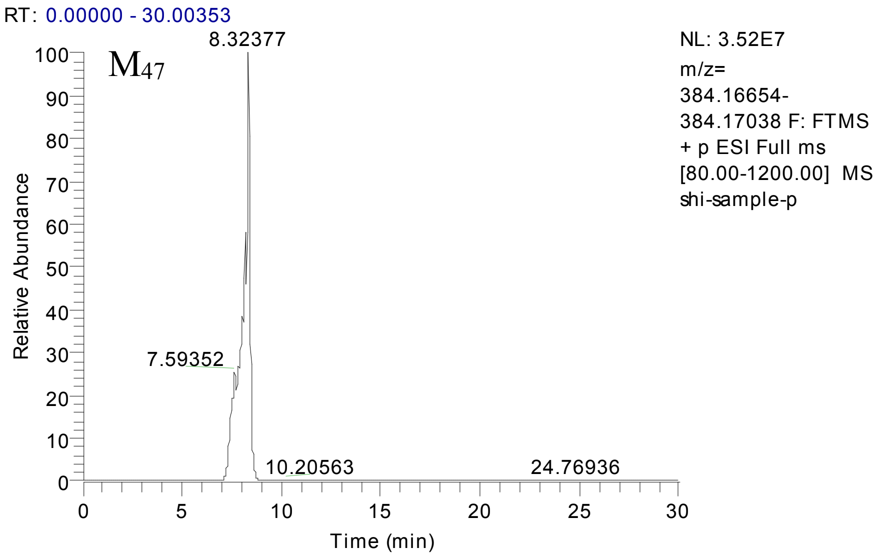
RT: 0.00000 - 30.00353
M47
8.32377
NL: 3.52E7
m/z=
384.16654-
384.17038 F: FTMS
+ p ESI Full ms
[80.00-1200.00] MS
shi-sample-p
7.59352
10.20563
24.76936
Relative Abundance
Time (min)

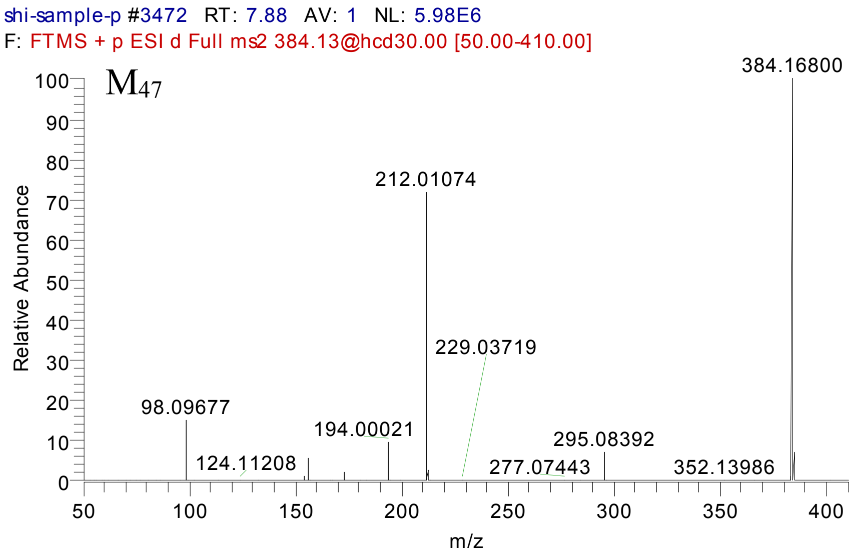
shi-sample-p #3472 RT: 7.88 AV: 1 NL: 5.98E6
F: FTMS + p ESI d Full ms2 384.13@hcd30.00 [50.00-410.00]
M47
384.16800
212.01074
229.03719
98.09677
194.00021
295.08392
124.11208
277.07443
352.13986
Relative Abundance
m/z
